# Solving the clustered traveling salesman problem *via* traveling salesman problem methods

**DOI:** 10.7717/peerj-cs.972

**Published:** 2022-06-13

**Authors:** Yongliang Lu, Jin-Kao Hao, Qinghua Wu

**Affiliations:** 1 School of Economics and Management, Fuzhou University, Fuzhou, China; 2 LERIA, Université d’Angers, Angers, France; 3 School of Management, Huazhong University of Science and Technology, Wuhan, China

**Keywords:** Traveling salesman, Heuristics, Clustered traveling salesman, Problem transformation

## Abstract

The Clustered Traveling Salesman Problem (CTSP) is a variant of the popular Traveling Salesman Problem (TSP) arising from a number of real-life applications. In this work, we explore a transformation approach that solves the CTSP by converting it to the well-studied TSP. For this purpose, we first investigate a technique to convert a CTSP instance to a TSP and then apply powerful TSP solvers (including exact and heuristic solvers) to solve the resulting TSP instance. We want to answer the following questions: How do state-of-the-art TSP solvers perform on clustered instances converted from the CTSP? Do state-of-the-art TSP solvers compete well with the best performing methods specifically designed for the CTSP? For this purpose, we present intensive computational experiments on various benchmark instances to draw conclusions.

## Introduction

The Clustered Traveling Salesman Problem (CTSP), originally proposed by Chisman (1975), is an extension of the classic Traveling Salesman Problem (TSP) where the cities are grouped into clusters and the cities of each cluster must be visited contiguously. Formally, the problem is defined on a symmetric complete weighted graph *G* = (*V*, *E*) with a set of vertices *V* = {1,2,…,*n*} and a set of edges *E* = {(*i*, *j*):*i*, *j* ∈ *V*, *i* ≠ *j*}. The vertex set *V* is partitioned into disjoint clusters *V*_1_,*V*_2_,…,*V*_*m*_ (*V*_1_ ∪ *V*_2_ ∪…∪ *V*_*m*_ = *V*). Let *C* be an *n* × *n* symmetric distance matrix such that *c*_*ij*_ (*i*, *j* = 1,2…,*n*, *i* ≠ *j*) represents the travel cost between two corresponding vertices *i* and *j*, and satisfies the triangle inequality rule. The objective of the CTSP is to find a minimum cost Hamiltonian circuit over all the vertices, where the vertices of each cluster must be visited consecutively.

**Figure 1:**
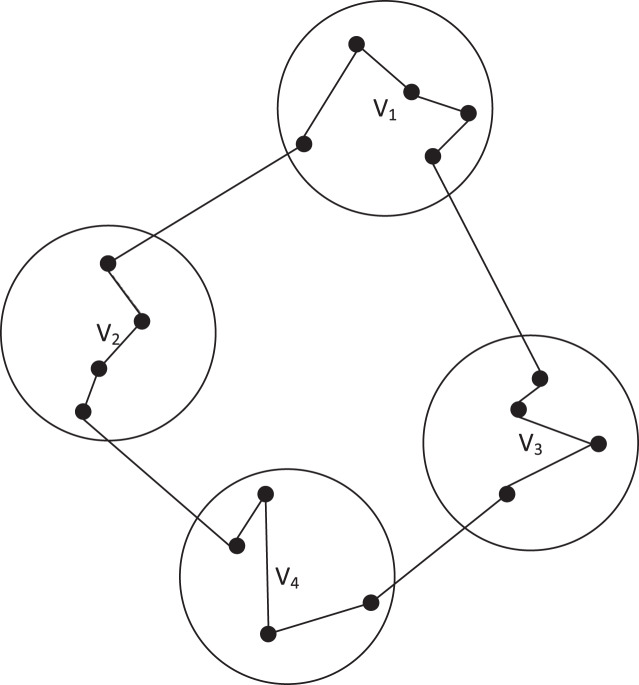
A feasible solution for an instance of the CTSP.

Figure 1 shows a feasible solution for a CTSP instance, where the solution corresponds to a Hamiltonian cycle such that the vertices of each cluster are visited contiguously.

The CTSP can be formally modelled as the following integer programming model described in Chisman (1975) where, without loss of generality, the salesman is assumed to leave origin city 1 and return to 1.

(1) $$\min f = \sum\limits_{i = 1}^n \sum\limits_{j = 1}^n {c_{ij}}{x_{ij}}$$

subject to

(2) $$\sum\limits_{j = 1}^n {x_{ij}} = 1\quad \quad \forall i \in V$$

(3) $$\sum\limits_{i = 1}^n {x_{ij}} = 1\quad \quad \forall j \in V$$

(4) $${u_i} - {u_j} + (n - 1){x_{ij}} \le n - 2\quad \quad 2 \le i \ne j \le n$$

(5) $$\sum\limits_{i \in {V_k}} \sum\limits_{j \in {V_k}} {x_{ij}} = |{V_k}| - 1\quad \quad \forall {V_k} \subset V,|{V_k}| \ge 1,k = 1,2,...,m$$

(6) $${x_{ij}} \in \{ 0,1\} \quad \quad \forall i,j \in V$$

(7) $${u_i} \ge 0\quad \quad 2 \le i \le n$$

In this model, the binary variable *x*_*ij*_ = 1 if city *j* is visited immediately after city *i*; *x*_*ij*_ = 0 otherwise. Objective function (1) seeks to minimize the total distance traveled by the salesman. Constraints (2) and (3) ensure that each city is visited exactly once. Constraints (4) eliminate subtours, while constraints (5) guarantee that the cities of each cluster are visited contiguously. The remaining constraints are related to the decision variables.

The above subtour elimination constraints (4) are called MTZ formulation (Miller, Tucker & Zemlin, 1960). Although MTZ is simple to implement, it provides a very poor linear relaxation (Campuzano, Obreque & Aguayo, 2020). Many compact formulations have been proposed to replace Constraints (4). According to the literature, a multi-commodity flow formulation (Wong, 1980; Claus, 1984) was proven to provide a strong linear relaxation, without compromising its simplicity. In the multi-commodity flow formulation, let *k* = 2,3,…,*n* be *n* − 1 commodities, and let $y_{ij}^k$ be a nonnegative decision variable which represents the flow on the arc (*i*, *j*)∈ *E* for the commodity *k* from city 1 to city *k*. Then, another alternative mathematical model for the CTSP is constituted of the objective function (1) and the constraints (2), (3), (5), (6) along with the following subtour elimination constraints:

(8) $$0 \le y_{ij}^k \le {x_{ij}}\quad \quad \forall i,j,k \in V,k \ne 1$$

(9) $$\sum\limits_{i = 2}^n y_{1i}^k = 1\quad \quad \forall k \in V\backslash \{ 1\}$$

(10) $$\sum\limits_{i = 2}^n y_{i1}^k = 0\quad \quad \forall k \in V\backslash \{ 1\}$$

(11) $$\sum\limits_{i = 1}^n y_{ik}^k = 1\quad \quad \forall k \in V\backslash \{ 1\}$$

(12) $$\sum\limits_{j = 1}^n y_{kj}^k = 0\quad \quad \forall k \in V\backslash \{ 1\}$$

(13) $$\sum\limits_{i = 1}^n y_{ij}^k - \sum\limits_{i = 1}^n y_{ji}^k = 0\quad \quad \forall j,k \in V\backslash \{ 1\} ,j \ne k$$

Constraints (8) only allow flow in an arc (*i*, *j*) if and only if it is traversed by the salesman (*i.e*., *x*_*ij*_ = 1). Constraints (9) ensure that city 1 is the source of one unit of each commodity *k* ∈ *V* \{1} and Constraints (10) avoid that the flow of each commodity *k* ∈ *V* \{1} returns to city 1. Constraints (11) and (12) guarantees that one flow unit of commodity *k* enters to city *k* and it does not leave the city *k*. Constraints (13) ensure flow conservation at each city, apart from city 1 and for commodity *k* at city *k*.

One notices that the CTSP is equivalent to the TSP when there is a single cluster or when each cluster contains exactly one vertex. Therefore, the CTSP is NP-hard, and thus computationally challenging in the general case. From a practical perspective, the CTSP is a versatile modeling tool for several operational research applications arising in a wide variety of areas, including automated warehouse routing (Chisman, 1975), emergency vehicle dispatching (Weintraub et al., 1999), production planning (Lokin, 1979), disk defragmentation (Laporte & Palekar, 2002), and commercial transactions with supermarkets, shops and grocery suppliers (Ghaziri & Osman, 2003). As a result, effective solution methods for the CTSP can help to solve these practical problems. Indeed, the computational challenge and the wide range of applications of the problem have motivated a variety of approaches that are reviewed in the “Literature Review on Existing Solution Methods” section. However, unlike the classic TSP problem for which many powerful methods have been introduced in the past decades, studies on the CTSP are still quite limited.

Moreover, the CTSP belongs to the large class of traveling salesman problems. Among the TSP variants, the generalized traveling salesman problem (GTSP) (Srivastava et al., 1969; Cosma, Pop & Cosma, 2021) and the family traveling salesman problem (FTSP) (Morán-Mirabal, Velarde & Resende, 2014; Pop, Matei & Pintea, 2018) share similarities with the CTSP. In the GTSP, the set of vertices is divided into clusters and the objective is to find a minimum-cost tour passing through one vertex from each cluster. In the FTSP, the set of vertices is also divided into clusters (called families) and the objective is to visit a predefined number of vertices in each family at a minimum cost.

In this work, we investigate the problem transformation approach proposed in Chisman (1975), which converts the CTSP to the TSP and assess the interest of popular modern TSP solvers for solving the resulting TSP instances. To our knowledge, this is the first large computational study testing modern TSP solvers on solving the CTSP. The work is motivated by the following considerations. First, intensive researches on the TSP have led to the development of many very powerful solvers. Thus, it is interesting to know whether we can take advantage of these solvers to effectively solve the CTSP. Second, the TSP instances converted from the CTSP are characterized by their cluster structures. These instances constitute interesting test cases for existing TSP solvers. This work aims thus to answer the following questions.

1. How do state-of-the-art *exact* TSP solvers perform on clustered instances converted from the CTSP?
2. How do state-of-the-art *inexact* (heuristic) TSP solvers perform on clustered instances converted from the CTSP?
3. Do state-of-the-art TSP solvers compete well with the best performing methods specifically designed for the CTSP?

To our knowledge, Questions 1 and 3 have never been investigated in the literature. Regarding Question 2, two previous studies (Neto, 1999; Helsgaun, 2014) are of interest. However, they are limited because they only concern one TSP algorithm, *i.e*., the local search based LKH solver (Helsgaun, 2009), while ignoring other powerful TSP solvers like GA-EAX (Nagata & Kobayashi, 1997) and Concorde (Applegate, Bixby & Chvatal, 2006). Answering these questions helps to enrich the state-of-the-art of solving the CTSP and gain novel knowledge on using modern TSP methods to solve new problems. Finally, we mention that the transformation approach was also tested in Lokin (1979) and Jongens & Volgenant (1985). However, these studies are clearly outdated and don’t provide useful information as to the questions we want to investigate.

The remainder of this paper is organized as follows. “Literature Review on Existing Solution Methods” reviews existing solution methods for the CTSP. “Solving the CTSP *via* TSP Methods” presents the transformation of the CTSP to the TSP and three powerful TSP methods (solvers). “Computational Experiments” shows computational studies of the TSP solvers applied to the clustered instances and comparisons with existing algorithms dedicated to the CTSP. “Discussion” provides additional explanations regarding the behaviors of the three TSP solvers. Finally, concluding remarks are provided in “Conclusion”.

## Literature review on existing solution methods

There are several dedicated solution algorithms for solving the CTSP that are based on exact, approximation, and metaheuristic approaches.

Along with the introduction of the CTSP, Chisman (1975) proposed a branch-and-bound algorithm to solve the integer programming model presented in the Introduction section. Jongens & Volgenant (1985) developed an algorithm based on the 1-tree relaxation to provide lower bounds as well as a heuristic to find satisfactory upper bounds. Mestria, Ochi & de Lima Martins (2013) used the mathematical formulation of Chisman (1975) and IBM Parallel CPLEX solver (version 11.2) to obtain lower bounds for medium CTSP instances (|*V*| ≤ 1,000).

Various *a*-approximation algorithms (Anily, Bramel & Hertz, 1999; Gendreau, Laporte & Hertz, 1997; Guttmann-Beck et al., 2000) have been developed for the CTSP. These approximation algorithms require either the starting and ending vertices in each cluster or a prespecified order of visiting the clusters in the tour as inputs, and solve the inter-cluster and intra-cluster problems independently. Bao & Liu (2012) presented a new 2.17-approximation algorithm where no starting and ending vertices were specified. Later, Bao et al. (2017) provided a 2.5-approximation algorithm for another version of the CTSP where the starting vertex of each cluster is given while the ending vertex is not specified. Recently, Kawasaki & Takazawa (2020) improved the approximation ratio for the CTSP by incorporating a recent approximation algorithm for the TSP by Zenklusen (2019).

Given that the CTSP is a NP-hard problem, a number of heuristic and metaheuristic algorithms have also been investigated, which aim to provide high-quality solutions in acceptable computation time, but without provable optimal guarantee of the attained solutions. For example, Laporte, Potvin & Quilleret (1997) presented a tabu search algorithm to solve a particular case of the CTSP, where the clusters are visited in a prespecified order. Potvin & Guertin (1996) developed a genetic algorithm for the CTSP that finds inter-cluster paths and then intra-cluster paths. Later, Ding, Cheng & He (2007) proposed a two-level genetic algorithm for the CTSP. In the first level, a genetic algorithm is used to find the shortest Hamiltonian cycle for each cluster. In the second level, a modified genetic algorithm is applied to merge the Hamiltonian cycles of all the clusters into a complete tour.

In addition to these early heuristic algorithms, Mestria, Ochi & de Lima Martins (2013) investigated GRASP (Greedy Randomized Adaptive Search Procedure) with path-relinking. Among the six proposed heuristics, one heuristic corresponds to the traditional GRASP procedure whereas the other heuristics include different path relinking procedures. Mestria (2016) studied a hybrid heuristic, which is based on a combination of GRASP, Iterated Local Search (ILS) and Variable Neighborhood Descent (VND). Recently, Mestria (2018) presented another complex hybrid algorithm (VNRDGILS) which mixes GRASP, ILS, and Variable Neighborhood Random Descent to explore several neighborhoods. According to the computational results reported in Mestria, Ochi & de Lima Martins (2013) and Mestria (2016, 2018), these GRASP-based algorithms are among the best performing heuristics specially designed for the CTSP currently available in the literature. In addition, Hà et al. (2022) proposed a metaheuristic method based on the ILS framework with problem-tailored operators for a version of the CTSP where the order of visiting the clusters is prespecified.

Existing studies have significantly contributed to better solving the CTSP. According to the computational results reported in the literature, due to the NP-hardness of the problem, only small CTSP instances were able to be solved to optimality with the exact algorithms. The approximation algorithms provide solutions for the CTSP within a given approximation factor. However, due to the high approximation factors involved (*e.g*., 5/3 (Anily, Bramel & Hertz, 1999), 3/2 Gendreau, Laporte & Hertz (1997), 2.17 (Bao & Liu, 2012), and 2.5 (Bao et al., 2017)), these approximation algorithms are not practical for solving large instances. To deal with large CTSP instances, heuristic and metaheuristic algorithms are often preferred to find sub-optimal solutions within an acceptable computation time.

## Solving the ctsp *via* tsp methods

### Transformation of the CTSP to the TSP

As the literature review shows, a number of dedicated solution approaches have been developed to solve the CTSP. However, one observes that these approaches have difficulty producing robustly and consistently high-quality solutions for large-scale CTSP instances with tens of thousands of vertices. Moreover, the best performing CTSP methods (*e.g*., VNRDGILS (Mestria, 2018), HHGILS (Mestria, 2016), and GPR1R2 (Mestria, Ochi & de Lima Martins, 2013)) are computationally expensive (*e.g*., requiring 1,080 s to find good solutions for instances with 1,173 ≤ *n* ≤ 2,000).

On the other hand, problem transformation has been highly successful in solving several difficult optimization problems such as the latin square completion problem *via* graph coloring (Jin & Hao, 2019) and the winner determination problem *via* weighted maximum cliques (Wu & Hao, 2015). It is known that the CTSP can be transformed to the conventional TSP (Chisman, 1975). Therefore, in principle, the CTSP can be solved by any TSP algorithm. However, to our knowledge, no computational study on using problem transformation to solve the CTSP has been presented in the literature. This work fills the gap by exploring the problem transformation approach of Chisman (1975) and testing three representative state-of-the-art TSP solvers including both exact and inexact solution approaches.

The basic idea of transforming the CTSP to the TSP is to add a large artificial cost *M* to all inter-cluster edges in order to force the salesman to visit all the cities within each cluster before leaving it.

Given a CTSP instance *G* = (*V*, *E*) with distance matrix *C*, we define a TSP instance *G′* = (*V′*, *E′*) with distance matrix *C′* as follow.

- Define *V* = *V′* and *E* = *E′*.
- Define the travel distance *c′*_*ij*_ in *G′* by

$c_{ij}^{\prime} = \left\{{\matrix{ {{c_{ij}} + M} & {{\rm if}\ i\ {\rm and}\ j\ {\rm belong\ to\ different\ clusters}} \cr {{c_{ij}}} & {{\rm otherwise}} \cr } } \right.$

Obviously, if the value of *M* is sufficiently large, then the best Hamiltonian cycle in *G′* is a feasible CTSP solution in *G*, in which the vertices of each cluster are visited contiguously.

**Property.***An optimal solution to the TSP instance corresponds to an optimal solution to the original CTSP instance*.

**Proof.** Let *S′* and *S* be the optimal solutions of the TSP instance *G′* and the original CTSP instance *G*, respectively. Let *m* be the number of clusters of *G*. To minimize the total travel cost, there are only *m* inter-cluster edges in *S′*. Therefore, *S′* is a feasible CTSP solution for *G* and satisfies the following relation:

$f({S^{'}}) = f(S) + m \times M$

Obviously, *S′* corresponds to *S* by subtracting the constant *m* × *M*.

### Solution methods for the TSP

There are numerous solution methods for the TSP. In this work, we adopt three very powerful TSP solvers whose codes are publicly available, including one exact solver (Concorde (Applegate, Bixby & Chvatal, 2006)) and two inexact (heuristic) solvers (LHK-2 (Helsgaun, 2009) and GA-EAX (Nagata & Kobayashi, 2013)).

Notice that the TSP instance converted from a CTSP instance has a particular feature that the vertices are grouped into clusters and the distance between each pair of vertices within a same cluster is in general small, while this distance is large for two vertices from different clusters. Along with the presentation of the TSP solvers, we discuss their suitability for solving such clustered instances each time this is appropriate.

#### Exact Concorde solver

Concorde is an advanced exact TSP solver for the symmetric TSP based on Branch-and-Bound and problem specific cutting plane methods (Applegate, Bixby & Chvatal, 2006). It makes use of a specifically designed QSopt linear programming solver. According to Hoos & Stützle (2014), Concorde is the best performing exact algorithm for the TSP. As shown in Applegate et al. (2006), Concorde can solve benchmark instances from TSPLIB with up to 1,000 vertices to optimality within a reasonable computation time and it also solves large TSP instances at the cost of a long computation time.

The run time behavior of Concorde has been investigated essentially on random uniform instances. For instance, Applegate et al. (2006) investigated the run time required by Concorde for solving random uniform instances and indicated that the run time increases as an exponential function of instance size |*V*|. Hoos & Stützle (2014) further demonstrated that the median run time required by Concorde scales with instance size |*V*| of the form $a{b^{\sqrt {|V|} }}$ (*a* ≈ 0.21, *b* ≈ 1.24) on the widely studied class of uniform random TSP instances. To our knowledge, no study has been reported concerning the behavior of Concorde on sharply clustered instances. As a result, the current study will provide useful information on this issue.

#### Lin-Kernighan based heuristic solver

According to the TSP literature, a majority of the best performing TSP heuristic algorithms is based on the Lin-Kernighan (LK) heuristic (Lin & Kernighan, 1973) and its extensions. The LK heuristic is a variable-depth *k*-opt local search procedure, where the *k*-opt neighborhood is partially searched with a smart pruning strategy. LK explores the most promising neighbors within the *k*-opt neighborhood, that is, the set of feasible tours obtained by removing *k* edges and adding other *k* edges such that the resulting tour is feasible. Several improved versions of the basic LK heuristic have been introduced within the iterated local search framework (*e.g*., Applegate, Cook & Rohe, 2003; Helsgaun, 2000; Helsgaun, 2009; Martin, Otto & Felten, 1991).

Among these iterated LK algorithms, Helsgaun’s LKH (Helsgaun, 2000, 2009) is the uncontested state-of-the-art heuristic TSP solver. Helsgaun (2000) developed an iterated version of LK together with an efficient implementation of the LK algorithm, known as the Lin-Kernighan-Helsgaun (LKH-1) heuristic, where a 5-opt move is used as the basic move to broaden the search and an *α*-measure method based on sensitivity analysis of minimum spanning trees is used to restrict the search to relative few of the *α*-nearest neighbors of a vertex to speed up the search process. Later, Helsgaun (2009) further extended LKH-1 by developing a highly effective implementation of the *k*-opt procedure (called LKH-2), which eliminated many of the limitations and shortcomings of LKH-1. Furthermore, LKH-2 specially extended the data structures of LKH-1 to solve very large TSP instances. The main features of LKH-2 include (1) using sequential and non-sequential *k*-opt moves, (2) using several partitioning procedures to partition a large TSP instance into smaller subproblems, (3) using a tour merging procedure to generate a better solution from two or more local optimum solutions, and (4) applying a backbone-guided search to guide the local search to make biased local perturbations. LKH-2 is considered to be one of most effective heuristic methods for finding very high-quality solutions for various large TSP instances (Dubois-Lacoste, Hoos & Stützle, 2015).

However, the LK algorithm and any LK-based algorithms require much longer running times on clustered instances of the TSP than on uniformly distributed instances (Neto, 1999). The main reason why the LK heuristic stumbles on clustered instances is that relatively large inter-cluster edges serve as bait edges. During the LK search, when removing such a bait edge, the LK heuristic is tricked into long and often fruitless searches. More precisely, each time an edge bridging two clusters is removed, the cumulative gain rises enormously, and the procedure is encouraged to perform very deep searches. To alleviate the problem, a cluster compensation technique was proposed in Neto (1999) for the Lin-Kernighan heuristic to limit its performance degradation. Helsgaun (2009) showed that the LKH-2 algorithm performs significantly worse on sharply clustered instances than on uniform random instances. To remedy this difficulty, Helsgaun (2014) considered the unusual structure of clustered instances, and adjusted the parameter settings of LKH-2 to better solve the clustered instances. The resulting solver is named CLKH, which is used in this study.

#### Edge assembly crossover based genetic algorithm

Population-based evolutionary algorithms are another well-known approach for the TSP. A popular example is the powerful genetic algorithm introduced by Nagata & Kobayashi (2013). This algorithm (called GA-EAX, see Algorithm 1) is characterized by its powerful edge assembly crossover (EAX) operator introduced in Nagata & Kobayashi (1997) and Nagata & Soler (2012) with an efficient implementation and a cost-effective selection strategy for maintaining population diversity.

**Algorithm 1:** GA-EAX for the CTSP.

**Require:** TSP instance *G*, population size *p*; number of offspring solutions *r* generated from each parent pair
**Ensure:** best solution S*
1: $POP = \{ {P_1},{P_2},...,{P_p}\} \leftarrow$ Initial_Population(*G*)
2: **while** stopping condition is not met **do**
3: Randomly shuffle the solutions in *POP*
4: **for***i* = 1,2,…, *p***do**
5: ${S_1} \leftarrow {P_i}$, ${S_2} \leftarrow {P_{i + 1}}$ /* Note: *P*_*p* + 1_ = *P*_1_ */
6: $({o_1},...,{o_r}) \leftarrow$ EAX(*S*_1_, *S*_2_)
7: ${P_i} \leftarrow$ Select_Best(*o*_1_,…, *o*_*r*_, *S*_1_)
8: **end for**
9**: end while**
10: ${S^*} \leftarrow$ Best(*POP*)
11: Return S*

**Figure 2:**
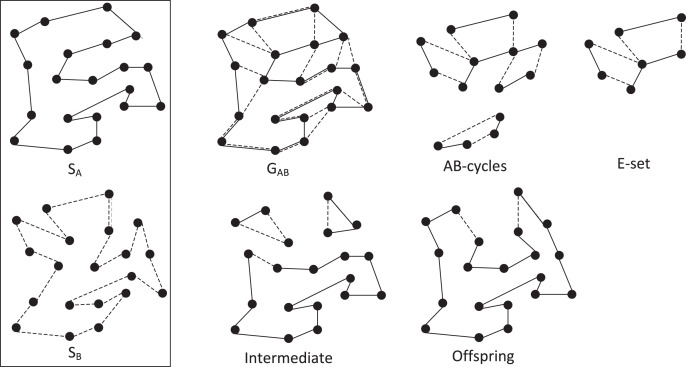
Illustrative example of the EAX crossover operator.

The key EAX operator generates, from two high-quality tours (parents), one offspring tour by first inheriting the edges from the parents to construct disjoint subtours and then connecting the subtours with new edges in a greedy fashion (similar to building a minimal spanning tree). Let *S*_*A*_ and *S*_*B*_ be the parents, EAX operates as follows (see Fig. 2 for an example):

1. Generate an undirected multigraph defined as *G*_*AB*_ = (*V*, *E*_*A*_ ∪ *E*_*B*_), where *E*_*A*_ and *E*_*B*_ are the sets of edges of parents *S*_*A*_ and *S*_*B*_, respectively.
2. Extract all AB-cycles from *G*_*AB*_. An AB-cycle is defined as a cycle in *G*_*AB*_, such that edges of *E*_*A*_ and edges of *E*_*B*_ are alternately linked.
3. Construct an E-set by selecting AB-cycles according to a given selection strategy (*e.g*., single, k-multiple, block and block2 (Nagata & Kobayashi, 2013)), where an E-set is a set of AB-cycles.
4. Copy parent *S*_*A*_ to an intermediate solution *o*. Then, remove the edges of *E*_*A*_ in the E-set from *o* and add those of *E*_*B*_ in the E-set to *o*. This leads to an intermediate solution *o* with one or more subtours.
5. Connect all the subtours in *o* with new short edges to generate a complete tour (a feasible offspring solution) by using a greedy heuristic.

Note that different versions of EAX can be developed by using different selection strategies of AB-cycles for constructing E-sets. The GA-EAX algorithm employs the single and block2 strategies to generates offspring solutions from parent solutions. To maintain a healthy population diversity, GA-EAX also uses an edge entropy measure to select the solution to be used to replace a parent in the population.

Other studies (*e.g*., Hains, Whitley & Howe, 2012) also indicated the usefulness of edge-assembly-like crossovers for solving clustered instances of the TSP. As shown in the next section, the EAX-based genetic algorithm performs remarkably well on all the clustered instances transformed from the CTSP.

## Computational experiments

In this section, we evaluate the capacity of the TSP solvers presented in “Solution Methods for the TSP” to solve the CTSP *via* its transformation to the TSP. For this purpose, we examine their qualitative performances and run time efficiencies on various benchmark instances and make comparisons with the best dedicated CTSP algorithms in the literature.

### Benchmark instances

Our computational assessments are based on three sets of 73 benchmark instances with 101 to 24,978 vertices. Sets 1 and 2 include 20 medium instances (101 ≤ |*V*| ≤ 1,000) and 15 large instances (1,173 ≤ |*V*| ≤ 2,000), which are classical and widely used in the CTSP literature (*e.g*., Mestria, Ochi & de Lima Martins, 2013; Mestria, 2016; Mestria, 2018). Set 3 includes 38 large GTSP instances (1,000 ≤ |*V*| ≤ 24,978) from Helsgaun (2014).

**Sets 1 and 2 (35 instances)**: These instances belong to the following six types: (1) instances taken from the TSPLIB (Reinelt, 1991) where the clusters are generated by using a k-means clustering algorithm; (2) instances created from a selection of classic TSP instances (Johnson & McGeoch, 2007), where the clusters are created by grouping the vertices in geometric centers; (3) instances generated by using the Concorde interface (Applegate, Bixby & Chvatal, 2006); (4) instances generated using the layout proposed in Laporte & Palekar (2002); (5) instances similar to type 2, but generated with different parameters; (6) instances adapted from the TSPLIB (Reinelt, 1991), where the rectangular floor plan is divided into several quadrilaterals and each quadrilateral corresponds to a cluster. These instances are available at http://www2.ic.uff.br/~labic/conteudo/instance/.

**Set 3 (38 instances)**: These large instances have 1,000 to 24,978 vertices and come from GTSPLIB for the generalized traveling salesman problem (GTSP). They were generated from TSP instances by using Fischetti, Salazar González & Toth (1997) clustering algorithm and tested in Helsgaun (2014) by considering them as CTSP instances. These instances are available at http://www.ruc.dk/~keld/research/CLKH. In Helsgaun (2014), six very large instances with 31,623 to 85,900 vertices were also tested. We ignore these instances, because they are too large for the exact Concorde solver and the GA-EAX solver stops abnormally when solving these instances.

### TSP solvers and experimental protocol

For our study, we employed three representative TSP solvers presented in “Solution Methods for the TSP”, which are among the most powerful methods for the TSP in the literature.

- Exact Concorde TSP solver (http://www.math.uwaterloo.ca/tsp/concorde/index.html): We used version Concorde-03.12.19 and ran the solver with its default parameter setting with a cutoff time of 24 CPU hours per instance.
- Inexact CLKH solver (http://www.ruc.dk/~keld/research/CLKH): We used the version CLKH-1.0 which is based on the latest version 2.0.9 (http://akira.ruc.dk/~keld/research/LKH/) of LKH-2. The default parameter setting given in Helsgaun (2014) was adopted to run CLKH. Notice that to reduce its run time, the maximum number of trials (iterations) is set to 1,000 in CLKH, while this number is set to *n* (instance size) by default in LKH-2.
- Inexact GA-EAX TSP solver (https://github.com/sugia/GA-for-TSP): We used GA-EAX with its default parameter setting given in Nagata & Kobayashi (2013): *p* = 300, *r* = 30 and GA-EAX terminates if the difference between the average tour length and the shortest tour length in the population is less than 0.001. Following Kerschke et al. (2018) and Kotthoff et al. (2015), we reset the random seed for GA-EAX for each run (which was set to a fixed value in the official implementation).

The experiments were carried out on a computer running Linux operating system with an Intel E5-2670 processor (2.8 GHz and 4G RAM). Given the stochastic nature of CLKH and GA-EAX, we ran each algorithm 10 times for each instances while the deterministic Concorde TSP solver was run one time to solve each instance.

### Computational results and comparison of popular TSP solvers

Our computational studies aim to answer the following questions: How do state-of-the-art *exact* TSP solvers perform on clustered instances converted from the CTSP? How do state-of-the-art *inexact* (heuristic) TSP solvers perform on clustered instances converted from the CTSP?

The results of the three TSP solvers (Concorde, CLKH, GA-EAX) on the 20 medium and 15 large CTSP benchmark instances are summarized in Table 1 (Set 1) and Table 2 (Set 2). Columns 1 to 3 show the basic information of each instance: the instance name (Instance), the number of vertices (|*V*|) and the number of clusters (*m*). Column 4 gives the optimal objective value reported by the exact Concorde TSP solver, followed by the required run time in seconds. For both the CLKH and GA-EAX solvers, we show the best (*Gap*_*best*_) and average (*Gap*_*avg*_) results over 10 independent runs in the form of the percentage gap to the optimal solution, as well as the average run time in seconds. If the best solution over 10 independent runs equals the optimal solution obtained with the exact Concorde TSP solver, the corresponding cell in column *Gap*_*best*_ shows ‘=’ along with the number of runs that succeeded in finding the optimal solution. Finally, row ‘Avg.’ provides the average run time in seconds for each approach, and the average gap between the average objective values obtained with CLKH/GA-EAX and the optimal values obtained with the Concorde TSP solver.

**Table 1:** Computational results of the TSP solvers Concorde, CLKH and GA-EAX on medium CTSP instances (Set 1).

			Concorde		CLKH			GA-EAX		
Instance	|*V*|	*m*	Opt.	*t*(*s*)	*Gap*_*best*_	*Gap*_*avg*_	*t*(*s*)	*Gap*_*best*_	*Gap*_*avg*_	*t*(*s*)
i-50-gil262	262	50	135,431	1.9	=(10)	0.0000	1.3	=(10)	0.0000	1.7
10-lin318	318	10	529,584	2.2	=(10)	0.0000	19.5	=(10)	0.0000	1.8
10-pcb442	442	10	537,419	20.7	=(10)	0.0000	46.9	=(10)	0.0000	6.3
C1k.0	1,000	10	132,521,027	21.9	=(9)	0.0001	128.6	=(10)	0.0000	16.3
C1k.1	1,000	10	129,128,125	22.3	=(10)	0.0000	70.6	=(10)	0.0000	14.3
C1k.2	1,000	10	142,784,000	69.9	0.0009	0.0009	244.6	=(9)	0.0001	17.2
300-6	300	6	8,934	4.4	=(10)	0.0000	30.2	=(10)	0.0000	3.5
400-6	400	6	9,045	6.7	=(10)	0.0000	26.7	=(10)	0.0000	4.4
700-20	700	20	41,425	29.9	=(10)	0.0000	200.0	=(10)	0.0000	10.2
200-4-h	200	4	62,777	0.6	=(10)	0.0000	5.4	=(10)	0.0000	0.9
200-4-x1	200	4	60,574	1.1	=(10)	0.0000	6.5	=(10)	0.0000	0.9
600-8-z	600	8	128,891	9.9	=(10)	0.0000	48.2	=(10)	0.0000	5.3
600-8-x2	600	8	128,891	4.8	=(10)	0.0000	48.2	=(10)	0.0000	5.3
300-5-108	300	5	67,760	1.2	=(10)	0.0000	8.5	=(10)	0.0000	2.0
300-20-111	300	20	309,739	1.8	=(10)	0.0000	6.0	=(10)	0.0000	2.0
500-15-306	500	15	194,818	2.6	=(10)	0.0000	37.1	=(10)	0.0000	5.2
500-25-308	500	25	365,447	4.4	=(10)	0.0000	10.1	=(10)	0.0000	5.4
25-eil101	101	25	23,671	0.5	=(10)	0.0000	0.4	=(10)	0.0000	0.8
42-a280	280	42	129,645	2.3	=(10)	0.0000	2.4	=(10)	0.0000	1.7
144-rat783	783	144	914,228	70.2	=(10)	0.0000	14.6	=(10)	0.0000	9.4
Avg.				14.0		0.0001	47.8		0.0000	5.7

**Table 2:** Computational results of the TSP solvers Concorde, CLKH and GA-EAX on large CTSP instances (Set 2).

			Concorde		CLKH			GA-EAX		
Instance	|*V*|	*m*	Opt.	*t*(*s*)	*Gap*_*best*_	*Gap*_*avg*_	*t*(*s*)	*Gap*_*best*_	*Gap*_*avg*_	*t*(*s*)
49-pcb1173	1,173	49	61,600	5,638.3	0.6250	1.0519	1,065.8	=(4)	0.0326	35.0
100-pcb1173	1,173	100	63,382	588.3	=(7)	0.0066	63.2	=(8)	0.0013	32.5
144-pcb1173	1,173	144	62,142	38.4	=(10)	0.0000	25.8	=(10)	0.0000	18.6
10-nrw1379	1,379	10	58,783	562.9	=(10)	0.0000	174.9	=(6)	0.0070	26.8
12-nrw1379	1,379	12	59,129	58.5	=(10)	0.0000	39.7	=(9)	0.0007	27.6
1500-10-503	1,500	10	11,116	65.5	=(5)	0.0225	603.6	=(10)	0.0000	28.4
1500-20-504	1,500	20	15,698	40.7	=(10)	0.0000	167.9	=(5)	0.0172	34.5
1500-50-505	1,500	50	22,900	67.0	=(7)	0.0476	178.8	=(5)	0.0044	35.1
1500-100-506	1,500	100	29,799	108.7	=(6)	0.0228	58.3	=(8)	0.0020	39.5
1500-150-507	1,500	150	34,068	114.7	=(10)	0.0000	44.4	=(10)	0.0000	32.3
2000-10-a	2,000	10	105,360	7214.3	0.0038	0.0155	401.9	0.0826	0.1167	45.3
2000-10-h	2,000	10	33,708	812.7	=(9)	0.0006	229.9	=(10)	0.0000	35.6
2000-10-z	2,000	10	33,509	200.9	=(10)	0.0000	160.1	=(9)	0.0003	37.3
2000-10-x1	2,000	10	33,792	1,325.4	=(4)	0.0213	485.3	=(6)	0.0136	35.6
2000-10-x2	2,000	10	33,509	170.9	=(10)	0.0000	160.1	=(10)	0.0000	39.6
Avg.				1,133.8		0.0793	257.3		0.0131	33.6

From Tables 1 to 2, we can make the following observations:

First, the exact Concorde TSP solver performs very well on these 35 instances and is able to solve all of them exactly. Specifically, the 20 medium instances can be solved easily in a short run time (an average of about 14 s). The 15 large instances are more difficult and the run time needed to solve these instances increases considerably (an average of 1,133.8 s, reaching 7,214.3 s for the most difficult instance).

Second, the CLKH solver performs globally very well on these 35 instances. For the 20 medium instances, CLKH attains all the optimal solutions but one with an average run time of 47.8. For the 15 large instances, CLKH reaches the optimal solutions for 13 instances with an average run time of 257.3 s.

Third, the GA-EAX solver performs remarkably well by attaining the optimal values for all 35 instances but one. For the 20 medium instances, GA-EAX consistently hits the optimal solutions for each of its 10 run (except for one instance for which it has a hit of 9 out of 10). The average run time is only 5.7 s for the medium instances and 33.6 s for the large instances. Compared to Concorde and CLKH, GA-EAX is thus extremely time efficient. Moreover, contrary to the Concorde and CLKH solvers, the computation time required by GA-EAX remains very stable across the instances of the same set, indicating a high robustness and scalability of this solver.

Table 3 presents the results of the three TSP solvers on the 38 large GTSP instances of Set 3. Notice that the Concorde solver failed to exactly solve 17 instances in 24 h, the corresponding cell (in parentheses) in column ‘Optimum’ indicates the best tour length (best upper bound) found by CLKH and GA-EAX. In this case, the percentage gaps (*Gap*_*best*_ and *Gap*_*avg*_) are calculated by using the best bound, and column *Gap*_*best*_ shows ‘=’ the number of runs for an algorithm to find the best bound.

**Table 3:** Computational results of the TSP solvers Concorde, CLKH and GA-EAX on large GTSP instances (Set 3).

					CLKH			GA-EAX		
Instance	|*V*|	*m*	Optimum	Concorde’s run-time	*Gap*_*best*_	*Gap*_*avg*_	*t*(*s*)	*Gap*_*best*_	*Gap*_*avg*_	*t*(*s*)
10C1k.0	1,000	10	12,139,627	23.5	=(9)	0.0016	194.5	=(10)	0.0000	16.1
200C1k.0	1,000	200	11,929,315	17.4	=(10)	0.0000	64.7	=(10)	0.0000	15.6
200E1k.0	1,000	200	24,468,822	66.2	=(8)	0.0008	27.7	=(10)	0.0000	15.1
49usa1097	1,097	49	77,583,052	51.1	=(7)	0.0069	128.6	=(10)	0.0000	23.7
235pcb1173	1,173	235	59,796	65.5	=(9)	0.0151	36.4	=(10)	0.0000	16.4
259d1291	1,291	259	55,962	8,402.5	0.0286	0.0484	51.7	=(7)	0.0064	17.3
261rl1304	1,304	261	261,132	19.2	=(10)	0.0000	18.7	=(10)	0.0000	7.5
265rl1323	1,323	265	280,004	3,361.3	0.0114	0.0381	18.9	=(8)	0.0019	10.2
276nrw1379	1,379	276	60,473	234.4	=(3)	0.0223	30.8	=(10)	0.0000	30.7
280fl1400	1,400	280	20,229	6,108.5	=(3)	0.0900	504.7	=(8)	0.0178	21.5
287u1432	1,432	287	162,151	23,029.9	=(8)	0.0136	111.6	=(10)	0.0000	26.8
316fl1577	1,577	316	23,023	1,179.6	=(10)	0.0000	183.0	=(2)	0.2332	17.2
331d1655	1,655	331	65,871	142.9	=(3)	0.0797	51.8	=(7)	0.0029	24.6
350vm1748	1,748	350	348,244	230.9	=(2)	0.0371	88.2	=(10)	0.0000	25.7
364u1817	1,817	364	61,879	5,675.7	=(1)	0.0739	77.7	=(6)	0.0050	31.8
378rl1889	1,889	378	323,040	461.5	=(1)	0.1197	29.0	=(10)	0.0000	18.1
421d2103	2,103	421	(91,637)	–	=(2)	0.0598	112.7	=(10)	0.0000	32.8
431u2152	2,152	431	(69,876)	–	=(2)	0.0215	98.5	=(10)	0.0000	37.0
464u2319	2,319	464	(246,707)	–	=(10)	0.0000	703.2	=(3)	0.0167	84.9
479pr2392	2,392	479	397,707	1,267.5	=(4)	0.0223	102.1	=(10)	0.0000	38.0
608pcb3038	3,038	608	146,351	45,008.4	0.0014	0.0256	115.5	=(4)	0.0018	83.2
31C3k.0	3,162	31	20,058,457	912.6	0.0144	0.0637	249.2	=(5)	0.0211	111.6
633C3k.0	3,162	633	20,158,425	1,650.4	0.0207	0.0869	163.5	=(8)	0.0011	98.0
633E3k.0	3,162	633	42,697,510	5,239.0	0.0036	0.0226	105.7	=(3)	0.0052	115.0
759fl3795	3,795	759	(29,582)	–	=(9)	0.0068	464.0	0.2637	0.3729	53.9
893fnl4461	4,461	893	(193,834)	–	=(2)	0.0163	139.0	=(8)	0.0004	236.6
1183rl5915	5,915	1,183	(599,096)	–	0.0212	0.1666	204.3	=(9)	0.0006	146.6
1187rl5934	5,934	1,187	(588,074)	–	0.0126	0.1256	251.6	=(5)	0.0033	156.1
1480pla7397	7,397	1,480	(23,926,551)	–	0.0035	0.0213	1104.7	=(2)	0.0078	388.2
100C10k.0	10,000	100	(36,352,580)	–	=(1)	0.6815	1877.4	0.0525	0.4872	2318.7
2000C10k.0	10,000	2,000	(34,574,383)	–	0.0369	0.2590	730.6	=(1)	0.0139	992.9
2000E10k.0	10,000	2,000	(75,506,665)	–	0.0112	0.0281	635.8	=(1)	0.0013	1,320.0
2370rl11849	11,849	2,370	(977,472)	–	0.0081	0.0477	757.1	=(1)	0.0028	1,051.9
2702usa13509	13,509	2,702	(20,836,160)	–	0.0118	0.0185	1028.6	=(1)	0.0012	2,154.0
2811brd14051	14,051	2,811	(496,827)	–	0.0125	0.0213	944.7	=(1)	0.0024	2,454.9
3023d15112	15,112	3,023	(1,658,091)	–	0.0220	0.0296	1193.3	=(1)	0.0019	3,864.0
3703d18512	18,512	3,703	(683,839)	–	0.0209	0.0328	1561.9	=(1)	0.0019	4,306.8
4996sw24978	24,978	4,996	(893,042)	–	0.0237	0.0369	2076.3	=(1)	0.0008	5,706.2
Avg.						0.0616	427.3		0.0319	686.0

From Table 3, we can make the following observations. First, Concorde manages to optimally solve 21 large GTSP instances with up to 3,162 vertices with a run time ranging from 17.4 s to 45,008.4 s while its solution time is not completely consistent with the size of the problem instances. For the 21 instances that can be solved exactly by Concorde, CLKH attains 15 best upper bounds, while GA-EAX reaches all best upper bounds in less computing time. Second, for most of the instances with |*V*| < 10,000, compared with CLKH, GA-EAX has a better performance both in terms of solution quality and computation time. For the instances with 10,000 ≤ |*V*| ≤ 24,978, the solution quality of GA-EAX is better than that of CLKH in most cases, while requiring more computation time.

To sum up, the exact Concorde solver is very efficient for the instances with up to 1,000 vertices (order of seconds) and can even find optimal solutions for instances with up to some 3,000 vertices at a price of more run time (order of minutes to hours). For larger instances, both inexact solvers (CLKH and GA-EAX) are reliable alternatives to find optimal or sub-optimal solutions with some advantages for GA-EAX. These heuristic solvers also perform very well on smaller instances.

To deepen our computational study, we call upon to the performance profile, an analytic tool for evaluating the performances of multiple compared optimization algorithms (Dolan & Moré, 2002). The performance profile uses a cumulative distribution function for a performance metric, such as run time, objective function values, number of iterations, and so on. Precisely, let *S* be a set of algorithms and *P* be a set of problem instances. For a given performance metric *f*_*s*,*p*_ (that is the performance of algorithm *s* ∈ *S* solving instance *p* ∈ *P*), the performance ratio is defined by ${r_{s,p}} = \displaystyle{{{f_{s,p}}} \over {min\{ {f_{a,p}}:a \in S\} }}$. Then, for each algorithm *s* ∈ *S*, the performance function is given by ${\rho _s}(\tau ) = \displaystyle{{|\{ p \in P:{r_{s,p}} \le \tau \} |} \over {|P|}}$. Thus, the value of *ρ*_*s*_(1) corresponds to the fraction of problem instances that algorithm *s* can achieve many times the performance of the best algorithm (meaning the probability that the algorithm *s* will win over the rest of the compared algorithms). For a large value *τ*, the value of *ρ*_*s*_(*τ*) indicates a high robustness of algorithm *s*.

**Figure 3:**
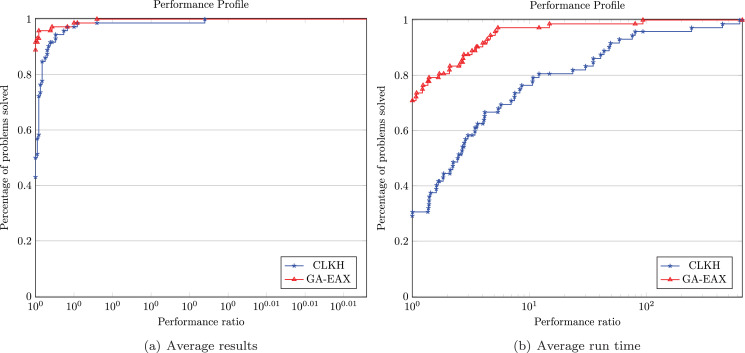
Performance profiles comparing solution quality and computing time.

To make a fair and meaningful comparison with this tool, we focus on the two inexact solvers CLKH and GA-EAX and run each solver 10 times on each of the 73 instances. We use the software ‘perprof-py’ (Siqueira, da Silva & Santos, 2016) to draw the performance profiles (see Fig. 3) where the quality of the solution is measured by the average objective value and average run time. These performance profiles tend to show an advantage of GA-EAX over CLKH for solving these clustered instances with up to 24,978 vertices.

### TSP solvers v.s. state-of-the-art CTSP heuristics

In “Computational Results and Comparison of Popular TSP Solvers”, we observed that the exact Concord TSP solver and the inexact CLKH and GA-EAX TSP solvers are powerful tools for solving clustered TSP instances converted from the CTSP. We now answer the following question: Do these general TSP solvers compete well with state-of-the-art CTSP heuristics specially designed for the problem?

For this purpose, we adopt GA-EAX as our representative TSP solver and compare it with three best performing CTSP heuristics in the literature: VNRDGILS (Mestria, 2018), HHGILS (Mestria, 2016), and GPR1R2 (Mestria, Ochi & de Lima Martins, 2013). Indeed, according to the experimental studies reported in Mestria, Ochi & de Lima Martins (2013) and Mestria (2016, 2018), these three heuristics perform the best among the recent CTSP heuristics available in the literature (see Table 4). This study is based on the 35 medium and large instances of Sets 1 and 2 (no results for the three CTSP heuristics are available on the large GTSP instances of Set 3).

**Table 4:** List of the reference algorithms for the CTSP.

Algorithm name	Reference	Search strategy
VNRDGILS	Mestria (2018)	A hybrid heuristic based on GRASP, ILS and VNRD
HHGILS	Mestria (2016)	A hybrid heuristic based on GRASP, ILS and VND
GPR1R2	Mestria, Ochi & de Lima Martins (2013)	A GRASP with Path Relinking PR1 and PR2
GPR1	Mestria, Ochi & de Lima Martins (2013)	A GRASP with Path Relinking PR1
GPR2	Mestria, Ochi & de Lima Martins (2013)	A GRASP with Path Relinking PR2
GPR3	Mestria, Ochi & de Lima Martins (2013)	A GRASP with Path Relinking PR3
GPR4	Mestria, Ochi & de Lima Martins (2013)	A GRASP with Path Relinking PR4
GRASP	Mestria, Ochi & de Lima Martins (2013)	A traditional GRASP heuristic
TLGA	Ding, Cheng & He (2007)	A two-level genetic algorithm

Table 5 provides the comparative results of the GA-EAX TSP solver along with the results reported by the three CTSP algorithms on the medium and large instances. For each instance and algorithm, columns ‘*f*_*best*_’, ‘*f*_*avg*_’ and ‘*t*(*s*)’ show respectively the best objective value over 10 independent runs, the average objective value and the average run time in seconds. Furthermore, the row ‘Avg.’ shows the average performances for each compared algorithm, including the average percentage gap of the best/average result to the optimal result obtained with the Concorde TSP solver and the average run time in seconds. To determine whether there exists a statistically significant difference in performance between the GA-EAX TSP solver and each CTSP algorithm in terms of best and average results, the *p*-values from the Wilcoxon signed-rank tests are given in the last row of Table 5. Entries with “-” mean that the corresponding results are not available in the literature. The best objective values obtained by the compared algorithms are indicated in bold if they attain the optimal solution. Notice that the results of the CTSP algorithms (VNRDGILS, HHGILS and GPR1R2) correspond to 10 executions per instance on a computer with 2.83 GHz Intel Core 2 CPU and 8 GB RAM and the time limit per run was set to 720 s for medium instances and 1,080 s for large instances.

**Table 5:** Comparative results between the GA-EAX TSP solver and three CTSP algorithms on medium and large CTSP instances. The best objective values obtained by the compared algorithms are indicated in bold if they attain the optimal solution.

			GA-EAX			VNRDGILS			HHGILS			GPR1R2		
Instance	|*V*|	*m*	*f*_*best*_	*f*_*avg*_	*t*(*s*)	*f*_*best*_	*f*_*avg*_	*t*(*s*)	*f*_*best*_	*f*_*avg*_	*t*(*s*)	*f*_*best*_	*f*_*avg*_	*t*(*s*)
i-50-gil262	262	50	**135,431**	135,431.0	1.7	135,483	135,510.2	720.0	135,510	135,578	720.0	–	–	–
10-lin318	318	10	**529,584**	529,584.0	1.8	530,604	530,871.4	720.0	530,283	530,817.9	720.0	530,443	532,697.9	720.0
10-pcb442	442	10	**537,419**	537,419.0	6.3	538,309	538,903.4	720.0	538,958	539,988.3	720.0	540,043	543,104.2	720.0
C1k.0	1,000	10	**132,521,027**	132,521,027.0	16.3	133,260,549	133,490,775.9	720.0	133,287,594	133,776,274.1	720.0	133,490,776	133,708,187.6	720.0
C1k.1	1,000	10	**129,128,125**	129,128,125.0	14.3	129,877,874	130,035,540.2	720.0	129,825,403	130,206,778.3	720.0	130,193,590	130,391,693.5	720.0
C1k.2	1,000	10	**142,784,000**	142,784,188.4	17.2	143,321,630	143,481,489.6	720.0	143,278,093	143,525,149.6	720.0	–	–	–
300-6	300	6	**8,934**	8,934.0	3.5	8,935	8,941.1	720.0	**8,934**	8,942.9	720.0	8959	8,985.3	720.0
400-6	400	6	**9,045**	9,045.0	4.4	9,053	9,062.3	720.0	9,051	9,063.2	720.0	–	–	–
700-20	700	20	**41,425**	41,425.0	10.2	41,456	41,489.7	720.0	41,452	41,485.6	720.0	41,540	41,573.3	720.0
200-4-h	200	4	**62,777**	62,777.0	0.9	62,867	63,058.3	720.0	62,804	63,058.3	720.0	62,994	63,710.2	720.0
200-4-x1	200	4	**60,574**	60,574.0	0.9	60,637	60,796.2	720.0	60,931	61,378.5	720.0	–	–	–
600-8-z	600	8	**128,891**	128,891.0	5.3	129,468	129,862.7	720.0	129,416	129,928.6	720.0	130,459	131,235.1	720.0
600-8-x2	600	8	**128,891**	128,891.0	5.3	129,246	129,533.9	720.0	129,246	129,691.5	720.0	–	–	–
300-5-108	300	5	**67,760**	67,760.0	2.0	67,766	67,868.7	720.0	67,814	67,930.5	720.0	–	–	–
300-20-111	300	20	**309,739**	309,739.0	2.0	310,146	310,270.9	720.0	310,209	310,427	720.0	309,928	310,551.9	720.0
500-15-306	500	15	**194,818**	194,818.0	5.2	194,946	195,201.5	720.0	195,202	195,438.1	720.0	–	–	–
500-25-308	500	25	**365,447**	365,447.0	5.4	365,717	365,937.8	720.0	365,828	366,085	720.0	366,232	366,785.7	720.0
25-eil101	101	25	**23,671**	23,671.0	0.8	23,673	23,685.2	720.0	23,678	23,690	720.0	23,676	23,711.3	720.0
42-a280	280	42	**129,645**	129,645.0	1.7	129,729	129,755.2	720.0	129,716	129,833.2	720.0	–	–	–
144-rat783	783	144	**914,228**	914,228.0	9.4	915,088	915,179.8	720.0	915,180	915,363.2	720.0	915,547	915,913.7	720.0
49-pcb1173	1,173	49	**61,600**	61,620.1	35.0	65,750	66,487.7	1,080.0	67,043	68,260.7	1,080.0	70,651	73,311.9	1,080.0
100-pcb1173	1,173	100	**63,382**	63,382.8	32.5	68,708	69,383.2	1,080.0	68,786	70,640.8	1,080.0	72,512	74,871.7	1,080.0
144-pcb1173	1,173	144	**62,142**	62,142.0	18.6	68,414	68,941.4	1,080.0	66,830	69,084.3	1,080.0	72,889	74,621.6	1,080.0
10-nrw1379	1,379	10	**58,783**	58,787.1	26.8	63,951	64,895.9	1,080.0	63,620	64,643.9	1,080.0	66,747	68,955.8	1,080.0
12-nrw1379	1,379	12	**59,129**	59,129.4	27.6	62,893	63,532.3	1,080.0	63,558	64,741.6	1,080.0	66,444	69,141.2	1,080.0
1500-10-503	1,500	10	**11,116**	11,116.0	28.4	11,969	12,103.0	1,080.0	11,986	12,109.5	1,080.0	12,278	12,531.4	1,080.0
1500-20-504	1,500	20	**15,698**	15,700.7	34.5	16,678	16,867.4	1,080.0	17,107	17,315.7	1,080.0	17,252	17,589.1	1,080.0
1500-50-505	1,500	50	**22,900**	22,901.0	35.1	24,631	24,803.6	1,080.0	25,264	25,558.9	1,080.0	25,124	25,761.5	1,080.0
1500-100-506	1,500	100	**29,799**	29,799.6	39.5	32,474	32,616.8	1,080.0	32,260	33,760.6	1,080.0	33,110	33,692.7	1,080.0
1500-150-507	1,500	150	**34,068**	34,068.0	32.3	37,357	38,251.1	1,080.0	37,658	38,433.1	1,080.0	38,767	39,478.0	1,080.0
2000-10-a	2,000	10	105,447	105,483.0	45.3	115,779	116,897.3	1,080.0	116,254	116,881.4	1,080.0	116,473	118,297.5	1,080.0
2000-10-h	2,000	10	**33,708**	33,708.0	35.6	36,806	38,351.8	1,080.0	36,447	37,305.1	1,080.0	37,529	38,861.8	1,080.0
2000-10-z	2,000	10	**33,509**	33,509.1	37.3	36,815	38,035.7	1,080.0	37,059	37,443.7	1,080.0	37,440	38,765.9	1,080.0
2000-10-x1	2,000	10	**33,792**	33,796.6	35.6	36,783	37,488.6	1,080.0	36,752	37,704.0	1,080.0	37,262	39,253.1	1,080.0
2000-10-x2	2,000	10	**33,509**	33,509.0	39.6	37,132	38,240.6	1,080.0	36,660	37,117.1	1,080.0	37,704	38,699.5	1,080.0
Avg.			0.00	0.01	17.7	3.79	4.62	874.3	3.94	4.96	874.3	6.98	8.94	920.0
*p*-value						2.477e−7	2.477e−7		3.651e−7	2.477e−7				

Table 6 summarizes the statistical results for each compared algorithm on the two sets of medium and large instances. The first row indicates the number of optimal solutions found by each approach. The average percentage gap of the best/average result from the optimal result is provided in row ‘Average *Gap*_*best*_/*Gap*_*avg*_’. Finally, row ‘Average time (s)’ provides the average run time in seconds for each algorithm.

**Table 6:** Statistical results for the GA-EAX TSP solver and three state-of-the-art CTSP algorithms on Set 1 (medium instances) and Set 2 (large instances). Dominating values are indicated in bold.

		GA-EAX	VNRDGILS	HHGILS	GPR1R2
Set 1	Optimal solutions	**20/20**	0/20	1/20	0/20
	Average *Gap*_*best*_/*Gap*_*avg*_ (%)	**0.00/0.00**	0.18/0.30	0.21/0.40	0.39/0.73
	Average time (s)	**5.7**	720.0	720.0	720.0
Set 2	Optimal solutions	**14/15**	0/15	0/15	0/15
	Average *Gap*_*best*_/*Gap*_*avg*_ (%)	**0.00/0.01**	8.61/10.39	8.92/11.04	12.25/15.51
	Average time (s)	**33.6**	1,080.0	1,080.0	1,080.0

From Tables 5 and 6, we observe that the GA-EAX solver significantly outperforms the three CTSP algorithms on the medium and large instances in terms of both the best and the average results. For the large instance set, the improvement gaps between the results of GA-EAX and those of the CTSP methods are very high, ranging from 10.39% to 15.49%. Furthermore, in terms of the average run time, GA-EAX is about 30 to 130 times faster than the CTSP algorithms. The above results thus indicate that the GA-EAX TSP solver has a strong dominance over current best performing CTSP approaches in the literature. In addition, the small *p*-values (<0.05) from the Wilcoxon signed-rank tests further confirm the statistically significant difference of the compared results.

**Figure 4:**
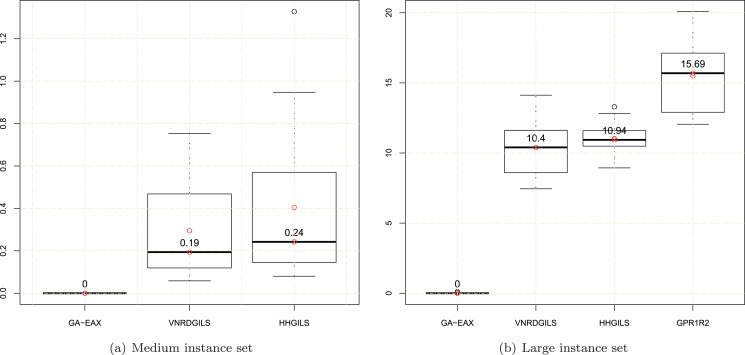
Boxplots of the normalized average objective values for the (A) medium instance set and (B) large instance set.

To have a finer analysis of the compared algorithms, Fig. 4 provides boxplot graphs to compare the distribution and range of the average results for each compared algorithm, except GPR1R2 for the medium instances since its results on several medium instances are not available. In this figure, the average objective value *f*_*avg*_ of a given algorithm is normalized according to the relation *y* = 100 * (*f*_*avg*_ − *f*_*opt*_)/*f*_*opt*_, where *f*_*opt*_ is the optimal value. The plots in Fig. 4 show clear differences in the distributions of the average results between GA-EAX and each compared CTSP heuristic, which further confirms the efficiency of the GA-EAX TSP solver with respect to these dedicated CTSP heuristics.

Finally, considering the results of the Concorde solver and the CLKH solver reported in “Computational Results and Comparison of Popular TSP Solvers”, we conclude that these TSP solvers also dominate the current best CTSP algorithms in the literature.

## Discussion

We now provide additional explanations regarding the behaviors of the three TSP solvers. First, given the NP-hard nature of the CTSP and the exponential time complexity of the exact Concorde solver, it is expected that the exact Concorde solver reaches its limit when the instance to be solved reaches some size (about 3,000 vertices for the studies instances). Indeed, when the search space becomes extremely large, the exact Branch-and-Bound search even equipped with the best problem specific cutting plane methods cannot effectively enumerate all candidate solutions. In fact, such a behavior has already been observed in previous studies on Concorde applied to classical TSP instances (Applegate et al., 2006; Hoos & Stützle, 2014). Second, regarding the two heuristic solvers CLKH and GA-EAX, the CLKH solver exhibits a worse performance compared to GA-EAX. As discussed in “Lin-Kernighan Based Heuristic Solver”, the underlying LK heuristic stumbles on clustered instances because relatively large intercluster edges serve as bait edges. With the presence of these bait edges, the LK heuristic may be tricked into long and often fruitless search trajectories. Third, the GA-EAX solver performs its search mainly with its edge assembly crossover, which inherits the edges of the parents to construct disjoint subtours and then connect the subtours. This crossover proves to be meaningful and helps the algorithm avoid local optimal traps. Once again, the excellent behavior of GA-EAX on the CTSP instances is consistent with its performance on conventional TSP instances as shown in Nagata & Kobayashi (2013).

## Conclusion

This work presents the first extensive computational study on the transformation approach of solving the Clustered Traveling Salesman Problem with general TSP solvers. Based on the results from the exact Concorde solver and the heuristic CLKH and GA-EAX solvers on 20 medium (101 ≤ |*V*| ≤ 1,000) and 15 large (1,173 ≤ |*V*| ≤ 2,000) CTSP benchmark instances and 38 large GTSP benchmark instances (with up to 24,978 vertices) available in the literature, we can draw the following conclusions.

- The exact Concorde solver can optimally solve all medium and large CTSP instances. It also solves exactly large GTSP instances with up to 3,162 vertices in a reasonable time, but fails to solve larger GTSP instances in 24 h. Its solution time is not completely consistent with the size of the problem instances.
- The heuristic CLKH and GA-EAX solvers perform very well both in terms of solution quality and computational efficiency. Both solvers have a good scalability, making them particularly suitable for solving very large instances with at least several thousands of vertices. For the tested instances with up to some 24,978 vertices, GA-EAX exhibits a better performance than CLKH.
- The general TSP solvers significantly dominate, both in terms of solution quality and computational efficiency, the current best performing CTSP heuristics specially designed for the problem. In particular, the TSP heuristics are several orders of faster than the state-of-the-art CTSP heuristics to find much better results.

This study indicates that the existing CTSP benchmark instances in the literature are not challenging for modern TSP solvers even if they remain difficult for the existing CTSP algorithms.

Finally, given the findings of this study, it would be interesting to investigate the problem transformation approach for solving other TSP variants that can be converted to the TSP or to a TSP variant for which effective algorithms are available.

## Supplemental Information

10.7717/peerj-cs.972/supp-1 Supplemental Information 1 Example code to add the artificial cost, M, to the edges of the internal clusters.

## Additional Information and Declarations

### Data Availability

The following information was supplied regarding data availability: All data and code are available at GitHub: https://github.com/lyldft/TSP.
