# Supplementary material for: Solving the clustered traveling salesman problem via traveling salesman problem methods
Source: PeerJ Comput Sci. 2022 Jun 13;8:e972. doi: 10.7717/peerj-cs.972 (PMC9202618; doi:10.7717/peerj-cs.972)
Supplement: Supplemental Information 1 [file peerj-cs-08-972-s001.tgz › CLKH-1.0/LKH-2.0.7/DOC/LKH-2_USER_GUIDE.pdf]

LKH
User Guide

Version 2.0.3 (July 2009)

by
Keld Helsgaun
E-mail: keld@ruc.dk

1. Introduction

The Lin-Kernighan heuristic [1] is generally considered to be one of the most successful methods for generating optimal or near-optimal solutions for the symmetric traveling salesman problem.

LKH is an implementation of a modified Lin-Kernighan heuristic. The new heuristic differs in many details from the original one. The most notable difference is found in the search strategy. The new heuristic uses larger (and more complex) search steps than the original one. Also new is the use of sensitivity analysis to direct and restrict the search. For a detailed description of the modified heuristic, see [2][3][4].

Computational experiments have shown that LKH is highly effective. Even though the algorithm is approximate, optimal solutions are produced with an impressively high frequency. LKH has produced optimal solutions for all solved problems we have been able to obtain, including an 85900-city problem (at the time of writing, the largest nontrivial problem solved to optimality).

2. Installation

LKH has been implemented in the programming language C. The software is entirely written in ANSI C and portable across a number of computer platforms and C compilers.

The code is distributed for research use. The author reserves all rights to the code.

The code is available at the Internet at:

http://www.akira.ruc.dk/~keld/research/LKH/

On a UNIX/LINUX machine execute the following commands:

```
tar xvfz LKH-2.0.3.tar
cd LKH-2.0.3
make
```

An executable file called LKH.UNIX will now be available in the directory LKH-2.0.3.

To verify that the software has been installed correctly, run the program by typing

```
./LKH pr2392.par
```

Then press return. The program should now solve a problem with 2392 cities.

The output should be similar to the following (produced by a 2.33 GHz Mac PowerBook):

```
PARAMETER_FILE = pr2392.par
Reading PROBLEM_FILE: "pr2392.tsp" ... done
ASCENT_CANDIDATES = 50
BACKBONE_TRIALS = 0
BACKTRACKING = NO
# CANDIDATE_FILE =
CANDIDATE_SET_TYPE = ALPHA
EXCESS = 0.00041806
EXTRA_CANDIDATES = 0
EXTRA_CANDIDATE_SET_TYPE = QUADRANT
GAIN23 = YES
GAIN_CRITERION = YES
INITIAL_PERIOD = 1196
INITIAL_STEP_SIZE = 1
INITIAL_TOUR_ALGORITHM = WALK
# INITIAL_TOUR_FILE =
INITIAL_TOUR_FRACTION = 1.000
# INPUT_TOUR_FILE =
KICK_TYPE = 0
KICKS = 1
# MAX_BREADTH =
MAX_CANDIDATES = 5
MAX_SWAPS = 2392
MAX_TRIALS = 2392
# MERGE_TOUR_FILE =
MOVE_TYPE = 5
NONSEQUENTIAL_MOVE_TYPE = 9
OPTIMUM = 378032
# OUTPUT_TOUR_FILE =
PATCHING_A = 2
PATCHING_C = 3
# PI_FILE =
PRECISION = 100
PROBLEM_FILE = pr2392.tsp
RESTRICTED_SEARCH = YES
RUNS = 10
SEED = 1
STOP_AT_OPTIMUM = YES
SUBGRADIENT = YES
# SUBPROBLEM_SIZE =
# SUBPROBLEM_TOUR_FILE =
SUBSEQUENT_MOVE_TYPE = 5
SUBSEQUENT_PATCHING = YES
# TIME_LIMIT =
# TOUR_FILE =
TRACE_LEVEL = 1
```

```
Lower bound = 373488.5, Gap = 1.20%, Ascent time = 8.8 sec.
Cand.min = 2, Cand.avg = 5.0, Cand.max = 5Preprocessing time = 7.3
sec.
* 1: Cost = 378224, Gap = 0.051%, Time = 0.5 sec.
* 2: Cost = 378032, Gap = 0.000%, Time = 0.6 sec. =
Run 1: Cost = 378032, Gap = 0.000%, Time = 0.6 sec. =

* 1: Cost = 378032, Gap = 0.000%, Time = 0.4 sec. =
Run 2: Cost = 378032, Gap = 0.000%, Time = 0.4 sec. =

* 1: Cost = 378032, Gap = 0.000%, Time = 0.5 sec. =
Run 3: Cost = 378032, Gap = 0.000%, Time = 0.5 sec. =

* 1: Cost = 378811, Gap = 0.206%, Time = 0.7 sec.
* 3: Cost = 378798, Gap = 0.203%, Time = 0.9 sec.
* 6: Cost = 378653, Gap = 0.164%, Time = 1.2 sec.
* 10: Cost = 378033, Gap = 0.000%, Time = 1.4 sec.
* 11: Cost = 378032, Gap = 0.000%, Time = 1.5 sec. =
Run 4: Cost = 378032, Gap = 0.000%, Time = 1.5 sec. =

* 1: Cost = 378032, Gap = 0.000%, Time = 0.6 sec. =
Run 5: Cost = 378032, Gap = 0.000%, Time = 0.6 sec. =

* 1: Cost = 378136, Gap = 0.028%, Time = 0.4 sec.
* 2: Cost = 378032, Gap = 0.000%, Time = 0.6 sec. =
Run 6: Cost = 378032, Gap = 0.000%, Time = 0.6 sec. =

* 1: Cost = 378370, Gap = 0.089%, Time = 0.4 sec.
* 4: Cost = 378075, Gap = 0.011%, Time = 1.2 sec.
* 6: Cost = 378053, Gap = 0.006%, Time = 1.4 sec.
* 29: Cost = 378032, Gap = 0.000%, Time = 1.9 sec. =
Run 7: Cost = 378032, Gap = 0.000%, Time = 1.9 sec. =

* 1: Cost = 378683, Gap = 0.172%, Time = 0.8 sec.
* 2: Cost = 378032, Gap = 0.000%, Time = 1.1 sec. =
Run 8: Cost = 378032, Gap = 0.000%, Time = 1.1 sec. =

* 1: Cost = 378787, Gap = 0.200%, Time = 0.7 sec.
* 3: Cost = 378634, Gap = 0.159%, Time = 0.9 sec.
* 4: Cost = 378615, Gap = 0.154%, Time = 1.1 sec.
* 8: Cost = 378032, Gap = 0.000%, Time = 1.7 sec. =
Run 9: Cost = 378032, Gap = 0.000%, Time = 1.7 sec. =

* 1: Cost = 378032, Gap = 0.000%, Time = 0.5 sec. =
Run 10: Cost = 378032, Gap = 0.000%, Time = 0.5 sec. =

Successes/Runs = 10/10
Cost.min = 378032, Cost.avg = 378032.00, Cost.max = 378032
Gap.min = 0.000%, Gap.avg = 0.000%, Gap.max = 0.000%
Trials.min = 1, Trials.avg = 5.8, Trials.max = 29
Time.min = 0.4 sec., Time.avg = 0.9 sec., Time.max = 1.9 sec.
```

A two-level tree [6] is used as the default tour representation. A three-level tree representation may be used in stead by compiling the source code with the compiler option

```
-DTHREE_LEVEL_TREE
```

Just edit the first line in `SRC/Makefile` and execute the commands

```
make clean
make
```

3. User interface

The software includes code both for reading problem instances and for printing solutions.

Input is given in two separate files:

(1) the *problem file* and
(2) the *parameter file*.

The *problem file* contains a specification of the problem instance to be solved. The file format is the same as used in TSPLIB [5], a publicly available library of sample instances for the TSP The library may be downloaded from

http://www.iwr.uni-heidelberg.de/iwr/comopt/soft/TSPLIB95/TSPLIB

The current version of the software allows specification of symmetric, asymmetric, as well as Hamiltonian tour problems.

Distances (costs, weights) may be given either explicitly in matrix form (in a full or triangular matrix), or implicitly by associating a 2- or 3-dimensional coordinate with each node. In the latter case distances may be computed by either a Euclidean, Manhattan, maximum, geographical or pseudo-Euclidean distance function. See the `TSPLIB_DOC.pdf` file for details. At present, all distances must be integral.

Problems may be specified on a complete or sparse graph, and there is an option to require that certain edges must appear in the solution of the problem.

The *parameter file* contains control parameters for the solution process. The solution process is typically carried out using default values for the parameters. The default values have proven to be adequate in many applications. Actually, almost all computational tests reported in this paper have been made using these default settings. The only information that cannot be left out is the name of the problem file.

The format is as follows:

PROBLEM_FILE = <*string*>
Specifies the name of the problem file.

Additional control information may be supplied in the following format:

ASCENT_CANDIDATES = <*integer*>
The number of candidate edges to be associated with each node during the ascent. The candidate set is complemented such that every candidate edge is associated with both its two end nodes.
Default: 50.

BACKBONE_TRIALS = <*integer*>
The number of backbone trials in each run.
Default: 0.

BACKTRACKING = { YES | NO }
Specifies whether a backtracking *K*-opt move is to be used as the first move in a sequence of moves (where K = MOVE_TYPE).
Default: NO.

CANDIDATE_FILE = <string>
Specifies the name of a file to which the candidate sets are to be written. If, however, the file already exists, the candidate edges are read from the file. The first line of the file contains the dimension of the instance. Each of the following lines contains a node number, the number of the dad of the node in the minimum spanning tree (0, if the node has no dad), the number of candidate edges emanating from the node, followed by the candidate edges. For each candidate edge its end node number and alpha-value are given. It is possible to give more than one CANDIDATE_FILE specification. In this case the given files are read and the union of their candidate edges is used as candidate sets.

CANDIDATE_SET_TYPE = { ALPHA | DELAUNAY [PURE] |
NEAREST-NEIGHBOR | QUADRANT }
Specifies the candidate set type. ALPHA is LKH's default type. It is applicable in general. The other three types can only be used for instances given by coordinates. The optional suffix PURE for the DELAUNAY type specifies that only edges of the Delaunay graph are used as candidates.
Default: ALPHA.

COMMENT <*string*>
A comment.
<*string*>
A comment.

EOF
Terminates the input data. The entry is optional.

EXCESS = <*real*>
The maximum alpha-value allowed for any candidate edge is set to EXCESS times the absolute value of the lower bound of a solution tour (determined by the ascent).
Default: value of 1.0/DIMENSION.

EXTRA_CANDIDATES = <*integer*> [SYMMETRIC]
Number of extra candidate edges to be added to the candidate set of each node. Their candidate set type may be specified using the keyword EXTRA_CANDIDATE_SET_ TYPE. The integer may be followed by the keyword SYMMETRIC, signifying that these extra candidate edges is to be complemented such that each of them is associated with both its two end nodes.
Default: 0.

EXTRA_CANDIDATE_SET_TYPE = { NEAREST-NEIGHBOR | QUADRANT }
The candidate set type of extra candidate edges.
Default: QUADRANT.

GAIN23 = [YES | NO]
Specifies whether the Gain23 function is used,
Default: YES.

GAIN_CRITERION ={ YES | NO }
Specifies whether Lin and Kernighan's gain criterion is used.
Default: YES.

INITIAL_PERIOD = <*integer*>
The length of the first period in the ascent.
Default: value of DIMENSION/2 (but at least 100).

INITIAL_STEP_SIZE = <*integer*>
The initial step size used in the ascent.
Default: 1.

INITIAL_TOUR_ALGORITHM ={ BORUVKA | GREEDY | MOORE |
NEAREST-NEIGHBOR | QUICK-BORUVKA |
SIERPINSKI | WALK }
Specifies the algorithm for obtaining an initial tour.
Default: WALK.

INITIAL_TOUR_FILE = <*string*>
Specifies the name of a file containing a tour to be used as the initial tour in the search. The tour is given by a list of integers giving the sequence in which the nodes are visited in the tour. The tour is terminated by a -1.

INITIAL_TOUR_FRACTION = <*real in [0;1]*>
Specifies the fraction of the initial tour to be constructed by means of INITIAL_ TOUR_FILE edges.
Default: 1.0.

INPUT_TOUR_FILE = <*string*>
Specifies the name of a file containing a tour. The tour is used to limit the search (the last edge to be excluded in a non-gainful move must not belong to the tour). In addition, the Alpha field of its edges is set to zero. The tour is given by a list of integers giving the sequence in which the nodes are visited in the tour. The tour is terminated by a -1.

KICK_TYPE = <*integer*>
Specifies the value of *K* for a random *K*-swap kick (an extension of the double-bridge move). If KICK_TYPE is zero, then the LKH's special kicking strategy, WALK, is used.
Default: 0

KICKS = <*integer*>
Specifies the number of times to "kick" a tour found by Lin-Kernighan. Each kick is a random *K*-swap kick. However, if KICKS is zero, then LKH's special kicking strategy, WALK, is used in stead.
Default: 1.

MAX_BREADTH = <*integer*>
Specifies the maximum number of candidate edges considered at each level of the search for a move.
Default: value of INT_MAX

MAX_CANDIDATES = <*integer*> { SYMMETRIC }
The maximum number of candidate edges to be associated with each node. The integer may be followed by the keyword SYMMETRIC, signifying that the candidate set is to be complemented such that every candidate edge is associated with both its two end nodes. If MAX_CANDIDATES is zero the candidate sets are made up of the edges represented in the CANDIDATE_FILEs, the INITIAL_TOUR_FILE, the INPUT_TOUR_FILE, the SUBPROBLEM_TOUR_FILE, and the MERGE_TOUR_FILEs.
Default: 5.

MAX_SWAPS = <integer>
Specifies the maximum number of swaps (flips) allowed in any search for a tour improvement.
Default: value of DIMENSION.

MAX_TRIALS = <*integer*>
The maximum number of trials in each run.
Default: number of nodes (DIMENSION, given in the problem file).

MERGE_TOUR_FILE = <*string*>
Specifies the name of a tour to be merged. The edges of the tour are added to the candidate sets. It is possible to give more than two MERGE_TOUR_FILE specifications.

MOVE_TYPE = <*integer*>
Specifies the sequential move type to be used in local search. A value $K >= 2$ signifies that a sequential K-opt move is to be used.
Default: 5.

NONSEQUENTIAL_MOVE_TYPE = <*integer*>
Specifies the nonsequential move type to be used. A value K >= 4 signifies that attempts are made to improve a tour by nonsequential k-opt moves where $4 <= k <= K$. Note, however, that the effect depends on the specifications of PATCHING_A and PATCHING_B.
Default: value of (MOVE_TYPE + PATCHING_A + PATCHING_B - 1).

OUTPUT_TOUR_FILE = <string>
Specifies the name of a file where the best tour is to be written. Each time a trial has produced a new best tour, the tour is written to this file. The character '$' in the name has a special meaning. All occurrences are replaced by the cost of the tour.

OPTIMUM = <*real*>
Known optimal tour length. If STOP_AT_OPTIMUM is YES, a run will be terminated if the tour length becomes equal to this value.
Default: value of -LLONG_MIN

PATCHING_A = <*integer*> [RESTRICTED | EXTENDED]
The maximum number of disjoint alternating cycles to be used for patching. An attempt to patch cycles is made if the corresponding non-sequential move is gainful. The integer may be followed by the keyword RESTRICTED or EXTENDED. The keyword RESTRICTED signifies that gainful moves are only considered if all its inclusion edges are candidate edges. The keyword EXTENDED signifies that the non-sequential move need not be gainful if only all its inclusion edges are candidate edges.
Default: 1.

PATCHING_C = <*integer*> [RESTRICTED | EXTENDED]
The maximum number of disjoint cycles to be patched in an attempt to find a feasible and gainful move. An attempt to patch cycles is made if the corresponding non-sequential move is gainful. The integer may be followed by the keyword RESTRICTED or EXTENDED. The keyword RESTRICTED signifies that gainful moves are only considered if all its inclusion edges are candidate edges. The keyword EXTENDED signifies that the non-sequential move need not be gainful if only all its inclusion edges are candidate edges.
Default: 0.

PI_FILE = <*string*>
Specifies the name of a file to which penalties (Pi-values determined by the ascent) are to be written. If the file already exists, the penalties are read from the file, and the ascent is skipped. The first line of the file contains the number of nodes. Each of the following lines is of the form

<*integer*> <*integer*>

where the first integer is a node number, and the second integer is the Pi-value associated with the node. The file name "0" represents a file with all Pi-values equal to zero.

POPULATION_SIZE=<*integer*>
Specifies the maximum size of the population in LKH's genetic algorithm. Tours found by the first POPULATION_SIZE runs constitute an initial population of tours. In each of the remaining runs two tours (parents) from the current population is recombined into a new tour (child) using a variant of the Edge Recombination Crossover (ERX). The parents are chosen with random linear bias towards the best members of the population. The child is used as initial tour for the next run. If this run produces a tour better than the worst tour of the population, then the resulting tour replaces the worst tour. Premature convergence is avoided by requiring that all tours in the population have different costs.
Default: 0.

PRECISION = <*integer*>
The internal precision in the representation of transformed distances:
d[i][j] = PRECISION*c[i][j] + pi[i] + pi[j],
where d[i][j], c[i][j], pi[i] and pi[j] are all integral.
Default: 100 (which corresponds to 2 decimal places).

RESTRICTED_SEARCH = [YES | NO]
Specifies whether the following search pruning technique is used: The first edge to be broken in a move must not belong to the currently best solution tour. When no solution tour is known, it must not belong to the minimum spanning 1-tree.
Default: YES.

RUNS = <*integer*>
The total number of runs.
Default: 10.

SEED = <*integer*>
Specifies the initial seed for random number generation.
Default: 1.

STOP_AT_OPTIMUM = [YES | NO]
Specifies whether a run is stopped, if the tour length becomes equal to OPTIMUM.
Default: YES.

SUBGRADIENT = [YES | NO]
Specifies whether the pi-values should be determined by subgradient optimization.
Default: YES.

SUBPROBLEM_SIZE = <*integer*> [DELAUNAY | KARP | K-MEANS | MOORE | SIERPINSKI]
[BORDERS] [COMPRESSED]
The number of nodes in a division of the original problem into subproblems. The division is made according to the tour given by SUBPROBLEM_TOUR_FILE. The value 0 signifies that no division is made. By default the subproblems are determined by subdividing the tour into segments of equal size. However, the integer may be followed by DELAUNAY, KARP, K-MEANS, ROHE, or SIERPINSKI. DELAUNAY signifies that a Delaunay partitioning scheme is used, KARP that Karp's partitioning scheme is used, K-MEANS that a partitioning scheme based on K-means clustering is used, ROHE that André Rohes's random rectangle partitioning scheme is used, and MOORE or SIERPINSKI that a partitioning scheme based on either a Moore or a Sierpinski space-filling curve is used. The BORDERS specification signifies that the subproblems along the borders between subproblems are to be solved too. The COMPRESSED specification signifies that each subproblem is compressed by removing from the problem all nodes with two incident subproblem tour edges that belong to all tours to be merged (at least two MERGE_TOUR_FILEs should be given).
Default: 0.

SUBPROBLEM_TOUR_FILE = <*string*>
Specifies the name of a file containing a tour to be used for dividing the original problem into subproblems. The approximate number of nodes in each is given by SUBPROBLEM_SIZE. The tour is given by a list of integers giving the sequence in which the nodes are visited in the tour. The tour is terminated by a -1

SUBSEQUENT_MOVE_TYPE = <*integer*>
Specifies the move type to be used for all moves following the first move in a sequence of moves. The value $K >= 2$ signifies that a K-opt move is to be used. The value 0 signifies that all moves are of the same type (K = MOVE_TYPE).
Default: 0.

SUBSEQUENT_PATCHING = { YES | NO }
Specifies whether patching is used for moves following the first move in a sequence of moves.
Default: YES.

TIME_LIMIT = <*real*>
Specifies a time limit in seconds for each run.
Default: value of DBL_MAX.

TOUR_FILE = <*string*>
Specifies the name of a file to which the best tour is to be written.

TRACE_LEVEL = <*integer*>
Specifies the level of detail of the output given during the solution process. The value 0 signifies a minimum amount of output. The higher the value is the more information is given.
Default: 1.

During the solution process information about the progress being made is written to standard output. The user may control the level of detail of this information (by the value of the TRACE_LEVEL parameter).

Before the program terminates, a summary of key statistics is written to standard output, and, if specified by the TOUR_FILE parameter, the best tour found is written to a file (in TSPLIB format).

The user interface is somewhat primitive, but it is convenient for many applications. It is simple and requires no programming in C by the user. However, the current implementation is modular, and an alternative user interface may be implemented by rewriting a few modules. A new user interface might, for example, enable graphical animation of the solution process.

References

[1] S. Lin & B. W. Kernighan,
"An Effective Heuristic Algorithm for the Traveling-Salesman Problem".
Oper. Res. **21**, 498-516 (1973).

[2] K. Helsgaun,
"An Effective Implementation of the Lin-Kernighan Traveling Salesman Heuristic".
Datalogiske Skrifter **81**, Roskilde University (1998).

[3] K. Helsgaun,
"An Effective Implementation of the Lin-Kernighan Traveling Salesman Heuristic".
European Journal of Operational Research **126** (1), 106-130 (2000).

[4] K. Helsgaun,
"An Effective Implementation of *K*-opt Moves for the Lin-Kernighan TSP Heuristic".
Datalogiske Skrifter **109**, Roskilde University (2006).

[5] G. Reinelt,
"TSPLIB - A Traveling Salesman Problem Library",
ORSA J. Comput. **3-4**, 376-385 (1991).

[6] M. L. Fredman, D. S. Johnson, L. A. McGeoch, and G. Ostheimer,
"Data Structures for Traveling Salesmen".
J. Algorithms, **18**(3) pp. 432-479 (1995).
