# Supplementary material for: Solving the clustered traveling salesman problem via traveling salesman problem methods
Source: PeerJ Comput Sci. 2022 Jun 13;8:e972. doi: 10.7717/peerj-cs.972 (PMC9202618; doi:10.7717/peerj-cs.972)
Supplement: Supplemental Information 1 [file peerj-cs-08-972-s001.tgz › CLKH-1.0/LKH-2.0.7/DOC/LKH_REPORT.pdf]

An Effective Implementation of the Lin-Kernighan Traveling Salesman Heuristic

Keld Helsgaun
E-mail: keld@ruc.dk

Department of Computer Science
Roskilde University
DK-4000 Roskilde, Denmark

Abstract

This report describes an implementation of the Lin-Kernighan heuristic, one of the most successful methods for generating optimal or near-optimal solutions for the symmetric traveling salesman problem. Computational tests show that the implementation is highly effective. It has found optimal solutions for all solved problem instances we have been able to obtain, including a 7397-city problem (the largest nontrivial problem instance solved to optimality today). Furthermore, the algorithm has improved the best known solutions for a series of large-scale problems with unknown optima, among these an 85900-city problem.

1. Introduction

The Lin-Kernighan heuristic [1] is generally considered to be one of the most effective methods for generating optimal or near-optimal solutions for the symmetric traveling salesman problem. However, the design and implementation of an algorithm based on this heuristic is not trivial. There are many design and implementation decisions to be made, and most decisions have a great influence on performance.

This report describes the implementation of a new modified version of the Lin-Kernighan algorithm. Computational experiments have shown that the implementation is highly effective.

The new algorithm differs in many details from the original one. The most notable difference is found in the search strategy. The new algorithm uses larger (and more complex) search steps than the original one. Also new is the use of sensitivity analysis to direct and restrict the search.

Run times of both algorithms increase approximately as $n^{2.2}$. However, the new algorithm is much more effective. The new algorithm makes it possible to find *optimal* solutions to large-scale problems, in reasonable running times.

For a typical 100-city problem the optimal solution is found in less than a second, and for a typical 1000-city problem optimum is found in less than a minute (on a 300 MHz G3 Power Macintosh).

Even though the algorithm is approximate, optimal solutions are produced with an impressively high frequency. It has produced optimal solutions for all solved problems we have been able to obtain, including a 7397-city problem (at the time of writing, the largest nontrivial problem solved to optimality).

The rest of this report is organized as follows. Section 2 defines the traveling salesman problem and gives an overview of solution algorithms. Section 3 describes the original algorithm of Lin and Kernighan (including their own refinements of the algorithm). Sections 4 and 5 present the new modified algorithm and its implementation. The effectiveness of the implementation is reported in Section 6.

2. The traveling salesman problem

2.1 Formulation

A salesman is required to visit each of n given cities once and only once, starting from any city and returning to the original place of departure. What tour should he choose in order to minimize his total travel distance?

The distances between any pair of cities are assumed to be known by the salesman. Distance can be replaced by another notion, such as time or money. In the following the term 'cost' is used to represent any such notion.

This problem, the traveling salesman problem (TSP), is one of the most widely studied problems in combinatorial optimization [2]. The problem is easy to state, but hard to solve. Mathematically, the problem may be stated as follows:

> Given a 'cost matrix' $C = (c_{ij})$, where c_{ij} represents the cost of going from city i to city j, (i, j = 1, ..., n), find a permutation $(i_1, i_2, i_3, \ldots, i_n)$ of the integers from 1 through n that minimizes the quantity
>
> $$c_{i_1 i_2} + c_{i_2 i_3} + \cdots + c_{i_n i_1}$$

Properties of the cost matrix C are used to classify problems.

- If $c_{ij} = c_{ji}$ for all i and j, the problem is said to be *symmetric*; otherwise, it is *asymmetric*.
- If the triangle inequality holds ($c_{ik} \le c_{ij} + c_{jk}$ for all i, j and k), the problem is said to be *metric*.
- If c_{ij} are Euclidean distances between points in the plane, the problem is said to be *Euclidean*. A Euclidean problem is, of course, both symmetric and metric.

2.2 Motivation

The importance of the TSP stems not from a massive need from salesmen wishing to minimize their travel distance. The importance comes from a wealth of other applications, many of which seemingly have nothing to do with traveling routes.

For example, consider the following process planning problem. A number of jobs have to be processed on a single machine. The machine can only process one job at a time. Before a job can be processed the machine must be prepared (cleaned, adjusted, or whatever). Given the processing time of each job and the switch-over time between each pair of jobs, the task is to find an execution sequence of the jobs making the total processing time as short as possible.

It is easy to see that this problem is an instance of TSP. Here c_{ij} represents the time to complete job j after job i (switch-over time plus time to perform job j). A pseudo job with processing time 0 marks the beginning and ending state for the machine.

Many real-world problems can be formulated as instances of the TSP. Its versatility is illustrated in the following examples of application areas:

- Computer wiring
- Vehicle routing
- Crystallography
- Robot control
- Drilling of printed circuit boards
- Chronological sequencing.

TSP is a typical problem of its genre: *combinatorial optimization*. This means that theoretical and practical insight achieved in the study of TSP can often be useful in the solution of other problems in this area. In fact, much progress in combinatorial optimization can be traced back to research on TSP. The now well-known computing method, *branch and bound*, was first used in the context of TSP [3, 4]. It is also worth mentioning that research on TSP was an important driving force in the development of the *computational complexity theory* in the beginning of the 1970s [5].

However, the interest in TSP not only stems from its practical and theoretical importance. The intellectual challenge of solving the problem also plays a role. Despite its simple formulation, TSP is hard to solve. The difficulty becomes apparent when one considers the number of possible tours - an astronomical figure even for a relatively small number of cities. For a symmetric problem with n cities there are (n-1)!/2 possible tours. If n is 20, there are more than 10^{18} tours. The 7397-city problem, which is successfully solved by the algorithm described in this report, contains more than $10^{25,000}$ possible tours. In comparison it may be noted that the number of elementary particles in the universe has been estimated to be ‘only’ 10^{87}.

2.3 Solution algorithms

It has been proven that TSP is a member of the set of NP-complete problems. This is a class of difficult problems whose time complexity is probably exponential. The members of the class are related so that if a polynomial time were found for one problem, polynomial time algorithms would exist for all of them. However, it is commonly believed that no such polynomial algorithm exists. Therefore, any attempt to construct a general algorithm for finding optimal solutions for the TSP in polynomial time must (probably) fail.

That is, for any such algorithm it is possible to construct problem instances for which the execution time grows at least exponentially with the size of the input. Note, however, that time complexity here refers to any algorithm's behavior in *worst* cases. It can not be excluded that there exist algorithms whose *average* running time is polynomial. The existence of such algorithms is still an open question.

Algorithms for solving the TSP may be divided into two classes:

- *Exact* algorithms;
- *Approximate* (or *heuristic*) algorithms.

2.3.1 Exact algorithms

The *exact* algorithms are guaranteed to find the optimal solution in a bounded number of steps. Today one can find exact solutions to symmetric problems with a few hundred cities, although there have been reports on the solution of problems with thousands of cities.

The most effective exact algorithms are cutting-plane or facet-finding algorithms [6, 7, 8]. These algorithms are quite complex, with codes on the order of 10,000 lines. In addition, the algorithms are very demanding of computer power. For example, the exact solution of a symmetric problem with 2392 cities was determined over a period of more than 27 hours on a powerful super computer [7]. It took roughly 3-4 years of CPU time on a large network of computers to determine the exact solution of the previously mentioned 7397-city problem [8].

Symmetric problems are usually more difficult to solve than asymmetric problems [9]. Today the 7397-city problem is the largest (nontrivial) symmetric problem that has been solved. In comparison, the optimal solution of a 500,000-city asymmetric problem has been reported [10].

2.3.2 Approximate algorithms

In contrast, the *approximate* algorithms obtain good solutions but do not guarantee that optimal solutions will be found. These algorithms are usually very simple and have (relative) short running times. Some of the algorithms give solutions that in average differs only by a few percent from the optimal solution. Therefore, if a small deviation from optimum can be accepted, it may be appropriate to use an approximate algorithm.

The class of approximate algorithms may be subdivided into the following three classes:

- *Tour construction algorithms*
- *Tour improvement algorithms*
- *Composite algorithms.*

The *tour construction algorithms* gradually build a tour by adding a new city at each step. The *tour improvement algorithms* improve upon a tour by performing various exchanges. The *composite algorithms* combine these two features.

A simple example of a tour construction algorithm is the so-called *nearest-neighbor algorithm* [11]: Start in an arbitrary city. As long as there are cities, that have not yet been visited, visit the nearest city that still has not appeared in the tour. Finally, return to the first city.

This approach is simple, but often too greedy. The first distances in the construction process are reasonable short, whereas the distances at the end of the process usually will be rather long. A lot of other construction algorithms have been developed to remedy this problem (see for example [2], [12] and [13]).

The tour improvement algorithms, however, have achieved the greatest success. A simple example of this type of algorithm is the so-called *2-opt algorithm*: Start with a given tour. Replace 2 links of the tour with 2 other links in such a way that the new tour length is shorter. Continue in this way until no more improvements are possible.

Figure 2.1 illustrates a 2-opt exchange of links, a so-called *2-opt move*. Note that a 2-opt move keeps the tour feasible and corresponds to a reversal of a subsequence of the cities.

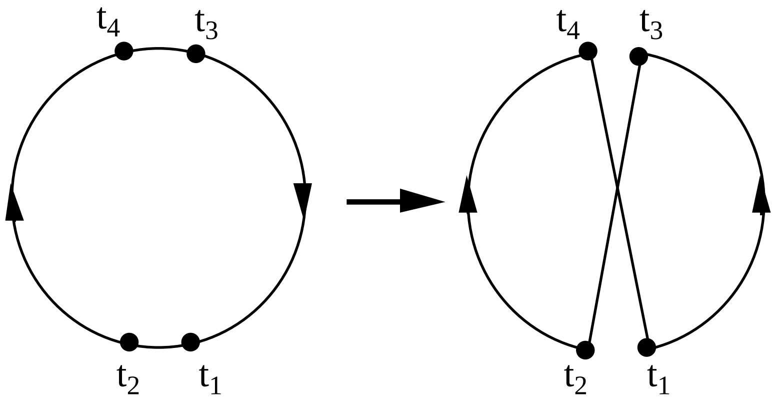

Figure 2.1 A 2-opt move

A generalization of this simple principle forms the basis for one the most effective approximate algorithms for solving the symmetric TSP, the *Lin-Kernighan algorithm* [1]. The original algorithm, as implemented by Lin and Kernighan in 1971, had an average running time of order $n^{2.2}$ and was able to find the optimal solutions for most problems with fewer than 100 cities.

However, the Lin-Kernighan algorithm is not simple to implement. In a survey paper from 1989 [14] the authors wrote that no other implementation of the algorithm at that time had shown as good efficiency as was obtained by Lin and Kernighan.

3. The Lin-Kernighan algorithm

3.1 The basic algorithm

The 2-opt algorithm is a special case of the λ-*opt* algorithm [15], where in each step λ links of the current tour are replaced by λ links in such a way that a shorter tour is achieved. In other words, in each step a shorter tour is obtained by deleting λ links and putting the resulting paths together in a new way, possibly reversing one ore more of them.

The λ-opt algorithm is based on the concept λ-*optimality*:

> A tour is said to be λ-*optimal* (or simply λ-*opt*) if it is impossible to obtain a shorter tour by replacing any λ of its links by any other set of λ links.

From this definition it is obvious that any λ-optimal tour is also λ'-optimal for 1 ≤ λ' ≤ λ. It is also easy to see that a tour containing n cities is optimal if and only if it is n-optimal.

In general, the larger the value of λ, the more likely it is that the final tour is optimal. For fairly large λ it appears, at least intuitively, that a λ-optimal tour should be optimal.

Unfortunately, the number of operations to test all λ-exchanges increases rapidly as the number of cities increases. In a naive implementation the testing of a λ-exchange has a time complexity of $O(n^{\lambda})$. Furthermore, there is no nontrivial upper bound of the number of λ–exchanges. As a result, the values λ = 2 and λ = 3 are the most commonly used. In one study the values λ = 4 and λ = 5 were used [16].

However, it is a drawback that λ must be specified in advance. It is difficult to know what λ to use to achieve the best compromise between running time and quality of solution.

Lin and Kernighan removed this drawback by introducing a powerful *variable* λ-*opt* algorithm. The algorithm changes the value of λ during its execution, deciding at each iteration what the value of λ should be. At each iteration step the algorithm examines, for ascending values of λ, whether an interchange of λ links may result in a shorter tour. Given that the exchange of r links is being considered, a series of tests is performed to determine whether r+1 link exchanges should be considered. This continues until some stopping conditions are satisfied.

At each step the algorithm considers a growing set of potential exchanges (starting with r = 2). These exchanges are chosen in such a way that a feasible tour may be formed at any stage of the process. If the exploration succeeds in finding a new shorter tour, then the actual tour is replaced with the new tour.

The Lin-Kernighan algorithm belongs to the class of so-called *local optimization algorithms* [17, 18]. The algorithm is specified in terms of *exchanges* (or *moves*) that can convert one tour into another. Given a feasible tour, the algorithm repeatedly performs exchanges that reduce the length of the current tour, until a tour is reached for which no exchange yields an improvement. This process may be repeated many times from initial tours generated in some randomized way. The algorithm is described below in more detail.

Let T be the current tour. At each iteration step the algorithm attempts to find two sets of links, $X = \{x_1, \ldots, x_r\}$ and $Y = \{y_1, \ldots, y_r\}$, such that, if the links of X are deleted from T and replaced by the links of Y, the result is a better tour. This interchange of links is called a *r-opt move*. Figure 3.1 illustrates a 3-opt move.

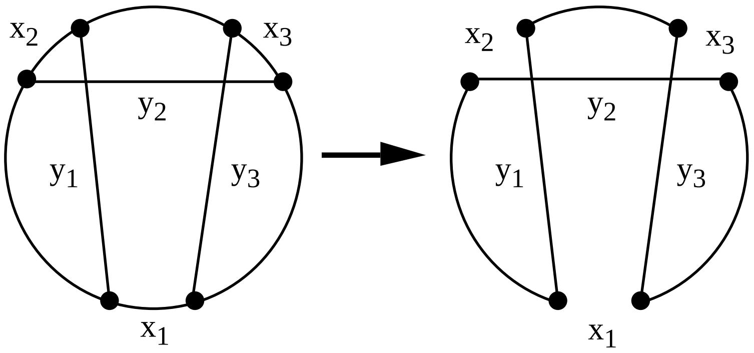

Figure 3.1 A 3-opt move

The two sets X and Y are constructed element by element. Initially X and Y are empty. In step i a pair of links, x_i and y_i, are added to X and Y, respectively.

In order to achieve a sufficient efficient algorithm, only links that fulfill the following criteria may enter X and Y.

(1) The sequential exchange criterion

x_i and y_i must share an endpoint, and so must y_i and x_{i+1}. If t_1 denotes one of the two endpoints of x_1, we have in general: $x_i = (t_{2i-1},t_{2i})$, $y_i = (t_{2i},t_{2i+1})$ and $x_{i+1} = (t_{2i+1},t_{2i+2})$ for $i \geq 1$. See Figure 3.2.

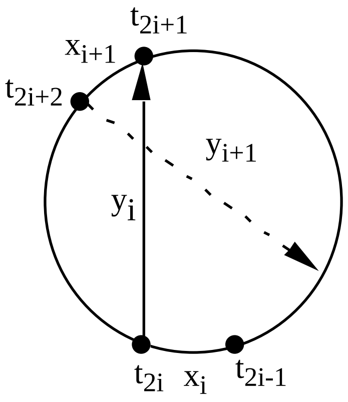

Figure 3.2. Restricting the choice of x_i, y_i, x_{i+1}, and y_{i+1}.

As seen, the sequence (x_1, y_1, x_2, y_2, x_3, …, x_r, y_r) constitutes a chain of adjoining links.

A necessary (but not sufficient) condition that the exchange of links X with links Y results in a tour is that the chain is closed, i.e., $y_r = (t_{2r},t_1)$. Such an exchange is called *sequential*.

Generally, an improvement of a tour may be achieved as a sequential exchange by a suitable numbering of the affected links. However, this is not always the case. Figure 3.3 shows an example where a sequential exchange is not possible.

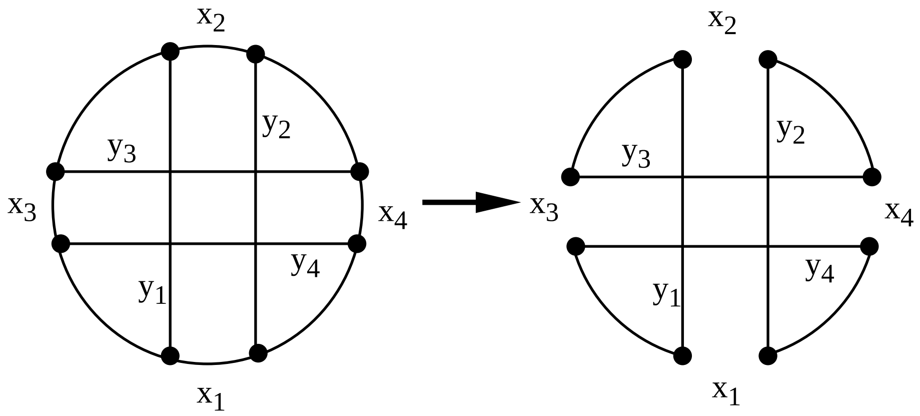

Figure 3.3 Nonsequential exchange (r = 4).

(2) The feasibility criterion

It is required that $x_i = (t_{2i-1},t_{2i})$ is chosen so that, if t_{2i} is joined to t_1, the resulting configuration is a tour. This feasibility criterion is used for $i \geq 3$ and guarantees that it is possible to *close up* to a tour. This criterion was included in the algorithm both to reduce running time and to simplify the coding.

(3) The positive gain criterion

It is required that y_i is always chosen so that the *gain*, G_i, from the proposed set of exchanges is positive. Suppose $g_i = c(x_i) - c(y_i)$ is the gain from exchanging x_i with y_i. Then G_i is the sum $g_1 + g_2 + ... + g_i$.

This stop criterion plays a great role in the efficiency of the algorithm. The demand that every partial sum, G_i, must be positive seems immediately to be too restrictive. That this, however, is not the case, follows from the following simple fact: If a sequence of numbers has a positive sum, there is a cyclic permutation of these numbers such that every partial sum is positive. The proof is simple and can be found in [1].

(4) The disjunctivity criterion

Finally, it is required that the sets X and Y are disjoint. This simplifies coding, reduces running time and gives an effective stop criterion.

Below is given an outline of the basic algorithm (a simplified version of the original algorithm).

1. Generate a random initial tour T.
2. Let i = 1. Choose t_1.
3. Choose $x_1 = (t_1,t_2)$ T.
4. Choose $y_1 = (t_2,t_3)$ T such that $G_1 > 0$.
   If this is not possible, go to Step 12.
5. Let i = i+1.
6. Choose $x_i = (t_{2i-1},t_{2i})$ T such that
   (a) if t_{2i} is joined to t_1, the resulting configuration is a tour, T', and
   (b) $x_i \neq y_s$ for all s < i.
   If T' is a better tour than T, let T = T' and go to Step 2.
7. Choose $y_i = (t_{2i},t_{2i+1})$ T such that
   (a) $G_i > 0$,
   (b) $y_i \neq x_s$ for all $s \leq i$, and
   (c) x_{i+1} exists.
   If such y_i exists, go to Step 5.
8. If there is an untried alternative for y_2, let i = 2 and go to Step 7.
9. If there is an untried alternative for x_2, let i = 2 and go to Step 6.
10. If there is an untried alternative for y_1, let i = 1 and go to Step 4.
11. If there is an untried alternative for x_1, let i = 1 and go to Step 3.
12. If there is an untried alternative for t_1, then go to Step 2.
13. Stop (or go to Step 1).

Figure 3.4. The basic Lin-Kernighan algorithm

Comments on the algorithm:

Step 1. A random tour is chosen as the starting point for the explorations.

Step 3. Choose a link $x_1 = (t_1,t_2)$ on the tour. When t_1 has been chosen, there are two choices for x_1. Here the verb 'choose' means 'select an untried alternative'. However, each time an improvement of the tour has been found (in Step 6), all alternatives are considered untried.

Step 6. There are two choices of x_i. However, for given y_{i-1} ($i \geq 2$) only one of these makes it possible to 'close' the tour (by the addition of y_i). The other choice results in two disconnected subtours. In only one case, however, such an unfeasible choice is allowed, namely for $i = 2$. Figure 3.5 shows this situation.

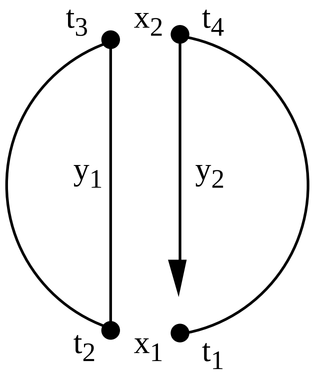

Figure 3.5 No close up at x_2.

If y_2 is chosen so that t_5 lies between t_2 and t_3, then the tour can be closed in the next step. But then t_6 may be on either side of t_5 (see Figure 3.6); the original algorithm investigated both alternatives.

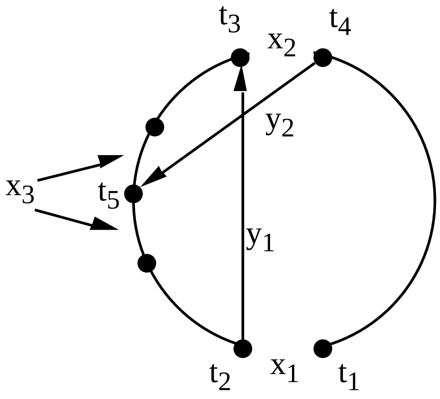

Figure 3.6 Two choices for x_3.

On the other hand, if y_2 is chosen so that t_5 lies between t_4 and t_1, there is only one choice for t_6 (it must lie between t_4 and t_5), and t_7 must lie between t_2 and t_3. But then t_8 can be on either side of t_7 (see Figure 3.7); the original algorithm investigated the alternative for which $c(t_7,t_8)$ is maximum.

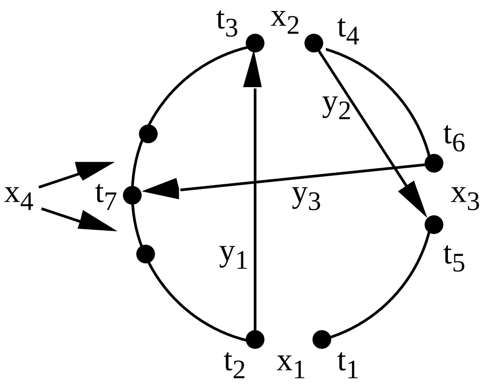

Figure 3.7 Unique choice for x_3. Limited choice of y_3. Two choices for x_4.

Condition (b) in Step 6 and Step 7 ensures that the sets X and Y are disjoint: y_i must not be a previously broken link, and x_i must not be a link previously added.

Steps 8-12. These steps cause backtracking. Note that backtracking is allowed only if no improvement has been found, and only at levels 1 and 2.

Step 13. The algorithm terminates with a solution tour when all values of t_1 have been examined without improvement. If required, a new random initial tour may be considered at Step 1.

The algorithm described above differs from the original one by its reaction on tour improvements. In the algorithm given above, a tour T is replaced by a shorter tour T' as soon as an improvement is found (in Step 6). In contrast, the original algorithm continues its steps by adding potential exchanges in order to find an even shorter tour. When no more exchanges are possible, or when $G_i \le G^*$, where G^* is the best improvement of T recorded so far, the search stops and the current tour T is replaced by the most advantageous tour. In their paper [1] Lin and Kernighan did not state their reasons for introducing this method. It complicates the coding and results neither in better solutions nor in shorter running times.

3.2 Lin and Kernighan's refinements

A bottleneck of the algorithm is the search for links to enter the sets X and Y. In order to increase efficiency, special care therefore should be taken to limit this search. Only exchanges that have a reasonable chance of leading to a reduction of tour length should be considered.

The basic algorithm as presented in the preceding section limits its search by using the following four rules:

(1) Only sequential exchanges are allowed.

(2) The provisional gain must be positive.

(3) The tour can be 'closed' (with one exception, $i = 2$).

(4) A previously broken link must not be added, and a previously added link must not be broken.

To limit the search even more Lin and Kernighan refined the algorithm by introducing the following rules:

(5) The search for a link to enter the tour, $y_i = (t_{2i},t_{2i+1})$, is limited to the five nearest neighbors to t_{2i}.

(6) For $i \geq 4$, no link, x_i, on the tour must be broken if it is a common link of a small number (2-5) of solution tours.

(7) The search for improvements is stopped if the current tour is the same as a previous solution tour.

Rules 5 and 6 are heuristic rules. They are based on expectations of which links are likely to belong to an optimal tour. They save running time, but sometimes at the expense of not achieving the best possible solutions.

Rule 7 also saves running time, but has no influence on the quality of solutions being found. If a tour is the same as a previous solution tour, there is no point in attempting to improve it further. The time needed to check that no more improvements are possible (the checkout time) may therefore be saved. According to Lin and Kernighan the time saved in this way it typically 30 to 50 percent of running time.

In addition to these refinements, whose purpose is primarily to *limit* the search, Lin and Kernighan added some refinements whose purpose is primarily to *direct* the search. Where the algorithm has a choice of alternatives, heuristic rules are used to give priorities to these alternatives. In cases where only one of the alternatives must be chosen, the one with the highest priority is

chosen. In cases where several alternatives must be tried, the alternatives are tried in descending priority order (using backtracking). To be more specific, the following rules are used:

(8) When link y_i ($i \geq 2$) is to be chosen, each possible choice is given the priority $c(x_{i+1}) - c(y_i)$.

(9) If there are two alternatives for x_4, the one where $c(x_4)$ is highest is chosen.

Rule 8 is a heuristic rule for ranking the links to be added to Y. The priority for y_i is the length of the next (unique) link to be broken, x_{i+1}, if y_i is included in the tour, minus the length of y_i. In this way, the algorithm is provided with some look-ahead. By maximizing the quantity $c(x_{i+1}) - c(y_i)$, the algorithm aims at breaking a long link and including a short link.

Rule 9 deals with the special situation in Figure 3.7 where there are two choices for x_4. The rule gives preference to the longest link in this case. In three other cases, namely for x_1, x_2, and sometimes x_3 (see Figure 3.6) there are two alternatives available. In these situations the algorithm examines both choices using backtracking (unless an improved tour was found). In their paper Lin and Kernighan do not specify the sequence in which the alternatives are examined.

As a last refinement, Lin and Kernighan included a limited defense against the situations where only nonsequential exchanges may lead to a better solution. After a local optimum has been found, the algorithm tests, among the links allowed to be broken, whether it is possible to make a further improvement by a nonsequential 4-opt change (as shown in Figure 3.3). Lin and Kernighan pointed out that the effect of this post optimization procedure varies substantially from problem to problem. However, the time used for the test is small relative to the total running time, so it is a cheap insurance.

4. The modified Lin-Kernighan algorithm

Lin and Kernighan's original algorithm was reasonably effective. For problems with up to 50 cities, the probability of obtaining optimal solutions in a single trial was close to 100 percent. For problems with 100 cities the probability dropped to between 20 and 30 percent. However, by running a few trials, each time starting with a new random tour, the optimum for these problems could be found with nearly 100 percent assurance.

The algorithm was evaluated on a spectrum of problems, among these a drilling problem with 318 points. Due to computer-storage limitations, the problem was split into three smaller problems. A solution tour was obtained by solving the subproblems separately, and finally joining the three tours. At the time when Lin and Kernighan wrote their paper (1971), the optimum for this problem was unknown. Now that the optimum is known, it may be noted that their solution was 1.3 percent above optimum.

In the following, a modified and extended version of their algorithm is presented. The new algorithm is a considerable improvement of the original algorithm. For example, for the mentioned 318-city problem the optimal solution is now found in a few trials (approximately 2), and in a very short time (about one second on a 300 MHz G3 Power Macintosh). In general, the quality of solutions achieved by the algorithm is very impressive. The algorithm has been able to find optimal solutions for all problem instances we have been able to obtain, including a 7397-city problem (the largest nontrivial problem instance solved to optimality today).

The increase in efficiency is primarily achieved by a revision of Lin and Kernighan's heuristic rules for restricting and directing the search. Even if their heuristic rules seem natural, a critical analysis shows that they suffer from considerable defects.

4.1 Candidate sets

A central rule in the original algorithm is the heuristic rule that restricts the inclusion of links in the tour to the five nearest neighbors to a given city (Rule 5 in Section 3.2). This rule directs the search against short tours and reduces the search effort substantially. However, there is a certain risk that the application of this rule may prevent the optimal solution from being found. If an optimal solution contains one link, which is not connected to the five nearest neighbors of its two end cities, then the algorithm will have difficulties in obtaining the optimum.

The inadequacy of this rule manifests itself particularly clearly in large problems. For example, for a 532-city problem [19] one of the links in the optimal solution is the 22nd nearest neighbor city for one of its end points. So in order to find the optimal solution to this problem, the number of nearest neigh-

bors to be considered ought to be at least 22. Unfortunately, this enlargement of the set of candidates results in a substantial increase in running time.

The rule builds on the assumption that the shorter a link is, the greater is the probability that it belongs to an optimal tour. This seems reasonable, but used too restrictively it may result in poor tours.

In the following, a measure of *nearness* is described that better reflects the chances of a given link being a member of an optimal tour. This measure, called α-*nearness*, is based on sensitivity analysis using minimum spanning trees.

First, some well-known graph theoretical terminology is reviewed.

Let G = (N, E) be a undirected weighted graph where N = {1, 2, ..., n} is the set of *nodes* and E = {(i,j)| i   N, j   N} is the set of *edges*. Each edge (i,j) has associated a *weight* c(i,j).

A *path* is a set of edges $\{(i_1,i_2), (i_2,i_3), ..., (i_{k-1},i_k)\}$ with $i_p \neq i_q$ for all $p \neq q$.

A *cycle* is a set of edges $\{(i_1,i_2), (i_2,i_3), ..., (i_k,i_1)\}$ with $i_p \neq i_q$ for $p \neq q$.

A *tour* is a cycle where k = n.

For any subset S   E the *length* of S, L(S), is given by $L(S) = \sum_{(i,j)\ \ S} c(i,j)$.

An *optimal tour* is a tour of minimum length. Thus, the symmetric TSP can simply be formulated as: “Given a weighted graph G, determine an optimal tour of G”.

A graph G is said to be *connected* if it contains for any pair of nodes a path connecting them.

A *tree* is a connected graph without cycles. A *spanning tree* of a graph G with n nodes is a tree with n-1 edges from G. A *minimum spanning tree* is a spanning tree of minimum length.

Now the important concept of a *1-tree* may be defined.

> A *1-tree* for a graph G = (N, E) is a spanning tree on the node set N\{1} combined with two edges from E incident to node 1.

The choice of node 1 as a special node is arbitrary. Note that a 1-tree is not a tree since it contains a cycle (containing node 1; see Figure 4.1).

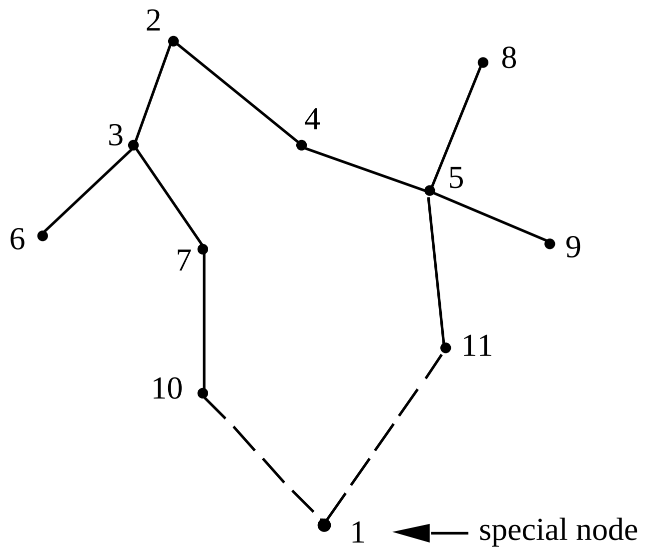

Figure 4.1 A 1-tree.

A *minimum 1-tree* is a 1-tree of minimum length.

The *degree* of a node is the number of edges incident to the node.

It is easy to see [20, 21] that

(1) an optimal tour is a minimum 1-tree where every node has degree 2;

(2) if a minimum 1-tree is a tour, then the tour is optimal.

Thus, an alternative formulation of the symmetric TSP is: “Find a minimum 1-tree all whose nodes have degree 2”.

Usually a minimum spanning tree contains many edges in common with an optimal tour. An optimal tour normally contains between 70 and 80 percent of the edges of a minimum 1-tree. Therefore, minimum 1-trees seem to be well suited as a heuristic measure of ‘nearness’. Edges that belong, or ‘nearly belong', to a minimum 1-tree, stand a good chance of also belonging to an optimal tour. Conversely, edges that are ‘far from’ belonging to a minimum 1-tree have a low probability of also belonging to an optimal tour. In the Lin-Kernighan algorithm these ‘far’ edges may be excluded as candidates to enter a tour. It is expected that this exclusion does not cause the optimal tour to be missed.

More formally, this measure of nearness is defined as follows:

> Let T be a minimum 1-tree of length L(T) and let $T^+(i,j)$ denote a minimum 1-tree required to contain the edge (i,j). Then the α–*nearness* of an edge (i,j) is defined as the quantity
>
> $$\alpha(i,j) = L(T^+(i,j)) - L(T).$$

That is, given the length of (any) minimum 1-tree, the α−nearness of an edge is the increase of length when a minimum 1-tree is required to contain this edge.

It is easy to verify the following two simple properties of α:

(1) $\alpha(i,j) \geq 0$.

(2) If (i,j) belongs to some minimum 1-tree, then $\alpha(i,j) = 0$.

The α-measure can be used to systematically identify those edges that could conceivably be included in an optimal tour, and disregard the remainder. These 'promising edges', called the *candidate set*, may, for example, consist of the k α-nearest edges incident to each node, and/or those edges having an α-nearness below a specified upper bound.

In general, using the α-measure for specifying the candidate set is much better than using nearest neighbors. Usually, the candidate set may be smaller, without degradation of the solution quality.

The use of α-nearness in the construction of the candidate set implies computations of α-values. The efficiency, both in time of space, of these computations is therefore important. The method is not of much practical value, if the computations are too expensive. In the following an algorithm is presented that computes all α-values. The algorithm has time complexity $O(n^2)$ and uses space O(n).

Let G = (N, E) be a *complete* graph, that is, a graph where for all nodes i and j in N there is an edge (i,j) in E. The algorithm first finds a minimum 1-tree for G. This can be done by determination of a minimum spanning tree that contains the nodes {2, 3, …, n}, followed by the addition of the two shortest edges incident to node 1. The minimum spanning tree may, for example, be determined using Prim's algorithm [22], which has a run time complexity of $O(n^2)$. The additional two edges may be determined in time O(n). Thus, the complexity of this first part is $O(n^2)$.

Next, the nearness α(i,j) is determined for all edges (i,j). Let T be a minimum 1-tree. From the definition of a minimum spanning tree, it is easy to see that a minimum spanning tree $T^+(i,j)$ containing the edge (i,j) may be determined from T using the following action rules:

(a) If (i,j) belongs to T, then $T^+(i,j)$ is equal to T.

(b) Otherwise, if (i,j) has 1 as end node (i = 1   j = 1), then $T^+(i,j)$ is obtained from T by replacing the longest of the two edges of T incident to node 1 with (i,j).

(c) Otherwise, insert (i,j) in T. This creates a cycle containing (i,j) in the spanning tree part of T. Then $T^+(i,j)$ is obtained by removing the longest of the other edges on this cycle.

Cases a and b are simple. With a suitable representation of 1-trees they can both be treated in constant time.

Case c is more difficult to treat efficiently. The number of edges in the produced cycles is O(n). Therefore, with a suitable representation it is possible to treat each edge with time complexity O(n). Since $O(n^2)$ edges must be treated this way, the total time complexity becomes $O(n^3)$, which is unsatisfactory.

However, it is possible to obtain a total complexity of $O(n^2)$ by exploiting a simple relation between the α-values [23, 24].

Let β(i,j) denote the length of the edge to be removed from the spanning tree when edge (i,j) is added. Thus α(i,j) = c(i,j) - β(i,j). Then the following fact may be exploited (see Figure 4.2). If (j_1,j_2) is an edge of the minimum spanning tree, i is one of the remaining nodes and j_1 is on that cycle that arises by adding the edge (i,j_2) to the tree, then $\beta(i,j_2)$ may be computed as the maximum of $\beta(i,j_1)$ and $c(j_1,j_2)$.

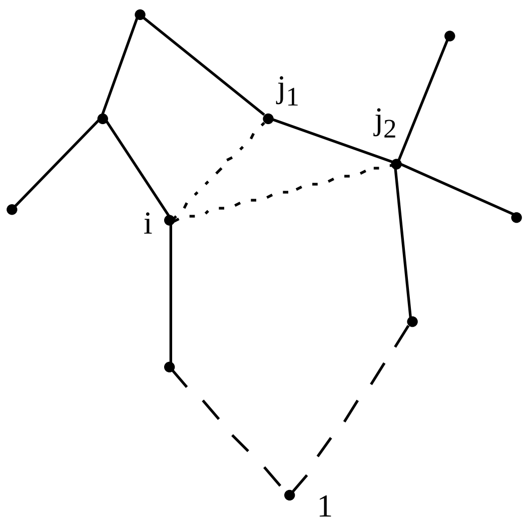

Figure 4.2 $\beta(i,j_2)$ *may be computed from* $\beta(i,j_1)$.

Thus, for a given node i all the values β(i,j), j = 1, 2, ..., n, can be computed with a time complexity of O(n), if only the remaining nodes are traversed in a suitable sequence. It can be seen that such a sequence is produced as a by-product of Prim’s algorithm for constructing minimum spanning trees, namely a topological order, in which every node's descendants in the tree are placed after the node. The total complexity now becomes $O(n^2)$.

Figure 4.3 sketches in C-style notation an algorithm for computing β(i,j) for i ≠ 1, j ≠ 1, i ≠ j. The algorithm assumes that the father of each node j in the tree, dad[j], precedes the node (i.e., dad[j] = i   i < j).

```
for (i = 2; i < n; i++) {
    β[i][i] = -∞;
    for (j = i+1; j <= n; j++)
        β[i][j] = β[j][i] = max(β[i][dad[j]], c(j,dad[j]));
}
```

Figure 4.3 Computation of β*(i,j) for i ≠1, j ≠1, i ≠ j.*

Unfortunately this algorithm needs space $O(n^2)$ for storing β-values. Some space may be saved by storing the c- and β-values in one quadratic matrix, so that, for example, the c-values are stored in the lower triangular matrix, while the β-values are stored in the upper triangular matrix. For large values of n, however, storage limitations may make this approach impractical.

Half of the space may be saved if the c-values are not stored, but computed when needed (for example as Euclidean distances). The question is whether it is also possible to save the space needed for the β-values At first sight it would seem that the β-values must be stored in order to achieve $O(n^2)$ time complexity for their computation. That this is not the case will now be demonstrated.

The algorithm, given in Figure 4.4, uses two one-dimensional auxiliary arrays, `b` and `mark`. Array `b` corresponds to the β-matrix but only contains β-values for a given node `i`, i.e., `b[j] = β(i,j)`. Array `mark` is used to indicate that `b[j]` has been computed for node `i`.

The determination of `b[j]` is done in two phases. First, `b[j]` is computed for all nodes `j` on the path from node `i` to the root of the tree (node `2`). These nodes are marked with `i`. Next, a forward pass is used to compute the remaining `b`-values. The α-values are available in the inner loop.

```
for (i = 2; i <= n; i++)
    mark[i] = 0;
for (i = 2; i <= n; i++) {
    b[i] = -∞;
    for (k = i; k != 2; k = j) {
        j = dad[k];
        b[j] = max(b[k], c(k,j));
        mark[j] = i;
    }
    for (j = 2; j <= n; j++) {
        if (j != i) {
            if (mark[j] != i)
                b[j] = max(b[dad[j]], c(j,dad[j]));
            /* α(i,j) is now available as c(i,j) - b[j] */
        }
    }
}
```

Figure 4.4 Space efficient computation of α.

It is easy to see that this algorithm has time complexity $O(n^2)$ and uses space $O(n)$.

The α-values provide a good estimate of the edges' chances of belonging to an optimal tour. The smaller α is for an edge, the more promising is this edge. Using α-nearness it is often possible to limit the search to relative few of the α-nearest neighbors of a node, and still obtain an optimal tour. Computational tests have shown that the α-measure provides a better estimate of the likelihood of an edge being optimal than the usual c-measure. For example, for the 532-city problem the worst case is an optimal edge being the 22nd

c-nearest edge for a node, whereas the worst case when using the α-measure is an optimal edge being the 14th α-nearest. The average rank of the optimal edges among the candidate edges is reduced from 2.4 to 2.1.

This seems to be quite satisfactory. However, the α-measure can be improved substantially by making a simple transformation of the original cost matrix. The transformation is based on the following observations [21]:

(1) Every tour is a 1-tree. Therefore the length of a minimum 1-tree is a lower bound on the length of an optimal tour.

(2) If the length of all edges incident to a node are changed with the same amount, π, any optimal tour remains optimal. Thus, if the cost matrix C= (c_{ij}) is transformed to D = (d_{ij}), where

$$d_{ij} = c_{ij} + \pi_i + \pi_j,$$

then an optimal tour for the D is also an optimal tour for C. The length of every tour is increased by $2\Sigma\pi_i$. The transformation leaves the TSP invariant, but usually changes the minimum 1-tree.

(3) If T_π is a minimum 1-tree with respect to D, then its length, $L(T_\pi)$, is a lower bound on the length of an optimal tour for D. Therefore $w(\pi) = L(T_\pi) - 2\Sigma\pi_i$ is lower bound on the length of an optimal tour for C.

The aim is now to find a transformation, C → D, given by the vector $\pi = (\pi_1, \pi_2, \ldots, \pi_n)$, that maximizes the lower bound $w(\pi) = L(T_\pi) - 2\Sigma\pi_i$.

If T_π becomes a tour, then the exact optimum has been found. Otherwise, it appears, at least intuitively, that if $w(\pi) > w(0)$, then α-values computed from D are better estimates of edges being optimal than α-values computed from C.

Usually, the maximum of w(π) is close to the length of an optimal tour. Computational experience has shown that this maximum typically is less than 1 percent below optimum. However, finding maximum for w(π) is not a trivial task. The function is piece-wise linear and concave, and therefore not differentiable everywhere.

A suitable method for maximizing w(π) is *subgradient optimization* [21] (a subgradient is a generalization of the gradient concept). It is an iterative method in which the maximum is approximated by stepwise changes of π.

At each step π is changed in the direction of the subgradient, i.e., $\pi^{k+1} = \pi^k + t^k v^k$, where v^k is a subgradient vector, and t^k is a positive scalar, called the *step size.*

For the actual maximization problem it can be shown that $v^k = d^k - 2$ is a subgradient vector, where d^k is a vector having as its elements the degrees of the nodes in the current minimum 1-tree. This subgradient makes the algorithm strive towards obtaining minimum 1-trees with node degrees equal to 2, i.e., minimum 1-trees that are tours. Edges incident to a node with degree 1 are made shorter. Edges incident to a node with degree greater than 2 are made longer. Edges incident to a node with degree 2 are not changed.

The π-values are often called *penalties*. The determination of a (good) set of penalties is called an *ascent*.

Figure 4.5 shows a subgradient algorithm for computing an approximation W for the maximum of $w(\pi)$.

1. Let $k = 0$, $\pi^0 = 0$ and $W = -\infty$.
2. Find a minimum 1-tree, T_π^k.
3. Compute $w(\pi^k) = L(T_\pi^k) - 2\Sigma\pi_i$.
4. Let $W = \max(W, w(\pi^k))$.
5. Let $v^k = d^k - 2$, where d^k contains the degrees of nodes in T_π^k.
6. If $v^k = 0$ (T_π^k is an optimal tour), or a stop criterion is satisfied, then stop.
7. Choose a step size, t^k.
8. Let $\pi^{k+1} = \pi^k + t^k v^k$.
9. Let $k = k + 1$ and go to Step 2.

Figure 4.5 Subgradient optimization algorithm.

It has been proven [26] that W will always converge to the maximum of $w(\pi)$, if $t^k \rightarrow 0$ for $k \rightarrow \infty$ and $\sum t^k = \infty$. These conditions are satisfied, for example, if t^k is t^0/k, where t^0 is some arbitrary initial step size. But even if convergence is guaranteed, it is very slow.

The choice of step size is a very crucial decision from the viewpoint of algorithmic efficiency or even adequacy. Whether convergence is guaranteed is often not important, as long as good approximations can be obtained in a short time.

No general methods to determine an optimum strategy for the choice of step size are known. However, many strategies have been suggested that are quite effective in practice [25, 27, 28, 29, 30, 31]. These strategies are heuristics, and different variations have different effects on different problems. In the present implementation of the modified Lin-Kernighan algorithm the following strategy was chosen (inspired by [27] and [31]):

- The step size is constant for a fixed number of iterations, called a *period*.
- When a period is finished, both the length of the period and the step size are halved.
- The length of the first period is set to n/2, where n is the number of cities.
- The initial step size, t^0, is set to 1, but is doubled in the beginning of the first period until W does not increase, i.e., $w(\pi^k) \le w(\pi^{k-1})$. When this happens, the step size remains constant for the rest of the period.
- If the last iteration of a period leads to an increment of W, then the period is doubled.
- The algorithm terminates when either the step size, the length of the period or v^k becomes zero.

Furthermore, the basic subgradient algorithm has been changed on two points (inspired by [13]):

- The updating of π, i.e., $\pi^{k+1} = \pi^k + t^k v^k$, is replaced by

  $$\pi^{k+1} = \pi^k + t^k(0.7v^k + 0.3v^{k-1}), \text{ where } v^{-1} = v^0.$$

- The special node for the 1-tree computations is not fixed. A minimum 1-tree is determined by computing a minimum spanning tree and then adding an edge corresponding to the second nearest neighbor of one of the leaves of the tree. The leaf chosen is the one that has the longest second nearest neighbor distance.

Practical experiments have shown that these changes lead to better bounds.

Having found a penalty vector π, that maximizes $w(\pi)$, the transformation given by π of the original cost matrix C will often improve the α-measure substantially. For example, for the 532-city problem every edge of the optimal tour is among the 5 α-nearest neighbors for at least one of its endpoints. The improvement of the α-measure reduces the average rank of the optimal edges among the candidate edges from 2.1 to 1.7. This is close to the ideal value of 1.5 (when every optimal edge has rank 1 or 2).

Table 4.1 shows the percent of optimal edges having a given rank among the nearest neighbors with respect to the c-measure, the α-measure, and the improved α-measure, respectively.

rank	c	α (π = 0)	Improved α
1	43.7	43.7	47.0
2	24.5	31.2	39.5
3	14.4	13.0	9.7
4	7.3	6.4	2.3
5	3.3	2.5	0.9
6	2.9	1.4	0.1
7	1.1	0.4	0.3
8	0.7	0.7	0.2
9	0.7	0.2	
10	0.3	0.1	
11	0.2	0.3	
12	0.2		
13	0.3		
14	0.2	0.1	
15			
16			
17			
18			
19	0.1		
20			
21			
22	0.1		
$rank_{avg}$	2.4	2.1	1.7

Table 4.1. The percentage of optimal edges among candidate edges for the 532-city problem.

It appears from the table that the transformation of the cost matrix has the effect that optimal edges come ‘nearer’, when measured by their α-values. The transformation of the cost matrix ‘conditions’ the problem, so to speak. Therefore, the transformed matrix is also used during the Lin-Kernighan search process. Most often the quality of the solutions is improved by this means.

The greatest advantage of the α-measure, however, is its usefulness for the construction of the candidate set. By using the α-measure the cardinality of the candidate set may generally be small without reducing the algorithm’s ability to find short tours. Thus, in all test problems the algorithm was able to find optimal tours using as candidate edges only edges the 5 α-nearest edges incident to each node. Most of the problems could even be solved when search was restricted to only the 4 α-nearest edges.

The candidate edges of each node are sorted in ascending order of their α-values. If two edges have the same α-value, the one with the smallest cost, c_{ij}, comes first. This ordering has the effect that candidate edges are considered for inclusion in a tour according to their 'promise' of belonging to an optimal tour. Thus, the α-measure is not only used to *limit* the search, but also to *focus* the search on the most promising areas.

To speed up the search even more, the algorithm uses a *dynamic* ordering of the candidates. Each time a shorter tour is found, all edges shared by this new tour and the previous shortest tour become the first two candidate edges for their end nodes.

This method of selecting candidates was inspired by Stewart [32], who demonstrated how minimum spanning trees could be used to accelerate 3-opt heuristics. Even when subgradient optimization is not used, candidate sets based on minimum spanning trees usually produce better results than nearest neighbor candidate sets of the same size.

Johnson [17] in an alternative implementation of the Lin-Kernighan algorithm used precomputed candidate sets that usually contained more than 20 (ordinary) nearest neighbors of each node. The problem with this type of candidate set is that the candidate subgraph need not be connected even when a large fraction of all edges is included. This is, for example, the case for geometrical problems in which the point sets exhibit clusters. In contrast, a minimum spanning tree is (by definition) always connected.

Other candidate sets may be considered. An interesting candidate set can be obtained by exploiting the Delaunay graph [13, 33]. The Delaunay graph is connected and may be computed in linear time, on the average. A disadvantage of this approach, however, is that candidate sets can only be computed for geometric problem instances. In contrast, the α-measure is applicable in general.

4.2 Breaking of tour edges

A candidate set is used to prune the search for edges, Y, to be included in a tour. Correspondingly, the search of edges, X, to be excluded from a tour may be restricted. In the actual implementation the following simple, yet very effective, pruning rules are used:

(1) The first edge to be broken, x_1, must not belong to the currently best solution tour. When no solution tour is known, that is, during the determination of the very first solution tour, x_1 must not belong to the minimum 1-tree.

(2) The last edge to be excluded in a basic move must not previously have been included in the current chain of basic moves.

The first rule prunes the search already at level 1 of the algorithm, whereas the original algorithm of Lin and Kernighan prunes at level 4 and higher, and only if an edge to be broken is a common edge of a number (2-5) of solution tours. Experiments have shown that the new pruning rule is more effective. In addition, it is easier to implement.

The second rule prevents an infinite chain of moves. The rule is a relaxation of Rule 4 in Section 3.2.

4.3 Basic moves

Central in the Lin-Kernighan algorithm is the specification of allowable moves, that is, which subset of r-opt moves to consider in the attempt to transform a tour into a shorter tour.

The original algorithm considers only r-opt moves that can be decomposed into a 2- or 3-opt move followed by a (possibly empty) sequence of 2-opt moves. Furthermore, the r-opt move must be *sequential* and *feasible*, that is, it must be a connected chain of edges where edges removed alternate with edges added, and the move must result in a feasible tour. Two minor deviations from this general scheme are allowed. Both have to do with 4-opt moves. First, in one special case the first move of a sequence may be a sequential 4-opt move (see Figure 3.7); the following moves must still be 2-opt moves. Second, nonsequential 4-opt moves are tried when the tour can no longer be improved by sequential moves (see Figure 3.3).

The new modified Lin-Kernighan algorithm revises this basic search structure on several points.

First and foremost, the basic move is now a sequential 5-opt move. Thus, the moves considered by the algorithm are sequences of one or more 5-opt moves. However, the construction of a move is stopped immediately if it is discovered that a close up of the tour results in a tour improvement. In this way the algorithm attempts to ensure 2-, 3-, 4- as well as 5-optimality.

Using a 5-opt move as the basic move broadens the search and increases the algorithm's ability to find good tours, at the expense of an increase of running times. However, due to the use of small candidate sets, run times are only increased by a small factor. Furthermore, computational experiments have shown that backtracking is no longer necessary in the algorithm (except, of course, for the first edge to be excluded, x_1). The removal of backtracking reduces runtime and does not degrade the algorithm's performance significantly. In addition, the implementation of the algorithm is greatly simplified.

The new algorithm's improved performance compared with the original algorithm is in accordance with observations made by Christofides and Eilon [16]. They observed that 5-optimality should be expected to yield a relatively superior improvement over 4-optimality compared with the improvement of 4-optimality over 3-optimality.

Another deviation from the original algorithm is found in the examination of nonsequential exchanges. In order to provide a better defense against possible improvements consisting of nonsequential exchanges, the simple nonsequential 4-opt move of the original algorithm has been replaced by a more powerful set of nonsequential moves.

This set consists of

- any nonfeasible 2-opt move (producing two cycles) followed by any 2- or 3-opt move, which produces a feasible tour (by joining the two cycles);

- any nonfeasible 3-opt move (producing two cycles) followed by any 2-opt move, which produces a feasible tour (by joining the two cycles).

As seen, the simple nonsequential 4-opt move of the original algorithm belongs to this extended set of nonsequential moves. However, by using this set of moves, the chances of finding optimal tours are improved. By using candidate sets and the "positive gain criterion" the time for the search for such nonsequential improvements of the tour is small relative to the total running time.

Unlike the original algorithm the search for nonsequential improvements is not only seen as a post optimization maneuver. That is, if an improvement is found, further attempts are made to improve the tour by ordinary sequential as well as nonsequential exchanges.

4.4 Initial tours

The Lin-Kernighan algorithm applies edge exchanges several times to the same problem using different initial tours.

In the original algorithm the initial tours are chosen at random. Lin and Kernighan concluded that the use of a construction heuristic only wastes time. Besides, construction heuristics are usually deterministic, so it may not be possible to get more than one solution.

However, the question of whether or not to use a construction heuristic is not that simple to answer. Adrabinsky and Syslo [34], for instance, found that the farthest insertion construction heuristic was capable of producing good initial tours for the Lin-Kernighan algorithm. Perttunen [35] found that the

Clarke and Wright savings heuristic [36] in general improved the performance of the algorithm. Reinelt [13] also found that is better not to start with a random tour. He proposed using locally good tours containing some major errors, for example the heuristics of Christofides [37]. However, he also observed that the difference in performance decreases with more elaborate versions of the Lin-Kernighan algorithm.

Experiments with various implementations of the new modified Lin-Kernighan algorithm have shown that the quality of the final solutions does not depend strongly on the initial tours. However, significant reduction in run time may be achieved by choosing initial tours that are close to being optimal.

In the present implementation the following simple construction heuristic is used:

1. Choose a random node i.

2. Choose a node j, not chosen before, as follows:
   If possible, choose j such that
   (a) (i,j) is a candidate edge,
   (b) $\alpha(i,j) = 0$, and
   (c) (i,j) belongs to the current best tour.
   Otherwise, if possible, choose j such that (i,j) is a candidate edge.
   Otherwise, choose j among those nodes not already chosen.
3. Let i = j. If not all nodes have been chosen, then go to Step 2.

If more than one node may be chosen at Step 2, the node is chosen at random among the alternatives. The sequence of chosen nodes constitutes the initial tour.

This construction procedure is fast, and the diversity of initial solutions is large enough for the edge exchange heuristics to find good final solutions.

4.5 Specification of the modified algorithm

This section presents an overview of the modified Lin-Kernighan algorithm. The algorithm is described top-down using the C programming language.

Below is given a sketch of the main program.

```
void main() {
    ReadProblemData();
    CreateCandidateSet();
    BestCost = DBL_MAX;
    for (Run = 1; Run <= Runs; Run++) {
        double Cost = FindTour();
        if (Cost < BestCost) {
           RecordBestTour();
           BestCost = Cost;
        }
    }
    PrintBestTour();
}
```

First, the program reads the specification of the problem to be solved and creates the candidate set. Then a specified number (`Runs`) of local optimal tours is found using the modified Lin-Kernighan heuristics. The best of these tours is printed before the program terminates.

The creation of the candidate set is based on α-nearness.

```
void CreateCandidateSet() {
    double LowerBound = Ascent();
    long MaxAlpha = Excess * fabs(LowerBound);
    GenerateCandidates(MaxCandidates, MaxAlpha);
}
```

First, the function `Ascent` determines a lower bound on the optimal tour length using subgradient optimization. The function also transforms the original problem into a problem in which α-values reflect the likelihood of edges being optimal. Next, the function `GenerateCandidates` computes the α-values and associates to each node a set of incident candidate edges. The edges are ranked according to their α-values. The parameter `MaxCandidates` specifies the maximum number of candidate edges allowed for each node, and `MaxAlpha` puts an upper limit on their α-values. The value of `MaxAlpha` is set to some fraction, `Excess`, of the lower bound.

The pseudo code of the function `Ascent` shown below should be reasonable self-explanatory. It follows the description given in Section 4.1. The `V`-value of a node is its degree minus 2. Therefore, `Norm` being the sum of squares of all `V`-values, is a measure of a minimum 1-tree's discrepancy from a tour. If `Norm` is zero, then the 1-tree constitutes a tour, and an optimal tour has been found. In order to speed up the computations the algorithm uses candidate sets in the computations of minimum 1-trees.

```
double Ascent() {
    Node *N;
    double BestW, W;
    int Period = InitialPeriod, P, InitialPhase = 1;

    W = Minimum1TreeCost();
    if (Norm == 0) return W;
    GenerateCandidates(AscentCandidates, LONG_MAX);
    BestW = W;
    ForAllNodes(N) {
        N->LastV = N->V;
        N->BestPi = N->Pi;
    }
    for (T = InitialStepSize; T > 0; Period /= 2, T /= 2) {
        for (P = 1; T > 0 && P <= Period; P++) {
            ForAllNodes(N) {
                if (N->V != 0)
                    N->Pi += T*(7*N->V + 3*N->LastV)/10;
                N->LastV = N->V;
            }
            W = Minimum1TreeCost();
            if (Norm == 0) return W;
            if (W > BestW) {
                BestW = W;
                ForAllNodes(N)
                    N->BestPi = N->Pi;
                if (InitialPhase) T *= 2;
                if (P == Period) Period *= 2;
            }
            else if (InitialPhase && P > InitalPeriod/2) {
                InitialPhase = 0;
                P = 0;
                T = 3*T/4;
            }
        }
    }
    ForAllNodes(N)
        N->Pi = N->BestPi;
    return Minimum1TreeCost();
}
```

Below is shown the pseudo code of the function `GenerateCandidates`. For each node at most `MaxCandidates` candidate edges are determined. This upper limit, however, may be exceeded if a "symmetric" neighborhood is desired (`SymmetricCandidates != 0`) in which case the candidate set is complemented such that every candidate edge is associated to both its two end nodes.

```
void GenerateCandidates(long MaxCandidates, long MaxAlpha) {
    Node *From, *To;
    long Alpha;
    Candidate *Edge;

    ForAllNodes(From)
        From->Mark = 0;
    ForAllNodes(From) {
        if (From != FirstNode) {
            From->Beta = LONG_MIN;
            for (To = From; To->Dad != 0; To = To->Dad) {
                To->Dad->Beta = max(To->Beta, To->Cost);
                To->Dad->Mark = From;
            }
        }
        ForAllNodes(To, To != From) {
            if (From == FirstNode)
                Alpha = To == From->Father ? 0 :
                        C(From,To) - From->NextCost;
            else if (To == FirstNode)
                Alpha = From == To->Father ? 0:
                        C(From,To) - To->NextCost;
            else {
                if (To->Mark != From)
                    To->Beta = max(To->Dad->Beta, To->Cost);
                Alpha = C(From,To) - To->Beta;
           }
           if (Alpha <= MaxAlpha)
              InsertCandidate(To, From->CandidateSet);
        }
    }
    if (SymmetricCandidates)
        ForAllNodes(From)
            ForAllCandidates(To, From->CandidateSet)
                if (!IsMember(From, To->CandidateSet))
                    InsertCandidate(From, To->CandidateSet);
}
```

After the candidate set has been created the function `FindTour` is called a predetermined number of times (`Runs`). `FindTour` performs a number of *trials* where in each trial it attempts to improve a chosen initial tour using the modified Lin-Kernighan edge exchange heuristics. Each time a better tour is found, the tour is recorded, and the candidates are reordered with the function `AdjustCandidateSet`. Precedence is given to edges that are common to the two currently best tours. The candidate set is extended with those tour edges that are not present in the current set. The original candidate set is re-established at exit from `FindTour`.

```
double FindTour() {
    int Trial;
    double BetterCost = DBL_MAX, Cost;

    for (Trial = 1; Trial <= Trials; Trial++) {
        ChooseInitialTour();
        Cost = LinKernighan();
        if (Cost < BetterCost) {
            RecordBetterTour();
            BetterCost = Cost
            AdjustCandidateSet();
        }
    }
    ResetCandidateSet();
    return BetterCost;
}
```

The following function, `LinKernighan`, seeks to improve a tour by sequential and nonsequential edge exchanges.

```
double LinKernighan() {
    Node *t1, *t2;
    int X2, Failures;
    long G, Gain;
    double Cost = 0;

    ForAllNodes(t1)
        Cost += C(t1,SUC(t1));
    do {
        Failures = 0;
        ForallNodes(t1, Failures < Dimension) {
            for (X2 = 1; X2 <= 2; X2++) {
                t2 = X2 == 1 ? PRED(t1) : SUC(t1);
                if (InBetterTour(t1,t2))
                    continue;
                G = C(t1,t2);
                while (t2 = BestMove(t1, t2, &G, &Gain)) {
                    if (Gain > 0) {
                        Cost -= Gain;
                        StoreTour();
                        Failures = 0;
                        goto Next_t1;
                    }
                }
                Failures++;
                RestoreTour();
            }
        Next_t1: ;
        }
        if ((Gain = Gain23()) > 0) {
            Cost -= Gain;
            StoreTour();
        }
    } while (Gain > 0);
}
```

First, the function computes the cost of the initial tour. Then, as long as improvements may be achieved, attempts are made to find improvements using sequential 5-opt moves (with `BestMove`), or when not possible, using nonsequential moves (with `Gain23`). For each sequential exchange, a basis edge (`t1`,`t2`) is found by selecting `t1` from the set of nodes and then selecting `t2` as one of `t1`'s two neighboring nodes in the tour. If (`t1`,`t2`) is an edge of the trial's best tour (`BetterTour`), then it is not used as a basis edge.

The function `BestMove` is sketched below. The function `BestMove` makes sequential edge exchanges. If possible, it makes an r-opt move ($r \leq 5$) that improves the tour. Otherwise, it makes the most promising 5-opt move that fulfils the positive gain criterion.

```
Node *BestMove(Node *t1, Node *t2, long *G0, long *Gain) {
    Node *t3, *t4, *t5, *t6, *t7, *t8, *t9, *t10;
    Node *T3, *T4, *T5, *T6, *T7, *T8, *T9, *T10 = 0;
    long G1, G2, G3, G4, G5, G6, G7, G8, BestG8 = LONG_MIN;
    int X4, X6, X8, X10;

    *Gain = 0; Reversed = SUC(t1) != t2;
    ForAllCandidates(t3, t2->CandidateSet) {
        if (t3 == PRED(t2) || t3 == SUC(t2) ||
            (G1 = *G0 - C(t2,t3)) <= 0)
            continue;
        for (X4 = 1; X4 <= 2; X4++) {
            t4 = X4 == 1 ? PRED(t3) : SUC(t3);
            G2 = G1 + C(t3,t4);
            if (X4 == 1 && (*Gain = G2 - C(t4,t1)) > 0) {
                Make2OptMove(t1, t2, t3, t4);
                return t4;
            }
            ForAllCandidates(t5, t4->CandidateSet) {
                if (t5 == PRED(t4) || t5 == SUC(t4) ||
                    (G3 = G2 - C(t4,t5)) <= 0)
                    continue;
                for (X6 = 1; X6 <= 2; X6++) {
                    Determine (T3,T4,T5,T6,T7,T8,T9,T10) =
                              (t3,t4,t5,t6,t7,t8,t9,t10)
                    such that
                       G8 = *G0 - C(t2,T3) + C(T3,T4)
                                - C(T4,T5) + C(T5,T6)
                                - C(T6,T7) + C(T7,T8)
                                - C(T8,T9) + C(T9,T10)
                    is maximum (= BestG8), and (T9,T10) has
                    not previously been included; if during
                    this process a legal move with *Gain > 0
                    is found, then make the move and exit
                    from BestMove immediately;
                }
            }
        }
    }
    *Gain = 0;
    if (T10 == 0) return 0;
    Make5OptMove(t1,t2,T3,T4,T5,T6,T7,T8,T9,T10);
    *G0 = BestG8;
    return T10;
}
```

Only the first part of the function (the 2-opt part) is given in some detail. The rest of the function follows the same pattern. The tour is as a circular list. The flag `Reversed` is used to indicate the reversal of a tour.

To prevent an infinite chain of moves the last edge to be deleted in a 5-opt move, (`T9,T10`), must not previously have been included in the chain.

A more detailed description of data structures and other implementation issues may be found in the following section.

5. Implementation

The modified Lin-Kernighan algorithm has been implemented in the programming language C. The software, approximately 4000 lines of code, is entirely written in ANSI C and portable across a number of computer platforms and C compilers. The following subsections describe the user interface and the most central techniques employed in the implementation.

5.1 User interface

The software includes code both for reading problem instances and for printing solutions.

Input is given in two separate files:

(1) the *problem file* and
(2) the *parameter file*.

The *problem file* contains a specification of the problem instance to be solved. The file format is the same as used in TSPLIB [38], a publicly available library of problem instances of the TSP.

The current version of the software allows specification of symmetric, asymmetric, as well as Hamiltonian tour problems.

Distances (costs, weights) may be given either explicitly in matrix form (in a full or triangular matrix), or implicitly by associating a 2- or 3-dimensional coordinate with each node. In the latter case distances may be computed by either a Euclidean, Manhattan, maximum, geographical or pseudo-Euclidean distance function. See [38] for details. At present, all distances must be integral.

Problems may be specified on a complete or sparse graph, and there is an option to require that certain edges must appear in the solution of the problem.

The *parameter file* contains control parameters for the solution process. The solution process is typically carried out using default values for the parameters. The default values have proven to be adequate in many applications. Actually, almost all computational tests reported in this paper have been made using these default settings. The only information that cannot be left out is the name of the problem file.

The format is as follows:

PROBLEM_FILE = <*string*>
Specifies the name of the problem file.

Additional control information may be supplied in the following format:

RUNS = <*integer*>
The total number of runs.
Default: 10.

MAX_TRIALS = <*integer*>
The maximum number of trials in each run.
Default: number of nodes (DIMENSION, given in the problem file).

TOUR_FILE = <*string*>
Specifies the name of a file to which the best tour is to be written.

OPTIMUM = <*real*>
Known optimal tour length. A run will be terminated as soon as a tour length less than or equal to optimum is achieved.
Default: DBL_MAX.

MAX_CANDIDATES = <*integer*> { SYMMETRIC }
The maximum number of candidate edges to be associated with each node.
The integer may be followed by the keyword SYMMETRIC, signifying that the candidate set is to be complemented such that every candidate edge is associated with both its two end nodes.
Default: 5.

ASCENT_CANDIDATES = <*integer*>
The number of candidate edges to be associated with each node during the ascent.
The candidate set is complemented such that every candidate edge is associated with both its two end nodes.
Default: 50.

EXCESS = <*integer*>
The maximum α-value allowed for any candidate edge is set to EXCESS times the absolute value of the lower bound of a solution tour (determined by the ascent).
Default: 1.0/DIMENSION.

INITIAL_PERIOD = <*integer*>
The length of the first period in the ascent.
Default: DIMENSION/2 (but at least 100).

INITIAL_STEP_SIZE = <*integer*>
The initial step size used in the ascent.
Default: 1.

PI_FILE = <*string*>
Specifies the name of a file to which penalties (π-values determined by the ascent) is to be written. If the file already exits, the penalties are read from the file, and the ascent is skipped.

PRECISION = <*integer*>
The internal precision in the representation of transformed distances:
d_{ij} = PRECISION$*c_{ij} + \pi_i + \pi_j$, where d_{ij}, c_{ij}, π_i and π_j are all integral.
Default: 100 (which corresponds to 2 decimal places).

SEED = <integer>
Specifies the initial seed for random number generation.
Default: 1.

SUBGRADIENT: [YES | NO]
Specifies whether the π-values should be determined by subgradient optimization.
Default: YES.

TRACE_LEVEL = <*integer*>
Specifies the level of detail of the output given during the solution process.
The value 0 signifies a minimum amount of output. The higher the value is the more information is given.
Default: 1.

During the solution process information about the progress being made is written to standard output. The user may control the level of detail of this information (by the value of the TRACE_LEVEL parameter).

Before the program terminates, a summary of key statistics is written to standard output, and, if specified by the TOUR_FILE parameter, the best tour found is written to a file (in TSPLIB format).

The user interface is somewhat primitive, but it is convenient for many applications. It is simple and requires no programming in C by the user. However, the current implementation is modular, and an alternative user interface may be implemented by rewriting a few modules. A new user interface might, for example, enable graphical animation of the solution process.

5.2 Representation of tours and moves

The representation of tours is a central implementation issue. The data structure chosen may have great impact on the run time efficiency. It is obvious that the major bottleneck of the algorithm is the search for possible moves (edge exchanges) and the execution of such moves on a tour. Therefore, special care should be taken to choose a data structure that allows fast execution of these operations.

The data structure should support the following primitive operations:

(1) find the predecessor of a node in the tour with respect to a chosen orientation (PRED);

(2) find the successor of a node in the tour with respect to a chosen orientation (SUC);

(3) determine whether a given node is between two other nodes in the tour with respect to a chosen orientation (BETWEEN);

(4) make a move;

(5) undo a sequence of tentative moves.

The necessity of the first three operations stems from the need to determine whether it is possible to 'close' the tour (see Figures 3.5-3.7). The last two operations are necessary for keeping the tour up to date.

In the modified Lin-Kernighan algorithm a move consists of a sequence of basic moves, where each basic move is a 5-opt move (k-opt moves with $k \leq 4$ are made in case an improvement of the tour is possible).

In order simplify tour updating, the following fact may be used: Any r-opt move ($r \geq 2$) is equivalent to a finite sequence of 2-opt moves [16, 39]. In the case of 5-opt moves it can be shown that any 5-opt move is equivalent to a sequence of at most five 2-opt moves. Any 4-opt move as well as any 3-opt move is equivalent to a sequence of at most three 2-opt moves.

This is exploited as follows. Any move is executed as a sequence of one or more 2-opt moves. During move execution, all 2-opt moves are recorded in a stack. A bad move is undone by unstacking the 2-opt moves and making the inverse 2-opt moves in this reversed sequence.

Thus, efficient execution of 2-opt moves is needed. A 2-opt move, also called a *swap*, consists of moving two edges from the current tour and reconnecting the resulting two paths in the best possible way (see Figure 2.1). This operation is seen to reverse one of the two paths. If the tour is represented as an array of nodes, or as a doubly linked list of nodes, the reversal of the path takes time O(n).

It turns out that data structures exist that allow logarithmic time complexity to be achieved [13, 40, 41, 42, 43]. These data structures, however, should not be selected without further notice. The time overhead of the corresponding update algorithms is usually large, and, unless the problem is large, typically more than 1000 nodes, update algorithms based on these data structures are

outperformed by update algorithms based on the array and list structures. In addition, they are not simple to implement.

In the current implementation of the modified Lin-Kernighan algorithm a tour may be represented in two ways, either by a *doubly linked list*, or by a *two-level tree* [43]. The user can select one of these two representations. The doubly linked list is recommended for problems with fewer than 1000 nodes. For larger problems the two-level tree should be chosen.

When the doubly link list representation is used, each node of the problem is represented by a C structure as outlined below.

```
struct Node {
    unsigned long Id, Rank;
    struct Node *Pred, *Suc;
};
```

The variable `Id` is the identification number of the node (`1` ≤ `Id` ≤ `n`).

`Rank` gives the ordinal number of the node in the tour. It is used to quickly determine whether a given node is between two other nodes in the tour.

`Pred` and `Suc` point to the predecessor node and the successor node of the tour, respectively.

A 2-opt move is made by swapping `Pred` and `Suc` of each node of one of the two segments, and then reconnecting the segments by suitable settings of `Pred` and `Suc` of the segments' four end nodes. In addition, `Rank` is updated for nodes in the reversed segment.

The following small code fragment shows the implementation of a 2-opt move. Edges (`t1`,`t2`) and (`t3`,`t4`) are exchanged with edges (`t2`,`t3`) and (`t1`,`t4`) (see Figure 2.1).

```
R = t1->Rank; t1->Suc = 0; s2 = t3;
while (s1 = s2) {
    s2 = s1->Suc;
    s1->Suc = s1->Pred;
    s1->Pred = s2;
    s1->Rank = R--;
}
t3->Suc = t2; t2->Pred = t3;
t1->Pred = t4; t4->Suc = t1;
```

Any of the two segments defined by the 2-opt move may be reversed. The segment with the fewest number of nodes is therefore reversed in order to speed up computations. The number of nodes in a segment can be found in constant time from the `Rank`-values of its end nodes. In this way much run time can be spared. For an example problem with 1000 nodes the average number of nodes touched during reversal was about 50, whereas a random reversal would have touched 500 nodes, on the average. For random Euclidean instances, the length of the shorter segment seems to grow roughly as $n^{0.7}$ [44].

However, the worst-case time cost of a 2-opt move is still O(n), and the costs of tour manipulation grow to dominate overall running time as n increases.

A worst-case cost of O(√n) per 2-opt move may be achieved using a two-level tree representation. This is currently the fastest and most robust representation on large instances that might arise in practice. The idea is to divide the tour into roughly √n segments. Each segment is maintained as a doubly linked list of nodes (using pointers labeled `Pred` and `Suc`).

Each node is represented by a C structure as outlined below.

```
struct Node {
    unsigned long Id, Rank;
    struct Node *Pred, *Suc;
    struct Segment *Parent;
};
```

`Rank` gives the position of the node within the segment, so as to facilitate BETWEEN queries. `Parent` is a pointer the segment containing the node.

Each segment is represented by the following C structure.

```
struct Segment {
    unsigned long Rank;
    struct Segment *Pred, *Suc;
    struct Node *First, *Last;
    bit Reversed;
};
```

The segments are connected in a doubly linked list (using pointers labeled `Pred` and `Suc`), and each segment contains a sequence number, `Rank`, that represents its position in the list.

`First` and `Last` are pointers to the segment's two end nodes. `Reversed` is a reversal bit indicating whether the segment should be traversed in forward or reverse direction. Just switching this bit reverses the orientation of a segment.

All the query operations (PRED, SUC and BETWEEN) are performed in constant time (as in the list representation, albeit with slightly larger constants), whereas the move operations have a worst-case cost of $O(\sqrt{n})$ per move.

The implementation of the operations closely follows the suggestions given in [43]. See [43, pp. 444-446] for details.

5.3 Distance computations

A bottleneck in many applications is the computing of distances. For example, if Euclidean distances are used, a substantial part of run time may be spent in computing square roots.

If sufficient space is available, all distances may be computed once and stored in a matrix. However, for large problems, say more than 5000 nodes, this approach is usually not possible.

In the present implementation, distances are computed once and stored in a matrix, only if the problem is smaller than a specified maximum dimension. For larger problems, the following techniques are used to reduce run time.

(1) Each candidate edge including its length is associated to the node from which it emanates. A large fraction of edges considered during the solution process are candidate edges. Therefore, in many cases the length of an edge may be found by a simple search among the candidate edges associated with its end nodes.

(2) Computational cheap functions are used in calculating lower bounds for distances. For example, a lower bound for an Euclidean distance $\sqrt{(dx^2+dy^2)}$ may be quickly computed as the maximum of $|dx|$ and $|dy|$. Often a reasonable lower bound for a distance is sufficient for deciding that there is no point in computing the true distance. This may, for example, be used for quickly deciding that a tentative move cannot possibly lead to a tour improvement. If the current gain, plus a lower bound for the distance of a closing edge, is not positive, then the tour will not be improved by this move.

(3) The number of distance computations is reduced by the caching technique described in [45]. When a distance between two nodes has been computed the distance is stored in a hash table. The hash index is computed from the identification numbers of the two nodes. Next time the distance between the same two nodes is to be computed, the table is consulted to see whether the distance is still available. See [45] for details. The effect of using the caching

technique was measured in the solution of a 2392-node problem. Here optimum was found with about 70 times fewer ordinary distance calculations than without the technique, and the running time was more than halved.

5.4 Reduction of checkout time

When the algorithm has found a local optimum, time is spent to check that no further progress is possible. This time, called the *checkout time*, can be avoided if the same local optimum has been found before. There is no point in attempting to find further improvements - the situation has been previously been 'checked out'. The checkout time often constitutes a substantial part of the running time. Lin and Kernighan report checkout times that are typically 30 to 50 per cent of running time.

The modified algorithm reduces checkout time by using the following techniques.

(1) Moves in which the first edge (t_1,t_2) to be broken belongs to the currently best solution tour are not investigated.

(2) A hashing technique is used. A hash function maps tours to locations in a hash table. Each time a tour improvement has been found, the hash table is consulted to see whether the new tour happens to be local optimum found earlier. If this is the case, fruitless checkout time is avoided. This technique is described in detail in [46].

(3) The concept of the *don't look bit*, introduced by Bentley [44], is used. If for a given choice of t_1 the algorithm previously failed to find an improvement, and if t_1's tour neighbors have not changed since that time, then it is unlikely that an improving move can made if the algorithm again looks at t_1. This is exploited as follows. Each node has a *don't look bit*, which initially is 0. The bit for node t_1 is set to 1 whenever a search for an improving move with t_1 fails, and it is set to 0 whenever an improving move is made in which it is an end node of one of the its edges. In considering candidates for t_1 all nodes whose don't look bit is 1 are ignored. This is done in maintaining a queue of nodes whose bits are zero.

5.5 Speeding up the ascent

Subgradient optimization is used to determine a lower bound for the optimum. At each step a minimum 1-tree is computed. Since the number of steps may be large, it is important to speed up the computation of minimum 1-trees. For this purpose, the trees are computed in sparse graphs.

The first tree is computed in a complete graph. All remaining trees but the last are computed in a sparse subgraph determined by the α-measure. The sub-

graph consists of a specified number of α-nearest neighbor edges incident to each node.

Prim's algorithm [22] is used for computing minimum spanning trees. Therefore, to achieve a speed-up it is necessary to quickly find the shortest edge from a number of edges. In the current implementation a binary heap is used for this purpose.

The combination of these methods results in fast computation of minimum spanning trees, at least when the number of candidate edges allowed for each node is not too large. On the other hand, this number should not be so small that the lower bound computed by the ascent is not valid. In the present implementation, the number is 50 by default.

6. Computational results

The performance of an approximate algorithm such as the Lin-Kernighan algorithm can be evaluated in three ways:

(1) by *worst-case* analysis
(2) by *probabilistic* (or *average-case*) analysis
(3) by *empirical* analysis

The goal of *worst-case* analysis is to derive upper bounds for possible deviations from optimum; that is, to provide quality guarantees for results produced by the algorithm.

All known approximate algorithms for the TSP have rather poor worst-case behavior. Assume, for example, that the problems to be solved are metric (the triangle inequality holds). Then the approximate algorithm known to have the best worst-case behavior is the algorithm of Christofides [37]. This algorithm guarantees a tour length no more than 50% longer than optimum. For any r-opt algorithm, where $r \le n/4$ (n being the number of cities), problems may be constructed such that the error is almost 100% [11]. For non-metric problems it can proven that it is impossible to construct an algorithm of polynomial complexity which find tours whose length is bound by a constant multiple of the optimal tour length [47].

The purpose of the second method, *probabilistic* analysis, is to evaluate average behavior of the algorithms. For example, for an approximate TSP algorithm probability analysis can used be to estimate the expected error for large problem sizes.

The worst-case as well as the probability approach, however, have their drawbacks. The mathematics involved may be very complex and results achieved by these methods may often be of little use when solving *practical* instances of TSP. Statements concerning problems that almost certainly do not occur in practice (‘pathological’ problems, or problems with an ‘infinite’ number of cities) will often be irrelevant in connection with practical problem solving.

In this respect the third method, *empirical* analysis, seems more appropriate. Here the algorithm is executed on a number of test problems, and the results are evaluated, often in relation to optimal solutions. The test problems may be generated at random, or they may be constructed in a special way. If the test problems are representative for those problems the algorithm is supposed to solve, the computations are useful for evaluating the appropriateness of the algorithm.

The following section documents computational results of the modified Lin-Kernighan algorithm. The results include the qualitative performance and the run time efficiency of the current implementation. Run times are measured in seconds on a 300 MHz G3 Power Macintosh.

The performance of the implementation has been evaluated on the following spectrum of problems:

(1) Symmetric problems
(2) Asymmetric problems
(3) Hamiltonian cycle problems
(4) Pathological problems

Each problem has been solved by a number of independent *runs*. Each run consist of a series of *trials*, where in each trial a tour is determined by the modified Lin-Kernighan algorithm. The trials of a run are not independent, since edges belonging to the best tour of the current run are used to prune the search.

In the experiments the number of runs varies. In problems with less than 1000 cities the number of runs is 100. In larger problems the number of runs is 10.

The number of trials in each run is equal to the dimension of the problem (the number of cities). However, for problems where optimum is known, the current series of trials is stopped if the algorithm finds optimum.

6.1 Symmetric problems

TSPLIB [38] is a library, which is meant to provide researchers with a set of sample instances for the TSP (and related problems). TSPLIB is publicly available via FTP from `softlib.rice.edu` and contains problems from various sources and with various properties.

At present, instances of the following problem classes are available: symmetric traveling salesman problems, asymmetric traveling salesman problems, Hamiltonian cycle problems, sequential ordering problems, and capacitated vehicle routing problems. Information on the length of optimal tours, or lower and upper bounds for this length, is provided (if available).

More than 100 symmetric traveling salesman problems are included in the library, the largest being a problem with 85900 cities. The performance evaluation that follows is based on those problems for which the optimum is known. Today there are 100 problems of this type in the library, ranging from a problem with 14 cities to a problem with 7397 cities. The test results are reported in Table 6.1 and 6.2.

Table 6.1 displays the results from the subgradient optimization phase. The table gives the problem names along with the number of cities, the problem type, the optimal tour length, the lower bound, the gap between optimum and lower bound as a percentage of optimum, and the time in seconds used for subgradient optimization.

The problem type specifies how the distances are given. The entry MATRIX indicates that distances are given explicitly in matrix form (as full or triangular matrix). The other entry names refer to functions for computing the distances from city coordinates. The entries EUC_2D and CEIL_2D both indicates that distances are the 2-dimensional Euclidean distances (they differ in their rounding method). GEO indicates geographical distances on the Earth's surface, and ATT indicates a special 'pseudo-Euclidean' distance function. All distances are integer numbers. See [38] for details.

Name	Cities	Type	Optimum	Lower bound	Gap	Time
a280	280	EUC_2D	2579	2565.8	0.5	0.7
ali535	535	GEO	202310	201196.2	0.6	4.0
att48	48	ATT	10628	10602.1	0.2	0.1
att532	532	ATT	27686	27415.7	1.0	2.9
bayg29	29	GEO	1610	1608.0	0.1	0.0
bays29	29	GEO	2020	2013.3	0.3	0.0
berlin52	52	EUC_2D	7542	7542.0	*0.0	0.1
bier127	127	EUC_2D	118282	117430.6	0.7	0.3
brazil58	58	MATRIX	25395	25354.2	0.2	0.1
brg180	180	MATRIX	1950	1949.3	0.0	0.3
burma14	14	GEO	3323	3223.0	*0.0	0.0
ch130	130	EUC_2D	6110	6074.6	0.6	0.2
ch150	150	EUC_2D	6528	6486.6	0.6	0.3
d198	198	EUC_2D	15780	145672.9	7.6	0.4
d493	493	EUC_2D	35002	34822.4	0.5	2.5
d657	657	EUC_2D	48912	48447.6	0.9	5.1
d1291	1291	EUC_2D	50801	50196.8	1.2	19.0
d1655	1655	EUC_2D	62128	61453.3	1.1	47.0
dantzig42	42	MATRIX	699	697.0	0.3	0.0
dsj1000	1000	CEIL_2D	18659688	18339778.9	1.7	12.3
eil51	51	EUC_2D	426	422.4	0.8	0.1
eil76	76	EUC_2D	538	536.9	0.2	0.1
eil101	101	EUC_2D	629	627.3	0.3	0.2
fl417	417	EUC_2D	11861	11287.3	4.8	2.0
fl1400	1400	EUC_2D	20127	19531.9	3.0	36.5
fl1577	1577	EUC_2D	22249	21459.3	3.5	45.2
fnl4461	4461	EUC_2D	182566	181566.1	0.5	332.7
fri26	26	MATRIX	937	937.0	*0.0	0.0
gil262	262	EUC_2D	2378	2354.4	1.0	0.6
gr17	17	MATRIX	2085	2085.0	*0.0	0.0
gr21	21	MATRIX	2707	2707.0	*0.0	0.0
gr24	24	MATRIX	1272	1272.0	*0.0	0.0
gr48	48	MATRIX	5046	4959.0	1.7	0.1
gr96	96	GEO	55209	54569.5	1.2	0.2
gr120	120	MATRIX	6942	6909.9	0.5	0.2
gr137	137	GEO	69853	69113.1	1.1	0.4
gr202	202	GEO	40160	40054.9	0.3	0.4
gr229	229	GEO	134602	133294.7	1.0	0.7
gr431	431	GEO	171414	170225.9	0.7	2.0
gr666	666	GEO	294358	292479.3	0.6	4.8
hk48	48	MATRIX	11461	11444.0	0.1	0.0
kroA100	100	EUC_2D	21282	20936.5	1.6	0.1
kroB100	100	EUC_2D	22141	21831.7	1.4	0.1
kroC100	100	EUC_2D	20749	20472.5	1.3	0.2
kroD100	100	EUC_2D	21294	21141.5	0.7	0.2
kroE100	100	EUC_2D	22068	21799.4	1.2	0.2
kroA150	150	EUC_2D	26524	26293.2	0.9	0.3

Table 6.1 Determination of lower bounds (Part I)

Name	Cities	Type	Optimum	Lower bound	Gap	Time
kroB150	150	EUC_2D	26130	25732.4	1.5	0.3
kroA200	200	EUC_2D	29368	29056.8	1.1	0.7
kroB200	200	EUC_2D	29437	29163.8	0.9	0.4
lin105	105	EUC_2D	14379	14370.5	0.1	0.2
lin318	318	EUC_2D	42029	41881.1	0.4	1.5
linhp318	318	EUC_2D	41345	41224.3	0.3	1.2
nrw1379	1379	EUC_2D	56638	56393.2	0.4	23.3
p654	654	EUC_2D	34643	33218.1	4.1	4.5
pa561	561	MATRIX	2763	2738.4	0.9	3.2
pcb442	442	EUC_2D	50778	50465.0	0.6	2.0
pcb1173	1173	EUC_2D	56892	56349.7	1.0	15.7
pcb3038	3038	EUC_2D	137694	136582.0	0.8	139.8
pla7397	7397	CEIL_2D	23260728	23113655.4	0.6	1065.6
pr76	76	EUC_2D	108159	105050.6	2.9	0.1
pr107	107	EUC_2D	44303	39991.5	9.7	0.2
pr124	124	EUC_2D	59030	58060.6	1.6	0.2
pr136	136	EUC_2D	96772	95859.2	0.9	0.3
pr144	144	EUC_2D	58537	57875.7	1.1	0.3
pr152	152	EUC_2D	73682	72166.3	2.1	0.5
pr226	226	EUC_2D	80369	79447.8	1.1	0.6
pr264	264	EUC_2D	49135	46756.3	4.8	0.6
pr299	299	EUC_2D	48191	47378.5	1.7	0.9
pr439	439	EUC_2D	107217	105816.3	1.3	2.0
pr1002	1002	EUC_2D	259045	256726.9	0.9	15.6
pr2392	2392	EUC_2D	378032	373488.5	1.2	84.6
rat99	99	EUC_2D	1211	1206.0	0.4	0.2
rat195	195	EUC_2D	2323	2292.0	1.3	0.3
rat575	575	EUC_2D	6773	6723.4	0.7	3.2
rat783	783	EUC_2D	8806	8772.1	0.4	6.5
rd100	100	EUC_2D	7910	7897.1	0.2	0.1
rd400	400	EUC_2D	15281	15155.9	0.8	1.7
rl1304	1304	EUC_2D	252948	249079.2	1.5	19.9
rl1323	1323	EUC_2D	270199	265810.4	1.6	20.8
rl1889	1889	EUC_2D	316536	311305.0	1.7	49.6
si175	175	MATRIX	21407	21373.6	0.2	0.3
si535	535	MATRIX	48450	48339.9	0.2	2.8
si1032	1032	MATRIX	92650	92434.4	0.2	12.6
st70	70	EUC_2D	675	670.9	0.6	0.1
swiss42	42	MATRIX	1273	1271.8	0.1	0.0
ts225	225	EUC_2D	126643	115604.6	8.7	0.5
tsp225	225	EUC_2D	3919	3880.3	1.0	0.5
u159	159	EUC_2D	42080	41925.0	0.4	0.3
u574	574	EUC_2D	36905	36710.3	0.5	3.3
u724	724	EUC_2D	41910	41648.9	0.6	5.2
u1060	1060	EUC_2D	224094	222626.4	0.7	12.2
u1432	1432	EUC_2D	152970	152509.2	0.3	25.7
u1817	1817	EUC_2D	57201	56681.7	0.9	42.9

Table 6.1 Determination of lower bounds (Part II)

Name	Cities	Type	Optimum	Lower bound	Gap	Time
u2152	2152	EUC_2D	64253	63848.1	0.6	65.2
u2319	2319	EUC_2D	234256	234152.0	0.0	86.2
ulysses16	16	GEO	6859	6859.0	*0.0	0.0
ulysses22	22	GEO	7013	7013.0	*0.0	0.0
vm1084	1084	EUC_2D	239297	236144.7	1.3	12.7
vm1748	1748	EUC_2D	336556	332049.8	1.3	40.6

Table 6.1 Determination of lower bounds (Part III)

The average gap between optimum and lower bound is 1.1%. For some of the small problems (berlin52, burma14, fri26, gr17, gr21, gr24, ulysses16 and ulysses22) the gap is zero, that is, optima are determined by subgradient optimization (marked with a *).

Table 6.2 documents the performance of the search heuristics. The table gives the problem names along with the ratio of runs succeeding in finding the optimal solution, the minimum and average number of trials in a run, the minimum and average gap between the length of the best tour obtained and optimum as a percentage of optimum, and the minimum and average time in seconds per run.

For example, for the problem att532 of Padberg and Rinaldi [19] the optimal solution was determined in 98 runs out of 100. The minimum number of trials made to find optimum was 1. The average number of trials in a run was 66.2 (a run is stopped if the optimal solution is found, or the number of trials made equals the number of cities). The average gap between the length of the best tour obtained and optimum as a percentage of optimum was 0.001%. The maximum gap was 0.072%. Finally, the minimum and average CPU time used in a run was 0.2 and 3.6 seconds, respectively.

All instances were solved to optimality with the default parameter settings.

Name	Success	$Trials_{min}$	$Trials_{avg}$	Gap_{avg}	Gap_{max}	$Time_{min}$	$Time_{avg}$
a280	100/100	1	1.0	0.000	0.000	0.0	0.1
ali535	100/100	1	8.0	0.000	0.000	0.2	0.7
att48	100/100	1	1.0	0.000	0.000	0.0	0.0
att532	98/100	1	66.2	0.001	0.072	0.2	3.6
bayg29	100/100	1	1.0	0.000	0.000	0.0	0.0
bays29	100/100	1	1.0	0.000	0.000	0.0	0.0
berlin52	*1/0	1	1.0	0.000	0.000	0.0	0.0
bier127	100/100	1	1.1	0.000	0.000	0.0	0.0
brazil58	100/100	1	1.0	0.000	0.000	0.0	0.0
brg180	100/100	1	3.3	0.000	0.000	0.0	0.0
burma14	*1/0	1	1.0	0.000	0.000	0.0	0.0
ch130	100/100	1	3.0	0.000	0.000	0.0	0.1
ch150	62/100	1	62.9	0.023	0.077	0.0	0.4
d198	100/100	1	13.6	0.000	0.000	0.2	1.0
d493	100/100	1	45.8	0.000	0.000	0.2	3.2
d657	100/100	1	85.8	0.000	0.000	0.3	3.3
d1291	8/10	152	584.2	0.033	0.167	23.1	59.8
d1655	10/10	98	494.6	0.000	0.000	10.5	41.1
dantzig42	100/100	1	1.0	0.000	0.000	0.0	0.0
dsj1000	7/10	90	514.1	0.035	0.116	13.1	55.1
eil51	100/100	1	1.0	0.000	0.000	0.0	0.0
eil76	100/100	1	1.0	0.000	0.000	0.0	0.0
eil101	100/100	1	1.0	0.000	0.000	0.0	0.0
fl417	88/100	1	64.2	0.052	0.430	1.2	12.4
fl1400	1/10	13	1261.3	0.162	0.199	13.1	583.3
fl1577	2/10	134	1350.1	0.046	0.063	120.2	1097.5
fnl4461	6/10	665	2767.3	0.001	0.003	284.1	1097.3
fri26	100/100	1	1.0	0.000	0.000	0.0	0.0
gil262	100/100	1	13.3	0.000	0.000	0.1	0.4
gr17	*1/0	1	1.0	0.000	0.000	0.0	0.0
gr21	*1/0	1	1.0	0.000	0.000	0.0	0.0
gr24	100/100	1	1.0	0.000	0.000	0.0	0.0
gr48	100/100	1	1.0	0.000	0.000	0.0	0.0
gr96	100/100	1	10.2	0.000	0.000	0.0	0.1
gr120	100/100	1	1.0	0.000	0.000	0.0	0.0
gr137	100/100	1	1.0	0.000	0.000	0.0	0.0
gr202	100/100	1	1.0	0.000	0.000	0.0	0.1
gr229	12/100	6	216.6	0.009	0.010	0.2	1.7
gr431	17/100	81	401.4	0.053	0.077	4.4	13.1
gr666	30/100	25	531.9	0.026	0.040	2.0	18.8
hk48	100/100	1	1.0	0.000	0.000	0.0	0.0
kroA100	100/100	1	1.0	0.000	0.000	0.0	0.0
kroB100	100/100	1	1.4	0.000	0.000	0.0	0.1
kroC100	100/100	1	1.0	0.000	0.000	0.0	0.0
kroD100	100/100	1	1.3	0.000	0.000	0.0	0.0
kroE100	99/100	1	10.7	0.002	0.172	0.0	0.2
kroA150	100/100	1	1.2	0.000	0.000	0.0	0.1

Table 6.2 Determination of solutions (Part I)

Name	Success	$Trials_{min}$	$Trials_{avg}$	Gap_{avg}	Gap_{max}	$Time_{min}$	$Time_{avg}$
kroB150	55/100	1	97,7	0.003	0.008	0.1	0.8
kroA200	100/100	1	2.1	0.000	0.000	0.1	0.2
kroB200	100/100	1	2.0	0.000	0.000	0.0	0.1
lin105	100/100	1	1.0	0.000	0.000	0.0	0.0
lin318	71/100	1	154.1	0.076	0.271	0.1	2.0
linhp318	100/100	1	2.4	0.000	0.000	0.0	0.1
nrw1379	3/10	414	1148.5	0.006	0.009	20.2	69.3
p654	100/100	1	46.6	0.000	0.000	1.1	9.7
pa561	99/100	1	40.8	0.001	0.072	0.2	3.5
pcb442	93/100	1	102.7	0.001	0.014	0.1	4.0
pcb1173	8/10	2	475.2	0.002	0.009	0.6	14.6
pcb3038	9/10	121	1084.7	0.000	0.004	39.1	323.7
pla7397	7/10	1739	4588.4	0.001	0.004	4507.9	13022.0
pr76	100/100	1	1.0	0.000	0.000	0.0	0.1
pr107	100/100	1	1.0	0.000	0.000	0.0	0.0
pr124	100/100	1	1.4	0.000	0.000	0.0	0.1
pr136	100/100	1	1.0	0.000	0.000	0.1	0.2
pr144	100/100	1	1.0	0.000	0.000	0.1	0.1
pr152	100/100	1	3.4	0.000	0.000	0.1	0.1
pr226	100/100	1	1.0	0.000	0.000	0.1	0.1
pr264	100/100	1	7.5	0.000	0.000	0.2	0.5
pr299	100/100	1	4.5	0.000	0.000	0.3	0.8
pr439	98/100	1	72.8	0.001	0.041	0.1	1.6
pr1002	10/10	38	215.1	0.000	0.000	1.0	3.4
pr2392	10/10	37	396.6	0.000	0.000	8.7	54.5
rat99	100/100	1	1.0	0.000	0.000	0.0	0.0
rat195	100/100	1	3.5	0.000	0.000	0.1	0.4
rat575	77/100	2	290.6	0.004	0.030	0.4	8.2
rat783	100/100	1	6.6	0.000	0.000	0.1	0.3
rd100	100/100	1	1.1	0.000	0.000	0.0	0.0
rd400	99/100	1	51.0	0.000	0.020	0.1	1.1
rl1304	8/10	14	840.0	0.019	0.161	1.1	35.8
rl1323	1/10	244	1215.1	0.018	0.048	10.9	51.6
rl1889	4/10	1	1418.1	0.002	0.004	1.2	113.8
si175	100/100	1	6.8	0.000	0.000	0.1	0.2
si535	33/100	70	460.8	0.006	0.017	5.2	30.0
si1032	2/10	49	844.6	0.057	0.071	3.4	20.1
st70	100/100	1	1.0	0.000	0.000	0.0	0.0
swiss42	100/100	1	1.0	0.000	0.000	0.0	0.0
ts225	100/100	1	1.0	0.000	0.000	0.1	0.1
tsp225	100/100	1	5.8	0.000	0.000	0.1	0.3
u159	100/100	1	1.0	0.000	0.000	0.0	0.0
u574	91/100	1	111.4	0.007	0.081	0.2	3.2
u724	98/100	4	162.5	0.000	0.005	0.6	6.8
u1060	9/10	2	305.9	0.000	0.003	1.6	38.0
u1432	10/10	3	59.9	0.000	0.000	0.8	8.0
u1817	2/10	905	1707.8	0.078	0.124	199.6	252.9

Table 6.2 Determination of solutions (Part II)

Name	Success	$Trials_{min}$	$Trials_{avg}$	Gap_{avg}	Gap_{max}	$Time_{min}$	$Time_{avg}$
u2152	5/10	491	1706.5	0.029	0.089	85.0	274.2
u2319	10/10	1	3.2	0.000	0.000	0.5	2.6
ulysses16	*1/0	1	1.0	0.000	0.000	0.0	0.0
ulysses22	*1/0	1	1.0	0.000	0.000	0.0	0.0
vm1084	7/10	8	425.2	0.007	0.022	1.9	28.6
vm1748	4/10	65	1269.6	0.023	0.054	7.3	1016.1

Table 6.2 Determination of solutions (Part III)

In addition to these problems, TSPLIB contains 11 symmetric problems for which no optimum solutions are known today. The dimension of these problems varies from 2103 to 85900 cities.

Table 6.3 lists for each of these problems the currently best known lower and upper bound (published in TSPLIB, October 1997).

Name	Cities	Type	Lower bound	Upper bound
brd14051	14051	EUC_2D	469272	469445
d2103	2103	EUC_2D	80099	80450
d15112	15112	EUC_2D	1572810	1573152
d18512	18512	EUC_2D	645075	645300
fl3795	3795	EUC_2D	28724	28772
pla33810	33810	CEIL_2D	65960739	66116530
pla85900	85900	CEIL_2D	142244225	142482068
rl5915	5915	EUC_2D	565277	565530
rl5934	5934	EUC_2D	555579	556045
rl11849	11849	EUC_2D	922859	923368
usa13509	13509	EUC_2D	19981013	19982889

Table 6.3 Problems in TSPLIB with unknown optimal solutions

When the new algorithm was executed on these problems, it found tour lengths equal to the best known upper bounds for 4 of the problems (d2103, fl3795, rl5915 and rl5934).

However, for the remaining 7 problems, the algorithm was able find tours *shorter* than the best known upper bounds. These new upper bounds are listed in Table 6.4.

Name	New upper bound
brd14051	469395
d15112	1573089
d18512	645250
pla33810	66060236
pla85900	142416327
rl11849	923307
usa13509	19982859

Table 6.4 Improved upper bounds

The new upper bound for the largest of these problems, pla85900, was found using two weeks of CPU time.

It is difficult to predict the running time needed to solve a given problem with the algorithm. As can be seen from Table 6.2, the size alone is not enough. One problem may require much more running time than a larger problem.

However, some guidelines may be given. Random problems may be used to provide estimates of running times. By solving randomly generated problems of different dimensions and measuring the time used to solve these problems, it is possible to get an idea of how running time grows as a function of problem dimension.

An algorithm for randomly generating traveling salesman problem with known optimal tours, described by Arthur and Frendewey [48], was used for this purpose. Figure 6.1 and 6.2 show total running times (in seconds) for solving symmetric with problems generated by this algorithm. The dimension varies from 50 to 1000 cities. The following parameter values have been chosen: $\rho = 0.1$, $\sigma = 0.25$ (both suggested by Arthur and Frendewey), and $R = n$, where n is the dimension of the problem. See [48] for details.

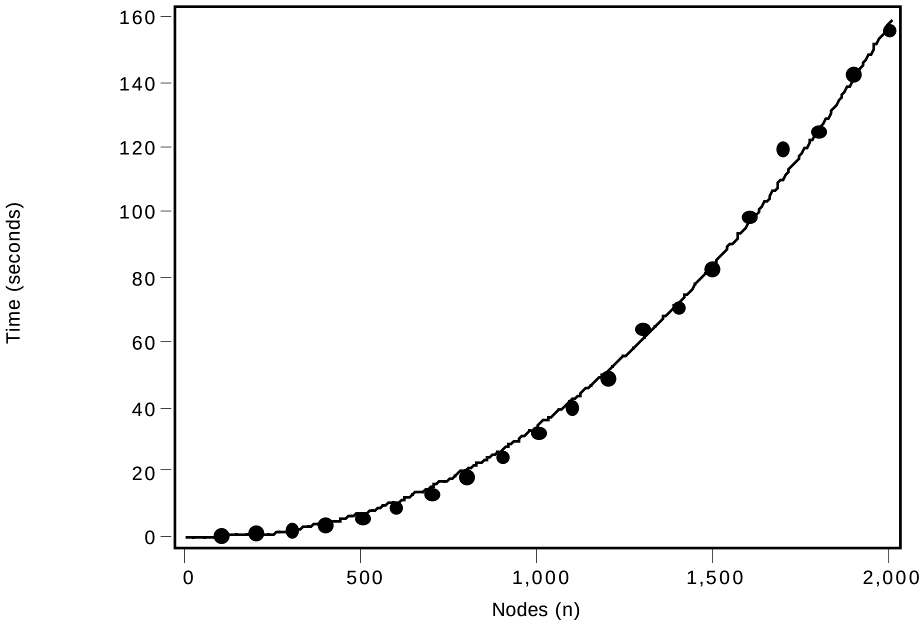

Figure 6.1 Run time as a function of problem dimension (non-metric problems)

The problems used to produce Figure 6.1 are not required to satisfy the triangle inequality. These problems are very simple for the algorithm. For all problems, optimum was found in only one trial. The curve depicts the function $f(n) = 8.6e^{-6}*n^{2.2}$ (correlation coefficient: 0.997).

Figure 6.2 depicts total running time as a function of dimension for randomly generated problems satisfying the triangle inequality. These problems seem to be harder to solve (probably due to a smaller diversity in the distances). However, the optimum for these problems was also found in one trial only. The curve depicts the function $f(n) = 3.2e^{-5}* n^{2.2}$ (correlation coefficient : 0.944).

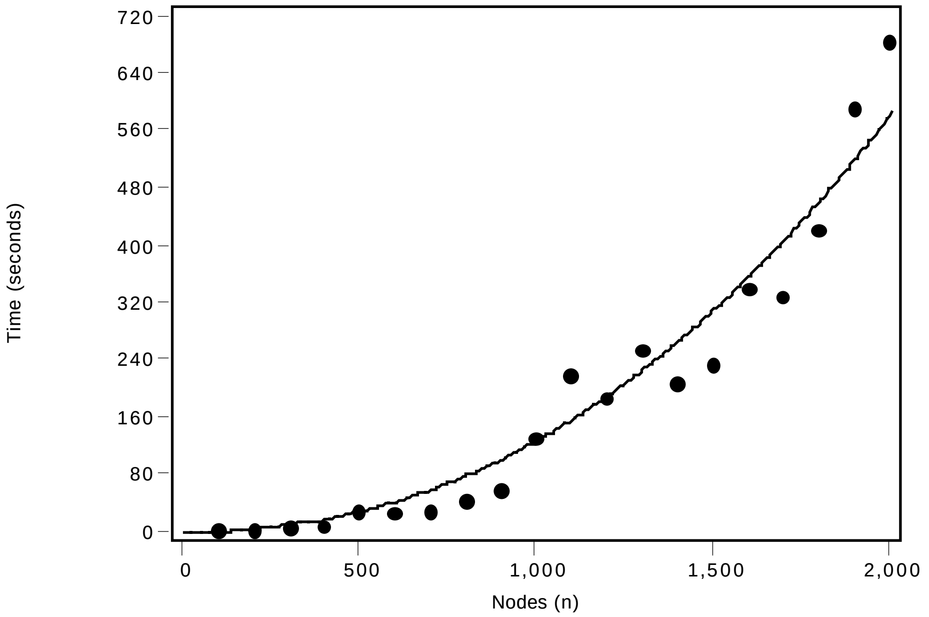

Figure 6.2 Run time as a function of problem dimension (metric problems)

These results indicate that the average running time of the algorithm is approximately $O(n^{2.2})$.

Another method often used in studying the average performance of TSP heuristics is to solve problem instances consisting of random points within a rectangle under the Euclidean metric. The instances are solved for increasing values of n and compared to the theoretical value for the *expected* length of an optimal tour (L_{opt}). A well-known formula is $L_{opt}(n,A) = K\sqrt{n}\sqrt{A}$ when n cities are distributed uniformly randomly over a rectangular area of A units [49]. That is, the ratio of the optimal tour length to $\sqrt{n}\sqrt{A}$ approaches a constant K for $N \to \infty$. Experiments of Johnson, McGeoch and Rothenberg [50] suggest that K is approximated by 0.7124.

Figure 6.3 shows the results obtained when the modified Lin-Kernighan algorithm was used to solve such problems on a 10^4x10^4 square for n ranging from 500 to 5000 and n increasing by 500. The figure depicts the tour length divided by $\sqrt{n}*10^4$. These results are consistent with the estimate $K \approx 0.7124$ for large n.

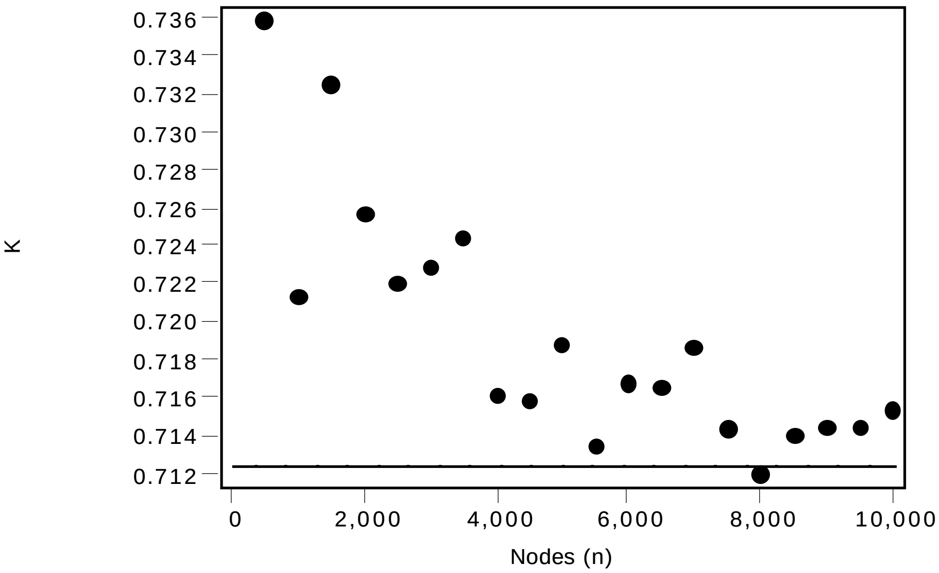

Figure 6.3 Solutions of random Euclidean problems on a square

Figure 6.4 shows the total running time in seconds to find the best (local optimal) tour as a function of problem dimension. The curve depicts the function $f(n) = 2.9e^{-5} * n^{2.2}$ (with correlation coefficient 0.968).

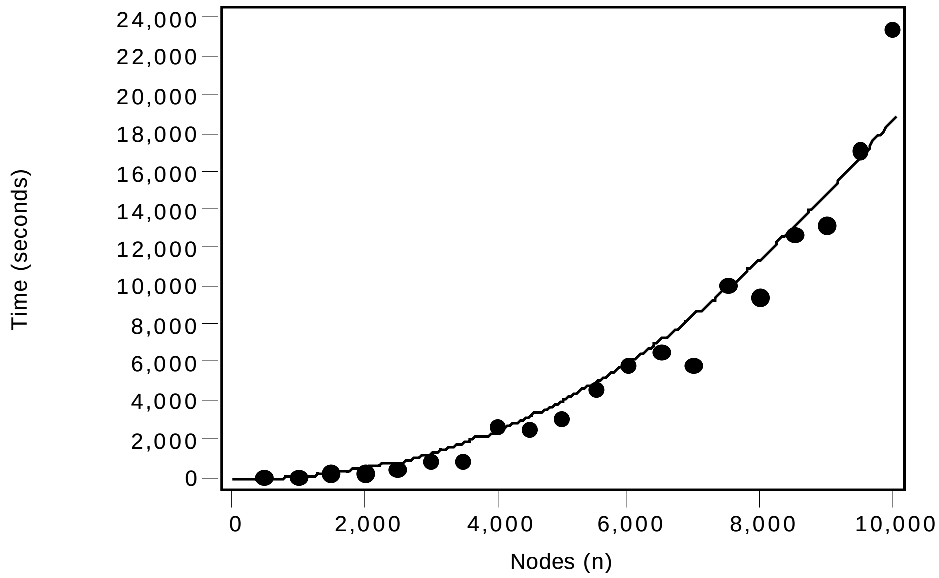

Figure 6.4 Run time as a function of problem dimension

6.2 Asymmetric problems

The implemented algorithm is primarily intended for solving symmetric TSPs. However, any asymmetric problem may be transformed into a symmetric problem and therefore be solved by the algorithm.

The transformation method of Jonker and Volgenant [51] transforms a asymmetric problem with n nodes into a problem 2n nodes. Let $C = (c_{ij})$ denote the nxn cost matrix of the asymmetric problem. Then let $C' = (c'_{ij})$ be the 2nx2n symmetric matrix computed as follows:

$$c'_{n+i,j} = c'_{j,n+i} = c_{i,j} \quad \text{for } i = 1, 2, ..., n,\ j = 1, 2, ..., n, \text{ and } i \neq j$$

$$c'_{n+i,i} = c'_{i,n+i} = -M \quad \text{for } i = 1, 2, ..., n$$

$$c'_{i,j} = M \quad \text{otherwise}$$

where M is a sufficiently large number, e.g., $M = \max(c_{ij})$.

It is easy to prove that any optimal solution of the new symmetric problem corresponds to an optimal solution of the original asymmetric problem.

An obvious disadvantage of the transformation is that it doubles the size of the problem. Therefore, in practice it is more advantageous to use algorithms dedicated for solving asymmetric problems. However, as can been seen from Table 6.5 the performance of the modified Lin-Kernighan algorithm for asymmetric problems is quite impressive. The optimum was obtained for all asymmetric problems of TSPLIB. The largest of these problems, rbg443, has 443 nodes (thus, the transformed problem has 886 nodes).

The problems prefixed with `ft` have size equal to their suffix number plus 1.

Name	Success	$Trials_{min}$	$Trials_{avg}$	Gap_{avg}	Gap_{max}	$Time_{min}$	$Time_{avg}$
br17	100/100	1	1.0	0.000	0.000	0.0	0.0
ft53	100/100	1	8.0	0.000	0.000	0.0	0.0
ft70	100/100	1	1.6	0.000	0.000	0.0	0.0
ftv33	100/100	1	1.0	0.000	0.000	0.0	0.0
ftv35	47/100	1	43.6	0.072	0.136	0.0	0.1
ftv38	53/100	1	40.3	0.061	0.131	0.0	0.1
ftv44	100/100	1	2.8	0.000	0.000	0.0	0.0
ftv47	100/100	1	1.5	0.000	0.000	0.0	0.0
ftv55	100/100	1	3.5	0.000	0.000	0.0	0.0
ftv64	100/100	1	3.6	0.000	0.000	0.0	0.0
ftv70	100/100	1	3.6	0.000	0.000	0.0	0.0
ftv170	88/100	1	119.7	0.039	0.327	0.0	1.0
kro124p	95/100	1	24.6	0.002	0.030	0.0	0.1
p43	21/100	1	72.2	0.014	0.018	0.0	0.5
rbg323	97/100	17	116.3	0.018	0.679	1.9	8.5
rbg358	99/100	19	96.8	0.060	6.019	2.5	8.3
rbg403	100/100	19	87.5	0.000	0.000	2.6	11.3
rbg443	100/100	18	105.2	0.000	0.000	2.5	12.6
ry48p	99/100	1	9.8	0.002	0.166	0.0	0.0

Table 6.5 Performance for asymmetric problems

6.3 Hamiltonian cycle problems

The Hamiltonian cycle problem is the problem of deciding if a given undirected graph contains a cycle. The problem can be answered by solving a symmetric TSP in the complete graph where all edges of G have cost 0 and all other edges have a positive cost. Then G contains a Hamiltonian cycle if and only if an optimal tour has cost 0.

At present TSPLIB includes 9 Hamiltonian cycle problems ranging in size from 1000 to 5000 nodes. Every instance of the problems contains a Hamiltonian cycle. Table 6.6 shows the excellent performance of the modified Lin-Kernighan algorithm for these problem instances.

Name	Success	$Trials_{min}$	$Trials_{avg}$	Gap_{avg}	Gap_{max}	$Time_{min}$	$Time_{avg}$
alb1000	10/10	1	1.0	0.000	0.000	0.0	0.1
alb2000	10/10	1	1.0	0.000	0.000	0.1	0.1
alb3000a	10/10	1	1.0	0.000	0.000	0.1	0.1
alb3000b	10/10	1	1.0	0.000	0.000	0.1	0.1
alb3000c	10/10	1	1.0	0.000	0.000	0.1	0.1
alb3000d	10/10	1	1.0	0.000	0.000	0.1	0.1
alb3000e	10/10	1	1.0	0.000	0.000	0.1	0.1
alb4000	10/10	1	1.0	0.000	0.000	0.1	0.1
alb5000	10/10	1	1.0	0.000	0.000	0.2	0.2

Table 6.6 Performance for the Hamiltonian cycle problems of TSPLIB

An interesting special Hamilton cycle problem is the so-called knight's-tour problem. A knight is to be moved around on a chessboard in such a way that all 64 squares are visited exactly once and the moves taken constitute a round trip on the board. Figure 6.5 depicts one solution of the problem. The two first moves are shown with arrows.

16	31	14	21	18	**1**	50	3
13	6	17	30	23	4	19	64
32	15	22	5	20	49	2	57
7	12	29	24	43	52	63	56
28	33	8	37	48	55	44	53
9	38	11	42	25	62	57	60
34	27	40	47	36	59	54	45
39	10	35	26	41	46	61	58

Figure 6.5 One solution of the knight's-tour problem

The problem is an instance of the general "leaper" problem: "Can a {r,s}-leaper, starting at any square of a mxn board, visit each square exactly once and return to its starting square" [52]. The knight's-tour problem is the {1,2}-leaper problem on a 8x8 board.

Table 6.7 shows the performance statistics for a few leaper problems. A small C-program, included in TSPLIB, was used for generating the leaper graphs. Edges belonging to the graph cost 0, and the remaining edges cost 1.

Problem	Success	$Trials_{min}$	$Trials_{avg}$	Gap_{avg}	Gap_{max}	$Time_{min}$	$Time_{avg}$
{1,2}, 8x8	100/100	1	1.0	0.000	0.000	0.0	0.0
{1,2}, 10x10	100/100	1	1.0	0.000	0.000	0.0	0.0
{1,2}, 20x20	100/100	1	2.1	0.000	0.000	0.0	0.0
{7,8}, 15x106	10/10	4	8.6	0.000	0.000	0.3	0.7
{6,7}, 13x76	100/100	1	1.1	0.000	0.000	0.3	0.5

Table 6.7 Performance for leaper problems (Variant 1)

The last of these problems has an optimal tour length of 18. All the other problems contain a Hamiltonian cycle, i.e., their optimum tour length is 0.

Table 6.8 shows the performance for the same problems, but now the edges of the leaper graph cost 100, and the remaining edges cost 101. Lin and Kernighan observed that knight's-tour problems with these edge costs were hard to solve for their algorithm [1].

Problem	Success	$Trials_{min}$	$Trials_{avg}$	Gap_{avg}	Gap_{max}	$Time_{min}$	$Time_{avg}$
{1,2}, 8x8	100/100	1	1.0	0.000	0.000	0.0	0.0
{1,2}, 10x10	100/100	1	1.0	0.000	0.000	0.0	0.0
{1,2}, 20x20	100/100	1	1.1	0.000	0.000	0.0	0.0
{7,8}, 15x106	10/10	3	10.0	0.000	0.000	0.4	1.0
{6,7}, 13x76	100/100	1	1.1	0.000	0.000	0.4	0.7

Table 6.8 Performance for leaper problems (Variant 2)

As seen the new implementation is almost unaffected.

6.4 Pathological problems

The Lin-Kernighan algorithm is not always as effective as it seems to be with "random" or "typical" problems. Papadimitriou and Steiglitz [53] have constructed a special class of instances of the TSP for which local search algorithms, such as the Lin-Kernighan algorithm, appears to be very ineffective. Papadimitriou and Steiglitz denote this class of problems as 'perverse'.

Each problem has n = 8k nodes. There is exactly one optimal tour with cost n, and there are $2^{k-1}(k-1)!$ tours that are next best, have arbitrary large cost, and cannot be improved by changing fewer than 3k edges. See [53] for a precise description of the constructed problems.

The difficulty of the problem class is illustrated in Table 6.9 showing the performance of the algorithm when subgradient optimization is left out. Note the results for the cases k = 3 and k = 5. Here the optimum was frequently found, whereas the implementation of the Lin-Kernighan algorithm by Papadimitriou and Steiglitz was unable to discover the optimum even once.

k	n	Success	$Trials_{min}$	$Trials_{avg}$	Gap_{avg}	Gap_{max}	$Time_{min}$	$Time_{avg}$
3	24	46/100	1	14.9	2479.5	8291.7	0.0	0.0
4	32	55/100	1	17.4	1732.8	6209.4	0.0	0.0
5	40	48/100	1	24.1	1430.4	4960.0	0.0	0.0
6	48	53/100	1	26.6	1045.1	4127.1	0.0	0.0
7	56	32/100	1	41.2	1368.2	3532.1	0.0	0.0
8	64	41/100	1	41.2	1040.2	3085.9	0.0	0.0
9	72	1/100	5	71.3	1574.1	2738.9	0.0	0.1
10	80	2/100	1	78.6	1700.5	4958.8	0.0	0.2

Table 6.9 Performance for perverse problems
(without subgradient optimization)

However, all problems are solved without any search, if subgradient optimization is used, provided that the initial period is sufficiently large (5*DIMENSION). Table 6.10 shows the time (in seconds) to find the optimal solutions by subgradient optimization.

k	n	Time
3	24	0.0
4	32	0.0
5	40	0.0
6	48	0.1
7	56	0.1
8	64	0.1
9	72	0.1
10	80	0.2

Table 6.10 Time to find optimal solutions for perverse problems
(with subgradient optimization)

7. Conclusions

This report has described a modified Lin-Kernighan algorithm and its implementation. In the development of the algorithm great emphasis was put on achieving high quality solutions, preferably optimal solutions, in a reasonable short time.

Achieving simplicity was of minor concern here. In comparison, the modified Lin-Kernighan algorithm of Mak and Morton [54] has a very simple algorithmic structure. However, this simplicity has been achieved with the expense of a reduced ability to find optimal solutions. Their algorithm does not even guarantee 2-opt optimality.

Computational experiments have shown that the new algorithm is highly effective. An optimal solution was obtained for all problems with a known optimum. This is remarkable, considering that the modified algorithm does not employ backtracking.

The running times were satisfactory for all test problems. However, since running time is approximately $O(n^{2.2})$, it may be impractical to solve very large problems. The current implementation seems to be feasible for problems with fewer than 100,000 cities (this depends, of course, of the available computer resources).

When distances are implicitly given, space requirements are $O(n)$. Thus, the space required for geometrical problems is linear. For such problems, not space, but run time, will usually be the limiting factor.

The effectiveness of the algorithm is primarily achieved through an efficient search strategy. The search is based on 5-opt moves restricted by carefully chosen candidate sets. The α-measure, based on sensitivity analysis of minimum spanning trees, is used to define candidate sets that are small, but large enough to allow excellent solutions to be found (usually the optimal solutions).

References

[1] S. Lin & B. W. Kernighan,
"An Effective Heuristic Algorithm for the Traveling-Salesman Problem",
Oper. Res. **21**, 498-516 (1973).

[2] E. L. Lawler, J. K. Lenstra, A. H. G. Rinnooy Kan & D. B. Shmoys (eds.),
The Traveling Salesman Problem: A Guided Tour of Combinatorial Optimization,
Wiley, New York (1985).

[3] G. B. Dantzig, D. R. Fulkerson & S. M. Johnson,
"Solution of a large-scale traveling-salesman problem",
Oper. Res., **2**, 393-410 (1954).

[4] J. D. C. Little, K. G. Murty, D. W. Sweeny & C. Karel,
"An algorithm for the traveling salesman problem",
Oper. Res., **11**, 972-989 (1963).

[5] R. M. Karp,
"Reducibility among Combinatorial Problems" *in*
R. E. Miller & J. W. Thatcher (eds.),
Complexity of Computer Computations,
Plenum Press, New York, 85-103 (1972).

[6] M. W. Padberg & G. Rinaldi,
"A branch-and-cut algorithm for the resolution of large-scale symmetric traveling salesman problems",
SIAM Review, **33**, 60-100 (1991).

[7] M. Grötchel & O. Holland,
"Solution of large scale symmetric travelling salesman problems",
Math. Programming, **51**, 141-202 (1991).

[8] D. Applegate, R. Bixby, V. Chvàtal & W. Cook,
"Finding cuts in the TSP (A preliminary report)",
DIMACS, Tech. Report 95-05 (1995).

[9] M. Bellmore & J. C. Malone,
"Pathology of traveling-salesman subtour-elimination algorithms",
Oper. Res., **19**, 278-307 (1972).

[10] D. L. Miller & J. F. Pekny,
"Exact solution of large asymmetric traveling salesman problems",
Science, **251**, 754-761 (1991).

[11] D. E. Rosenkrantz, R. E. Stearns & P. M. Lewis II,
"An analysis of several heuristics for the traveling salesman problem",
SIAM J. Comput., **6**, 563-581 (1977).

[12] G. Laporte,
"The Traveling Salesman Problem: An overview of exact and approximate algorithms",
Eur. J. Oper. Res., **59**, 231-247 (1992).

[13] G. Reinelt,
The Traveling Salesman: Computational Solutions for TSP Applications,
Lecture Notes in Computer Science, **840** (1994).

[14] I. I. Melamed, S. I. Sergeev & I. Kh. Sigal,
"The traveling salesman problem. Approximate algorithms",
Avtomat. Telemekh., **11**, 3-26 (1989).

[15] S. Lin,
"Computer Solutions of the Traveling Salesman Problem",
Bell System Tech. J., **44**, 2245-2269 (1965).

[16] N. Christofides & S. Eilon,
"Algorithms for Large-scale Travelling Salesman Problems",
Oper. Res. Quart., **23**, 511-518 (1972).

[17] D. S. Johnson,
"Local optimization and the traveling salesman problem",
Lecture Notes in Computer Science, **442**, 446-461 (1990).

[18] D. S. Johnson & L. A. McGeoch,
"The Traveling Salesman Problem: A Case Study in Local Optimization" *in*
E. H. L. Aarts & J. K. Lenstra (eds.),
"*Local Search in Combinatorial Optimization*",
Wiley, New York (1997).

[19] M. W. Padberg & G. Rinaldi,
"Optimization of a 532-city symmetric traveling salesman problem by branch and cut",
Oper. Res. Let., **6**, 1-7 (1987).

[20] M. Held & R. M. Karp,
"The Traveling-Salesman Problem and Minimum Spanning Trees",
Oper. Res., **18**, 1138-1162 (1970).

[21] M. Held & R. M. Karp,
"The Traveling-Salesman Problem and Minimum Spanning Trees: Part II",
Math. Programming, **1**, 16-25 (1971).

[22] R. C. Prim,
"Shortest connection networks and some generalizations",
Bell System Tech. J., **36**, 1389-1401 (1957).

[23] T. Volgenant & R. Jonker,
"The symmetric traveling salesman problem and edge exchanges i minimal 1-trees",
Eur. J. Oper. Res., **12**, 394-403 (1983).

[24] G. Carpaneto, M. Fichetti & P. Toth,
"New lower bounds for the symmetric travelling salesman problem",
Math. Programming, **45**, 233-254 (1989).

[25] M. Held, P. Wolfe & H.P. Crowder,
"Validation of subgradient optimization",
Math. Programming, **6**, 62-88 (1974).

[26] B.T. Poljak:
"A general method of solving extremum problems",
Soviet Math. Dokl., **8**, 593-597 (1967).

[27] H. P. Crowder,
"Computational improvements of subgradient optimization",
IBM Res. Rept. RC 4907, No. 21841 (1974).

[28] P. M. Camerini, L. Fratta & F. Maffioli,
"On improving relaxation methods by modified gradient techniques",
Math. Programming Study, **3**, 26-34 (1976).

[29] M. S. Bazaraa & H. D. Sherali,
"On the choice of step size in subgradient optimization",
Eur. J. Oper. Res., **7**, 380-388 (1981).

[30] T. Volgenant & R. Jonker,
"A branch and bound algorithm for the symmetric traveling salesman problem based on the 1-tree relaxation",
Eur. J. Oper. Res., **9**, 83-89 (1982).

[31] K. H. Helbig-Hansen & J. Krarup,
"Improvements of the Held-Karp algorithm for the symmetric traveling salesman problem",
Math. Programming, **7**, 87-96 (1974).

[32] W. R. Stewart, Jr.,
"Accelerated Branch Exchange Heuristics for Symmetric Traveling Salesman Problems",
Networks, **17**, 423-437 (1987).

[33] G. Reinelt,
"Fast Heuristics for Large Geometric Traveling Salesman Problems",
ORSA J. Comput., **2**, 206-217 (1992).

[34] A. Adrabinski & M. M. Syslo,
"Computational experiments with some approximation algorithms for the traveling salesman problem",
Zastos. Mat., **18**, 91-95 (1983).

[35] J. Perttunen,
"On the Significance of the Initial Solution in Travelling Salesman Heuristics",
J. Oper. Res. Soc., **45**, 1131-1140 (1994).

[36] G. Clarke & J. W. Wright,
"Scheduling of vehicles from a central depot to a number of delivery points",
Oper. Res., **12**, 568-581 (1964).

[37] N. Christofides,
"Worst Case Analysis of a New Heuristic for the Travelling Salesman Problem",
Report 388. Graduate School of Industrial Administration,
Carnegie-Mellon University, Pittsburg (1976).

[38] G. Reinelt,
"TSPLIB - A Traveling Salesman Problem Library",
ORSA J. Comput., **3-4**, 376-385 (1991).

[39] K-T. Mak & A. J. Morton,
"Distances between traveling salesman tours",
Disc. Appl. Math., **58**, 281-291 (1995).

[40] D. Applegate, R. E. Bixby, V. Chvàtal & W. Cook,
"Data Structures for the Lin-Kernighan Heuristic",
Talk presented at the TSP-Workshop, CRCP, Rice University (1990).

[41] L. Hárs,
"Reversible-Segment List",
Report No 89596-OR,
Forshunginstitut für Discrete Mathematik, Bonn (1989).

[42] F. Margot,
"Quick updates for p-opt TSP heuristics",
Oper. Res. Let., **11**, 45-46 (1992).

[43] M. L. Fredman, D. S. Johnson & L. A. McGeoch,
"Data Structures for Traveling Salesmen",
J. Algorithms., **16**, 432-479 (1995).

[44] J. L. Bentley,
"Fast Algorithms for Geometric Traveling Salesman Problems",
ORSA J. Comput., **4**, 347-411 (1992).

[45] J. L. Bentley,
"K-d trees for semidynamic point sets",
Sixth Annual ACM Symposium on Computational Geometry,
Berkely, CA, 187-197 (1990).

[46] O. Martin, S. W. Otto & E. W. Felten,
"Large-Step Markov Chains for the Traveling Salesman Problem",
J. Complex Systems, **5**, 219-224 (1991).

[47] S. Sahni & T. Gonzales,
"P-complete approximation algorithms",
J. Assoc. Comput. Mach., **23**, 555-565 (1976).

[48] J. L. Arthur & J. O. Frendewey,
"Generating Travelling-Salesman Problems with Known Optimal Tours",
J. Opl Res. Soc., **39**, 153-159 (1988).

[49] J. Beardswood & J. M. Hammersley,
"The shortest path through many points",
Proc. Cambridge Philos. Soc., **55**, 299-327 (1959).

[50] D. S. Johnson, L. A. McGeoch & E. E. Rothenberg,
"Asymptotic experimental analysis for the Held-Karp traveling salesman problem",
Proc. 7th ACM SIAM Symp. on Discrete Algorithms
Society of Industrial and Applied Mathematics, Philadelphia York (1996).

[51] R. Jonker & T. Volgenant,
"Transforming asymmetric into symmetric traveling salesman problems",
Oper. Res. Let., **2**, 161-163 (1983).

[52] D. E. Knuth,
"Leaper Graphs",
Math. Gaz., **78**, 274-297 (1994).

[53] C. H. Papadimitriou & K. Steiglitz,
"Some Examples of Difficult Traveling Salesman Problems",
Oper. Res., **26**, 434-443 (1978).

[54] K-T. Mak & A. J. Morton,
"A modified Lin-Kernighan traveling-salesman heuristic",
Oper. Res. Let., **13**, 127-132 (1993).
