# Supplementary material for: Solving the clustered traveling salesman problem via traveling salesman problem methods
Source: PeerJ Comput Sci. 2022 Jun 13;8:e972. doi: 10.7717/peerj-cs.972 (PMC9202618; doi:10.7717/peerj-cs.972)
Supplement: Supplemental Information 1 [file peerj-cs-08-972-s001.tgz › CLKH-1.0/LKH-2.0.7/DOC/TSPLIB_DOC.pdf]

TSPLIB 95

Gerhard Reinelt
Universität Heidelberg
Institut für Angewandte Mathematik
Im Neuenheimer Feld 294
D-69120 Heidelberg
Gerhard.Reinelt@IWR.Uni-Heidelberg.DE

TSPLIB is a library of sample instances for the TSP (and related problems) from various sources and of various types. Instances of the following problem classes are available.

Symmetric traveling salesman problem (TSP)
Given a set of n nodes and distances for each pair of nodes, find a roundtrip of minimal total length visiting each node exactly once. The distance from node i to node j is the same as from node j to node i.

Hamiltonian cycle problem (HCP)
Given a graph, test if the graph contains a Hamiltonian cycle or not.

Asymmetric traveling salesman problem (ATSP)
Given a set of n nodes and distances for each pair of nodes, find a roundtrip of minimal total length visiting each node exactly once. In this case, the distance from node i to node j and the distance from node j to node i may be different.

Sequential ordering problem (SOP)
This problem is an asymmetric traveling salesman problem with additional constraints. Given a set of n nodes and distances for each pair of nodes, find a Hamiltonian path from node 1 to node n of minimal length which takes given precedence constraints into account. Each precedence constraint requires that some node i has to be visited before some other node j.

Capacitated vehicle routing problem (CVRP)
We are given $n-1$ nodes, one depot and distances from the nodes to the depot, as well as between nodes. All nodes have demands which can be satisfied by the depot. For delivery to the nodes, trucks with identical capacities are available. The problem is to find tours for the trucks of minimal total length that satisfy the node demands without violating truck capacity constraint. The number of trucks is not specified. Each tour visits a subset of the nodes and starts and terminates at the depot. (Remark: In some data files a collection of alternate depots is given. A CVRP is then given by selecting one of these depots.)

Except, for the Hamiltonian cycle problems, all problems are defined on a complete graph and, at present, all distances are integer numbers. There is a possibility to require that certain edges appear in the solution of a problem.

1. The file format

Each file consists of a **specification part** and of a **data part**. The specification part contains information on the file format and on its contents. The data part contains explicit data.

1.1 The specification part

All entries in this section are of the form <*keyword*> : <*value*>, where <*keyword*> denotes an alphanumerical keyword and <*value*> denotes alphanumerical or numerical data. The terms <*string*>, <*integer*> and <*real*> denote character string, integer or real data, respectively. The order of specification of the keywords in the data file is arbitrary (in principle), but must be consistent, i.e., whenever a keyword is specified, all necessary information for the correct interpretation of the keyword has to be known. Below we give a list of all available keywords.

1.1.1 `NAME` : <*string*>

Identifies the data file.

1.1.2 `TYPE` : <*string*>

Specifies the type of the data. Possible types are

`TSP`	Data for a symmetric traveling salesman problem
`ATSP`	Data for an asymmetric traveling salesman problem
`SOP`	Data for a sequential ordering problem
`HCP`	Hamiltonian cycle problem data
`CVRP`	Capacitated vehicle routing problem data
`TOUR`	A collection of tours

1.1.3 `COMMENT` : <*string*>

Additional comments (usually the name of the contributor or creator of the problem instance is given here).

1.1.4 `DIMENSION` : <*integer*>

For a TSP or ATSP, the dimension is the number of its nodes. For a CVRP, it is the total number of nodes and depots. For a TOUR file it is the dimension of the corresponding problem.

1.1.5 `CAPACITY` : <*integer*>

Specifies the truck capacity in a CVRP.

1.1.6 `EDGE_WEIGHT_TYPE` : <*string*>

Specifies how the edge weights (or distances) are given. The values are

`EXPLICIT`	Weights are listed explicitly in the corresponding section
`EUC_2D`	Weights are Euclidean distances in 2-D
`EUC_3D`	Weights are Euclidean distances in 3-D

MAX_2D	Weights are maximum distances in 2-D
MAX_3D	Weights are maximum distances in 3-D
MAN_2D	Weights are Manhattan distances in 2-D
MAN_3D	Weights are Manhattan distances in 3-D
CEIL_2D	Weights are Euclidean distances in 2-D rounded up
GEO	Weights are geographical distances
ATT	Special distance function for problems att48 and att532
XRAY1	Special distance function for crystallography problems (Version 1)
XRAY2	Special distance function for crystallography problems (Version 2)
SPECIAL	There is a special distance function documented elsewhere

1.1.7 EDGE_WEIGHT_FORMAT : <*string*>

Describes the format of the edge weights if they are given explicitly. The values are

FUNCTION	Weights are given by a function (see above)
FULL_MATRIX	Weights are given by a full matrix
UPPER_ROW	Upper triangular matrix (row-wise without diagonal entries)
LOWER_ROW	Lower triangular matrix (row-wise without diagonal entries)
UPPER_DIAG_ROW	Upper triangular matrix (row-wise including diagonal entries)
LOWER_DIAG_ROW	Lower triangular matrix (row-wise including diagonal entries)
UPPER_COL	Upper triangular matrix (column-wise without diagonal entries)
LOWER_COL	Lower triangular matrix (column-wise without diagonal entries)
UPPER_DIAG_COL	Upper triangular matrix (column-wise including diagonal entries)
LOWER_DIAG_COL	Lower triangular matrix (column-wise including diagonal entries)

1.1.7 EDGE_DATA_FORMAT : <*string*>

Describes the format in which the edges of a graph are given, if the graph is not complete. The values are

EDGE_LIST	The graph is given by an edge list
ADJ_LIST	The graph is given as an adjacency list

1.1.9 NODE_COORD_TYPE : <*string*>

Specifies whether coordinates are associated with each node (which, for example may be used for either graphical display or distance computations). The values are

TWOD_COORDS	Nodes are specified by coordinates in 2-D
THREED_COORDS	Nodes are specified by coordinates in 3-D
NO_COORDS	The nodes do not have associated coordinates

The default value is NO_COORDS.

1.1.10 DISPLAY_DATA_TYPE : <*string*>

Specifies how a graphical display of the nodes can be obtained. The values are

COORD_DISPLAY	Display is generated from the node coordinates
TWOD_DISPLAY	Explicit coordinates in 2-D are given
NO_DISPLAY	No graphical display is possible

The default value is COORD_DISPLAY if node coordinates are specified and NO_DISPLAY otherwise.

1.1.11 `EOF` :

Terminates the input data. This entry is optional.

1.2 The data part

Depending on the choice of specifications some additional data may be required. These data are given in corresponding data sections following the specification part. Each data section begins with the corresponding keyword. The length of the section is either implicitly known from the format specification, or the section is terminated by an appropriate end-of-section identifier.

1.2.1 `NODE_COORD_SECTION` :

Node coordinates are given in this section. Each line is of the form

<*integer*> <*real*> <*real*>

if `NODE_COORD_TYPE` is `TWOD_COORDS`, or

<*integer*> <*real*> <*real*> <*real*>

if `NODE_COORD_TYPE` is `THREED_COORDS`. The integers give the number of the respective nodes. The real numbers give the associated coordinates.

1.2.2 `DEPOT_SECTION` :

Contains a list of possible alternate depot nodes. This list is terminated by a -1.

1.2.3 `DEMAND_SECTION` :

The demands of all nodes of a CVRP are given in the form (per line)

<*integer*> <*integer*>

The first integer specifies a node number, the second its demand. The depot nodes must also occur in this section. Their demands are 0.

1.2.4 `EDGE_DATA_SECTION` :

Edges of a graph are specified in either of the two formats allowed in the `EDGE_DATA_FORMAT` entry. If the type is `EDGE_LIST`, then the edges are given as a sequence of lines of the form

<*integer*> <*integer*>

each entry giving the terminal nodes of some edge. The list is terminated by a -1.
If the type is `ADJ_LIST`, the section consists of a list of adjacency lists for nodes. The adjacency list of a node x is specified as

<*integer*> <*integer*> ... <*integer*> -1

where the first integer gives the number of node x and the following integers (terminated by -1) the numbers of nodes adjacent to x. The list of adjacency lists is terminated by an additional -1.

1.2.5 FIXED_EDGES_SECTION :

In this section, edges are listed that are required to appear in each solution to the problem. The edges to be fixed are given in the form (per line)

<*integer*> <*integer*>

meaning that the edge (arc) from the first node to the second node has to be contained in a solution. This section is terminated by a −1.

1.2.6 DISPLAY_DATA_SECTION :

If DISPLAY_DATA_TYPE is TWOD_DISPLAY, the 2-dimensional coordinates from which a display can be generated are given in the form (per line)

<*integer*> <*real*> <*real*>

The integers specify the respective nodes and the real numbers give the associated coordinates.

1.2.7 TOUR_SECTION :

A collection of tours is specified in this section. Each tour is given by a list of integers giving the sequence in which the nodes are visited in this tour. Every such tour is terminated by a −1. An additional −1 terminates this section.

1.2.8 EDGE_WEIGHT_SECTION :

The edge weights are given in the format specified by the EDGE_WEIGHT_FORMAT entry. At present, all explicit data is integral and is given in one of the (self-explanatory) matrix formats. with implicitly known lengths.

2. The distance functions

For the various choices of EGDE_WEIGHT_TYPE, we now describe the computations of the repsective distances. In each case we give a (simplified) C-implementation for computing the distances from the input coordinates. All computations involving floating-point numbers are carried out in double precision arithmetic. The integers are assumed to be represented in 32-bit words. Since distances are required to be integral, we round to the nearest integer (in most cases). Below we have used the rounding function "nint".

2.1 Euclidean distance (L_2-metric)

For edge weight type EUC_2D and EUC_3D, floating point coordinates must be specified for each node. Let x[i], y[i], and z[i] be the coordinates of node i.
In the 2-dimensional case the distance between two points i and j is computed as follows:

```
xd = x[i] - x[j];
yd = y[i] - y[j];
dij = nint( sqrt( xd*xd + yd*yd) );
```

In the 3-dimensional case we have:

```
xd = x[i] - x[j];
yd = y[i] - y[j];
zd = z[i] - z[j];
dij = nint( sqrt( xd*xd + yd*yd + zd*zd) );
```

where sqrt is the C square root function.

2.2 Manhattan distance (L_1-metric)

Distances are given as Manhattan distances if the edge weight type is MAN_2D or MAN_3D. They are computed as follows.
2-dimensional case:

```
xd = abs( x[i] - x[j] );
yd = abs( y[i] - y[j] );
dij = nint( xd + yd );
```

3-dimensional case:

```
xd = abs( x[i] - x[j] );
yd = abs( y[i] - y[j] );
zd = abs( z[i] - z[j] );
dij = nint( xd + yd + zd );
```

2.3 Maximum distance (L_∞-metric)

Maximum distances are computed if the edge weight type is MAX_2D or MAX_3D.
2-dimensional case:

```
xd = abs( x[i] - x[j] );
yd = abs( y[i] - y[j] );
dij = max( nint( xd ), nint( yd ) );
```

3-dimensional case:

```
xd = abs( x[i] - x[j] );
yd = abs( y[i] - y[j] );
zd = abs( z[i] - z[j] );
dij = max( nint( xd ), nint( yd ), nint( zd ) );
```

2.4 Geographical distance

If the traveling salesman problem is a geographical problem, then the nodes correspond to points on the earth and the distance between two points is their distance on the idealized sphere with radius 6378.388 kilometers. The node coordinates give the geographical latitude and longitude of the corresponding point on the earth. Latitude and longitude are given in the form `DDD.MM` where `DDD` are the degrees and `MM` the minutes. A positive latitude is assumed to be "North", negative latitude means "South". Positive longitude means "East", negative latitude is assumed to be "West". For example, the input coordinates for Augsburg are 48.23 and 10.53, meaning $48^o23'$ North and $10^o53'$ East.
Let `x[i]` and `y[i]` be coordinates for city i in the above format. First the input is converted to geographical latitude and longitude given in radians.

```
PI = 3.141592;

deg = nint( x[i] );
min = x[i] - deg;
latitude[i] = PI * (deg + 5.0 * min / 3.0 ) / 180.0;
deg = nint( y[i] );
min = y[i] - deg;
longitude[i] = PI * (deg + 5.0 * min / 3.0 ) / 180.0;
```

The distance between two different nodes i and j in kilometers is then computed as follows:

```
RRR = 6378.388;

q1 = cos( longitude[i] - longitude[j] );
q2 = cos( latitude[i] - latitude[j] );
q3 = cos( latitude[i] + latitude[j] );
dij = (int) ( RRR * acos( 0.5*((1.0+q1)*q2 - (1.0-q1)*q3) ) + 1.0);
```

The function "`acos`" is the inverse of the cosine function.

2.5 Pseudo-Euclidean distance

The edge weight type `ATT` corresponds to a special "pseudo-Euclidean" distance function. Let `x[i]` and `y[i]` be the coordinates of node i. The distance between two points i and j is computed as follows:

```
xd = x[i] - x[j];
yd = y[i] - y[j];
rij = sqrt( (xd*xd + yd*yd) / 10.0 );
tij = nint( rij );
if (tij<rij) dij = tij + 1;
else dij = tij;
```

2.6 Ceiling of the Euclidean distance

The edge weight type CEIL_2D requires that the 2-dimensional Euclidean distances is rounded up to the next integer.

2.7 Distance for crystallography problems

We have included into TSPLIB the crystallography problems as described in [1]. These problems are not explicitly given but subroutines are provided to generate the 12 problems mentioned in this reference and subproblems thereof (see section 3.2).
To compute distances for these problems the movement of three motors has to be taken into consideration. There are two types of distance functions: one that assumes equal speed of the motors (XRAY1) and one that uses different speeds (XRAY2). The corresponding distance functions are given as FORTRAN implementations (files deq.f, resp. duneq.f) in the distribution file.
For obtaining integer distances, we propose to multiply the distances computed by the original subroutines by 100.0 and round to the nearest integer.
We list our modified distance function for the case of equal motor speeds in the FORTRAN version below.

```
  INTEGER FUNCTION ICOST(V,W)
  INTEGER V,W
  DOUBLE PRECISION DMIN1,DMAX1,DABS
  DOUBLE PRECISION DISTP,DISTC,DISTT,COST
  DISTP=DMIN1(DABS(PHI(V)-PHI(W)),DABS(DABS(PHI(V)-PHI(W))-360.0E+0))
  DISTC=DABS(CHI(V)-CHI(W))
  DISTT=DABS(TWOTH(V)-TWOTH(W))
  COST=DMAX1(DISTP/1.00E+0,DISTC/1.0E+0,DISTT/1.00E+0)
C  *** Make integral distances ***
  ICOST=AINT(100.0E+0*COST+0.5E+0)
  RETURN
  END
```

The numbers PHI(), CHI(), and TWOTH() are the respective x-, y-, and z-coordinates of the points in the generated traveling salesman problems. Note, that TSPLIB95 contains only the original distance computation without the above modification.

2.7 Verification

To verify correctness of the distance function implementations we give the length of some "canonical" tours $1, 2, 3, \ldots, n$.
The canonical tours for pcb442, gr666, and att532 have lengths 221 440, 423 710, and 309 636, respectively.
The canonical tour for the problem xray14012 (the 8th problem considered in [21]) with distance XRAY1 has length 15 429 219. With distance XRAY2 it has the length 12 943 294.

3. Description of the library files

In this section we give a list of all problem instances that are currently available together with information on the length of optimal tours or lower and upper bounds for this length (if available).

3.1 Symmetric traveling salesman problems

The TSP instances are contained in directory `tsp`. Table 1 gives the problem names along with number of cities, problem type, and known lower and upper bounds for the optimal tour length (a single number indicating that the optimal length is known). The entry `MATRIX` indicates that the data is given in one of the matrix formats of 1.1.7. The names of the corresponding data files are obtained by appending the suffix "`.tsp`" to the problem name. Some optimal tours are also provided. The corresponding files have names with suffix "`.opt.tour`".

Name	#cities	Type	Bounds
a280	280	EUC_2D	**2579**
ali535	535	GEO	**202310**
att48	48	ATT	**10628**
att532	532	ATT	**27686**
bayg29	29	GEO	**1610**
bays29	29	GEO	**2020**
berlin52	52	EUC_2D	**7542**
bier127	127	EUC_2D	**118282**
brazil58	58	MATRIX	**25395**
brd14051	14051	EUC_2D	[468942,469445]
brg180	180	MATRIX	**1950**
burma14	14	GEO	**3323**
ch130	130	EUC_2D	**6110**
ch150	150	EUC_2D	**6528**
d198	198	EUC_2D	**15780**
d493	493	EUC_2D	**35002**
d657	657	EUC_2D	**48912**
d1291	1291	EUC_2D	**50801**
d1655	1655	EUC_2D	**62128**
d2103	2103	EUC_2D	[79952,80450]
d15112	15112	EUC_2D	[1564590,1573152]
d18512	18512	EUC_2D	[644650,645488]
dantzig42	42	MATRIX	**699**
dsj1000	1000	CEIL_2D	**18659688**
eil51	51	EUC_2D	**426**
eil76	76	EUC_2D	**538**
eil101	101	EUC_2D	**629**

Table 1 Symmetric traveling salesman problems (Part I)

Name	#cities	Type	Bounds
fl417	417	EUC_2D	**11861**
fl1400	1400	EUC_2D	**20127**
fl1577	1577	EUC_2D	[22204,22249]
fl3795	3795	EUC_2D	[28723,28772]
fnl4461	4461	EUC_2D	**182566**
fri26	26	MATRIX	**937**
gil262	262	EUC_2D	**2378**
gr17	17	MATRIX	**2085**
gr21	21	MATRIX	**2707**
gr24	24	MATRIX	**1272**
gr48	48	MATRIX	**5046**
gr96	96	GEO	**55209**
gr120	120	MATRIX	**6942**
gr137	137	GEO	**69853**
gr202	202	GEO	**40160**
gr229	229	GEO	**134602**
gr431	431	GEO	**171414**
gr666	666	GEO	**294358**
hk48	48	MATRIX	**11461**
kroA100	100	EUC_2D	**21282**
kroB100	100	EUC_2D	**22141**
kroC100	100	EUC_2D	**20749**
kroD100	100	EUC_2D	**21294**
kroE100	100	EUC_2D	**22068**
kroA150	150	EUC_2D	**26524**
kroB150	150	EUC_2D	**26130**
kroA200	200	EUC_2D	**29368**
kroB200	200	EUC_2D	**29437**
lin105	105	EUC_2D	**14379**
lin318	318	EUC_2D	**42029**
linhp318	318	EUC_2D	**41345**
nrw1379	1379	EUC_2D	**56638**
p654	654	EUC_2D	**34643**
pa561	561	MATRIX	**2763**
pcb442	442	EUC_2D	**50778**
pcb1173	1173	EUC_2D	**56892**
pcb3038	3038	EUC_2D	**137694**
pla7397	7397	CEIL_2D	**23260728**
pla33810	33810	CEIL_2D	[65913275,66116530]
pla85900	85900	CEIL_2D	[141904862,142487006]

Table 1 Symmetric traveling salesman problems (Part II)

Name	#cities	Type	Bounds
pr76	76	EUC_2D	**108159**
pr107	107	EUC_2D	**44303**
pr124	124	EUC_2D	**59030**
pr136	136	EUC_2D	**96772**
pr144	144	EUC_2D	**58537**
pr152	152	EUC_2D	**73682**
pr226	226	EUC_2D	**80369**
pr264	264	EUC_2D	**49135**
pr299	299	EUC_2D	**48191**
pr439	439	EUC_2D	**107217**
pr1002	1002	EUC_2D	**259045**
pr2392	2392	EUC_2D	**378032**
rat99	99	EUC_2D	**1211**
rat195	195	EUC_2D	**2323**
rat575	575	EUC_2D	**6773**
rat783	783	EUC_2D	**8806**
rd100	100	EUC_2D	**7910**
rd400	400	EUC_2D	**15281**
rl1304	1304	EUC_2D	**252948**
rl1323	1323	EUC_2D	**270199**
rl1889	1889	EUC_2D	**316536**
rl5915	5915	EUC_2D	[565040,565530]
rl5934	5934	EUC_2D	[554070,556045]
rl11849	11849	EUC_2D	[920847,923368]
si175	175	MATRIX	**21407**
si535	535	MATRIX	**48450**
si1032	1032	MATRIX	**92650**
st70	70	EUC_2D	**675**
swiss42	42	MATRIX	**1273**
ts225	225	EUC_2D	**126643**
tsp225	225	EUC_2D	**3919**
u159	159	EUC_2D	**42080**
u574	574	EUC_2D	**36905**
u724	724	EUC_2D	**41910**
u1060	1060	EUC_2D	**224094**
u1432	1432	EUC_2D	**152970**
u1817	1817	EUC_2D	**57201**
u2152	2152	EUC_2D	**64253**
u2319	2319	EUC_2D	**234256**
ulysses16	16	GEO	**6859**
ulysses22	22	GEO	**7013**
usa13509	13509	EUC_2D	[19947008,19982889]
vm1084	1084	EUC_2D	**239297**
vm1748	1748	EUC_2D	**336556**

Table 1 Symmetric traveling salesman problems (Part III)

Crystallography problems

In the file `xray.problems` in directory `tsp` we distribute the routines written by Bland and Shallcross and the necessary data to generate the crystallography problems discussed in [1]. The file `xray.problems` is one file into which the single files mentioned in the sequel have been merged. These single files have to be extracted from `xray.problems` using an editor. The following original files are provided

read.me	deq.f	duneq.f	daux.f	gentsp.f
a.data	b.data	d.data	e.data	f.data

In addition we have included specially prepared data files to generate the 12 problems mentioned in [1]. The files have the names `xray1.data` through `xray12.data`.
Using these data files 12 symmetric TSPs can be generated using the program `gentsp.f`. We propose to name the respective problem instances `xray4472`, `xray2950`, `xray7008`, `xray2762`, `xray6922`, `xray9070`, `xray5888`, `xray14012`, `xray5520`, `xray13804`, `xray14464`, and `xray13590`.

To verify the correct use of the generating routines we list part of the file `xray14012.tsp`.

```
NAME : xray14012
COMMENT : Crystallography problem 8 (Bland/Shallcross)
TYPE : TSP
DIMENSION : 14012
EDGE_WEIGHT_TYPE : XRAY2
NODE_COORD_SECTION

         1    -91.802854544029   -6.4097888697337    176.39830490027
         2    -87.715643397938   -6.4659384343446    165.56800324542
         3    -83.587211962870   -6.4895404648110    163.53828545043
         4    -79.460007412434   -6.4797580053949    165.86438271158
       :
       :
     14009    100.539992581837    6.4797580053949    165.86438271158
     14010     96.412788031401    6.4895404648110    163.53828545043
     14011     92.284356596333    6.4659384343446    165.56800324542
     14012     88.197145450242    6.4097888697337    176.39830490027
```

3.2 Hamiltonian cycle problems

Instances of the Hamiltonian cycle problem are contained in the directory `hcp`. At present, we have the data files

alb1000.hcp	alb3000b.hcp	alb3000e.hcp	alb2000.hcp	alb3000c.hcp
alb4000.hcp	alb3000a.hcp	alb3000d.hcp	alb5000.hcp	

Every instance contains a Hamiltonian cycle which is given in the corresponding `.opt.tour` file. In problem instance `alb4000` two edges are fixed.
In addition to these files, the directory contains the C-program `tspleap.c` by M. Jünger and G. Rinaldi. This program can be used to generate TSP instances (in TSPLIB format)

originating from the problem of deciding whether an (r,s)-leaper on a mxn chess board can start at some square of the board, visit each square exactly once, and return to its starting square. A detailed documentation is given in the file `tspleap.c`.

3.3 Asymmetric traveling salesman problems

Table 2 lists the ATSP instances (in directory `atsp`) together with their optimal solution values. The names of the corresponding data files are obtained by appending the suffix "`.atsp`" to the problem name. The data files for problems `ftv90`, `ftv100`, `ftv110`, `ftv120`, `ftv130`, `ftv140`, `ftv150`, and `ftv160` are not present. These instances are obtained from `ftv170`. E.g., `ftv120` is the subproblem of `ftv170` defined by the first 121 nodes, `ftv130` is defined by the first 131 nodes, etc.

Name	#cities	Type	Optimum
br17	17	MATRIX	**39**
ft53	53	MATRIX	**6905**
ft70	70	MATRIX	**38673**
ftv33	34	MATRIX	**1286**
ftv35	36	MATRIX	**1473**
ftv38	39	MATRIX	**1530**
ftv44	45	MATRIX	**1613**
ftv47	48	MATRIX	**1776**
ftv55	56	MATRIX	**1608**
ftv64	65	MATRIX	**1839**
ftv70	71	MATRIX	**1950**
ftv90	91	MATRIX	**1579**
ftv100	101	MATRIX	**1788**
ftv110	111	MATRIX	**1958**
ftv120	121	MATRIX	**2166**
ftv130	131	MATRIX	**2307**
ftv140	141	MATRIX	**2420**
ftv150	151	MATRIX	**2611**
ftv160	161	MATRIX	**2683**
ftv170	171	MATRIX	**2755**
kro124	100	MATRIX	**36230**
p43	43	MATRIX	**5620**
rbg323	323	MATRIX	**1326**
rbg358	358	MATRIX	**1163**
rbg403	403	MATRIX	**2465**
rbg443	443	MATRIX	**2720**
ry48p	48	MATRIX	**14422**

Table 2 Asymmetric traveling salesman problems

3.4 Sequential ordering problems

Every instance of a sequential ordering problem is given by a full matrix C of the following kind. If node i has to precede node j, then C_{ji} is set to -1. C is assumed to be transitively closed with respect to precedences, i.e., if i has to precede j and j has to precede k, then

it is implied that i has to precede k and, therefore, also C_{ki} has to be set to -1. Because we require, that node 1 is the first node and node n is the last node in each feasible path, a SOP problem instance always has $C_{i1} = -1$, for all $i = 2, \ldots, n$, and $C_{nj} = -1$, for all $j = 1, \ldots, n-1$. The entry C_{1n} is set to infinity. All other entries of C are nonnegative integer values.

Name	#nodes	#prec.	Type	Bounds
ESC07	9	6	MATRIX	**2125**
ESC11	13	3	MATRIX	**2075**
ESC12	14	7	MATRIX	**1675**
ESC25	27	9	MATRIX	**1681**
ESC47	49	10	MATRIX	**1288**
ESC63	65	95	MATRIX	**62**
ESC78	80	77	MATRIX	**18230**
br17.10	17	10	MATRIX	**55**
br17.12	17	12	MATRIX	**55**
ft53.1	54	12	MATRIX	[7438,7570]
ft53.2	54	25	MATRIX	[7630,8335]
ft53.3	54	48	MATRIX	[9473,10935]
ft53.4	54	63	MATRIX	**14425**
ft70.1	71	17	MATRIX	**39313**
ft70.2	71	35	MATRIX	[39739,41778]
ft70.3	71	68	MATRIX	[41305,44732]
ft70.4	71	86	MATRIX	[52269,53882]
kro124p.1	101	25	MATRIX	[37722,42845]
kro124p.2	101	49	MATRIX	[38534,45848]
kro124p.3	101	97	MATRIX	[40967,55649]
kro124p.4	101	131	MATRIX	[64858,80753]
p43.1	44	9	MATRIX	**27990**
p43.2	44	20	MATRIX	[28175,28330]
p43.3	44	37	MATRIX	[28366,28680]
p43.4	44	50	MATRIX	[69569,82960]
prob42	42	10	MATRIX	**243**
prob100	100	41	MATRIX	[1024,1385]
rbg048a	50	192	MATRIX	**351**
rbg050c	52	256	MATRIX	**467**
rbg109a	111	622	MATRIX	**1038**
rbg150a	152	952	MATRIX	[1748,1750]
rbg174a	176	1113	MATRIX	**2053**
rbg253a	255	1721	MATRIX	[2928,2987]
rbg323a	325	2412	MATRIX	[3136,3221]
rbg341a	343	2542	MATRIX	[2543,2854]
rbg358a	360	3239	MATRIX	[2518,2758]
rbg378a	380	3069	MATRIX	[2761,3142]
ry48p.1	49	11	MATRIX	[15220,15935]
ry48p.2	49	23	MATRIX	[15524,17071]
ry48p.3	49	42	MATRIX	[18156,20051]
ry48p.4	49	58	MATRIX	[29967,31446]

Table 3 Sequential ordering problems

Table 3 lists the SOP instances (in directory sop) together with their known lower and upper bounds for the optimal path length. The names of the corresponding data files are obtained by appending the suffix ".sop" to the problem name.

3.5 Capacitated vehicle routing problems

Data for capacitated vehicle routing problems is contained in the directory vrp. Data files have suffix ".vrp". At present, we have the data files

att48.vrp	eil30.vrp	eil7.vrp	eilB76.vrp	eil13.vrp
eil31.vrp	eilA101.vrp	eilC76.vrp	eil22.vrp	eil33.vrp
eilA76.vrp	eilD76.vrp	eil23.vrp	eil51.vrp	eilB101.vrp
gil262.vrp				

Various problems can be defined on these data sets, e.g., depending on whether the number of vehicles is fixed, so we do not list optimal solutions here. Some values are given in the data files themselves.

3.6 Further special files

In addition to the data and solution files, the following special files are contained in the library.

TSPLIB_VERSION:	Gives the current version of the library
README:	A short information on TSPLIB
DOC.PS:	Description of TSPLIB (PostScript)

4. Remarks

1. The problem lin318 is originally a Hamiltonian path problem. One obtains this problem by adding the additional requirement that the edge from 1 to 214 is contained in the tour. The data is given in linhp318.tsp.

2. Some data sets are referred to by different names in the literature. Below we give the corresponding names used in [3] and [2].

TSPLIB	[3]	[2]	TSPLIB	[3]	[2]
att48	ATT048	-	att532	ATT532	-
dantzig42	-	42	eil101	EIL10	-
eil51	EIL08	-	eil76	EIL09	-
gil262	GIL249	-	gr120	-	120
gr137	GH137	137	gr202	GH202	202
gr229	GH229	229	gr431	GH431	431
gr666	GH666	666	gr96	GH096	96
hk48	-	48H	kroA100	KRO124	100A
kroB100	KRO125	100B	kroC100	KRO126	100C
kroD100	KRO127	100D	kroE100	KRO128	100E

kroA150	KRO30	-	kroB150	KRO31	-
kroA200	KRO32	-	kroB200	KRO33	-
lin105	LK105	-	lin318	LK318	-
linhp318	LK318P	-	pr1002	TK1002	-
pr107	TK107	-	pr124	TK124	-
pr136	TK136	-	pr144	TK144	-
pr152	TK152	-	pr226	TK226	-
pr2392	TK2392	-	pr264	TK264	-
pr299	TK299	-	pr439	TK439	-
pr76	TK076	-	st70	KR0070	70

3. Some vehicle routing problems are also available in a TSP version. Here the depots are just treated as normal nodes. The problem gil262 originally contained two identical nodes, of which one was eliminated.

4. Potential contributors to this library should provide their data files in appropriate format and contact

Gerhard Reinelt
Institut für Angewandte Mathematik, Universität Heidelberg
Im Neuenheimer Feld 294, D-69120 Heidelberg
Germany
Tel (6221) 56 3171
Fax (6221) 56 5634
E-Mail Gerhard.Reinelt@IWR.Uni-Heidelberg.DE

5. Informations on new bounds or optimal solutions for library problems as well as references to computational studies (to be included in the list of references) are also appreciated.

5. Access

TSPLIB is available at various locations.

Interdisziplinäres Zentrum für Wissenschaftliches Rechnen (IWR), Heidelberg

World Wide Web:

`http://www.iwr.uni-heidelberg.de/iwr/comopt/soft/TSPLIB95/TSPLIB.html`

Konrad-Zuse-Zentrum für Informationstechnik (ZIB), Berlin

World Wide Web:

`ftp://elib.zib-berlin.de/pub/mp-testdata/tsp/index.html`

For further information on how to use the electronic library facilities at ZIB send a mail just containing “help” to elib@ZIB-Berlin.de.

Center for Research on Parallel Computation (CRPC), Houston

Gopher:

`gopher://softlib.rice.edu/11/softlib/tsplib`

Anonymous ftp:

`ftp://softlib.rice.edu/pub/tsplib`

References

1. R.E. Bland & D.F. Shallcross (1989). *Large Traveling Salesman Problems Arising from Experiments in X-ray Crystallography: A Preliminary Report on Computation,* Operations Research Letters *8*, 125–128.
2. M. Grötschel & O. Holland (1991). *Solution of Large-Scale Symmetric Travelling Salesman Problems,* Mathematical Programming *51*, 141–202.
3. M.W. Padberg & G. Rinaldi (1991). *A Branch & Cut Algorithm for the Resolution of Large-scale Symmetric Traveling Salesman Problems,* SIAM Review *33*, 60–100.
